# Dynamics of Singlet Oxygen-Triggered, RONS-Based Apoptosis Induction after Treatment of Tumor Cells with Cold Atmospheric Plasma or Plasma-Activated Medium

**DOI:** 10.1038/s41598-019-50329-3

**Published:** 2019-09-26

**Authors:** Georg Bauer, Dominika Sersenová, David B. Graves, Zdenko Machala

**Affiliations:** 1 Institute of Virology, Medical Center - University of Freiburg, Freiburg, Germany; 2 Faculty of Medicine, University of Freiburg, Freiburg, Germany; 3 Division of Environmental Physics, Faculty of Mathematics, Physics and Informatics, Comenius University, Bratislava, Slovakia; 4 Department of Chemical and Biomolecular Engineering, University of California at Berkeley, Berkeley, California 94720 USA

**Keywords:** Biochemistry, Cancer, Cell biology, Chemical biology, Oncology

## Abstract

Treatment of tumor cells with cold atmospheric plasma (CAP) or with plasma-activated medium (PAM) leads to a biochemical imprint on these cells. This imprint is mediated by primary singlet oxygen, which is mainly generated through the interaction between CAP-derived H_2_O_2_ and NO_2_^−^. This imprint is induced with a low efficiency as local inactivation of a few membrane-associated catalase molecules. As sustained generation of secondary singlet oxygen by the tumor cells is activated at the site of the imprint, a rapid bystander effect-like spreading of secondary singlet oxygen generation and catalase inactivation within the cell population is thus induced. This highly dynamic process is essentially driven by NOX1 and NOS of the tumor cells, and finally leads to intercellular RONS-driven apoptosis induction. This dynamic process can be studied by kinetic analysis, combined with the use of specific inhibitors at defined time intervals. Alternatively, it can be demonstrated and quantified by transfer experiments, where pretreated cells are mixed with untreated cells and bystander signaling is determined. These studies allow to conclude that the specific response of tumor cells to generate secondary singlet oxygen is the essential motor for their self-destruction, after a singlet oxygen-mediated triggering process by CAP or PAM.

## Introduction

Plasma medicine^1,2^ is coupling plasma physics and chemistry, biochemistry, and biology as rational basis for various fascinating medical applications of plasma^3^. Cold atmospheric plasma (CAP) and plasma activated medium (PAM) show impressive antitumor effects *in vitro* and *in vivo*^4–24^.

Most studies concluded that CAP- and PAM-mediated apoptosis induction acts selectively on tumor cells^25–36^. There is a general consent that RONS are centrally involved in CAP- and PAM-mediated antitumor effects^5,37^. The exact mechanism is the subject of an ongoing discussion.

The gaseous and liquid phase of CAP contains electrons, photons, superoxide anions (O_2_^●−^), hydroperoxyl radicals (HO_2_^●^), hydrogen peroxide (H_2_O_2_), hydroxyl radicals (^●^OH), atomic oxygen (O), singlet oxygen (^1^O_2_), ozone (O_3_), nitric oxide (^●^NO), nitrogen dioxide (^●^NO_2_), peroxynitrite (ONOO^−^), nitrite (NO_2_^−^), nitrate (NO_3_^−^), dichloride radicals (Cl_2_^●−^) and hypochloride anions (OCl^−^)^3,16,37,38^. Due to the high reactivity and short live time of most CAP-derived species, PAM (as well as other plasma-activated liquids) essentially contains only H_2_O_2_, NO_2_^−^ and NO_3_^−^ ^33,39–41^. It is intriguing that a liquid with a RONS composition of such an apparently low complexity can trigger impressive antitumor effects in many tumor systems *in vitro* and *in vivo*, as shown by many groups^5,14,30,33–36,40,42–51^, and reviewed by Yan *et al*.^52^. Whereas Koensgen *et al*.^50^ and Canal *et al*.^35^ found equivalent antitumor effects of CAP and PAM *in vitro*, Saadati *et al*.^53^ reported on a stronger effect of CAP compared to PAM *in vivo*. Yan *et al*.^54^ reported that CAP, but not PAM, triggered H_2_O_2_ generation from tumor cells. Several groups reported on selective effects of PAM towards tumor cells^30,33–36^. Ikeda *et al*.^51^ presented evidence that PAM kills human cancer-initiating cells. PAM is not only effective in classical 2 D cell cultures, but also affects 3D multicellular tumor spheroids^55^ *in vitro* and is effective *in vivo*^36,42,46,53^. There is very strong evidence that RONS contained in PAM are responsible for apoptosis induction in tumor cells, and that PAM treatment also triggers a RONS response of the target cells^5,33,40,43,46,48^. As H_2_O_2_ seems to play a central role for PAM-mediated antitumor effects, and as tumor cells express high concentrations of aquaporins in their membrane^56^, Yan *et al*.^57,58^ proposed that an increased influx of H_2_O_2_ through aquaporins determines the selective effect of PAM on tumor cells. Girard *et al*.^40^ and Kurake *et al*.^33^, concluded that a synergistic effect between PAM-contained H_2_O_2_ and NO_2_^−^ is responsible for selective antitumor action of PAM. This view was substantiated by reconstitution experiments with defined concentrations of H_2_O_2_ and NO_2_^−^ ^59,60^. The interaction of these compounds was shown to result in the formation of primary ^1^O_2_ that caused local inactivation of membrane-associated catalase on tumor cells. This established autoamplificatory ^1^O_2_ generation by the tumor cells, catalase inactivation and reactivation of intercellular apoptosis-inducing RONS signaling. The apoptotic response required a preceding influx of H_2_O_2_ through aquaporins, which caused depletion of glutathione and sensitization of the cells for apoptosis induction through RONS signaling. This aquaporin-dependent step is analogous to the mechanism proposed by Yan *et al*.^57,58^, but required preceding inactivation of the gating catalase on the membrane. Freund *et al*.^61^ presented strong evidence that plasma-treated saline promotes an immunogenic phenotype of colon cancer cells, whereas Lin *et al*.^62^ concluded that PAM is not sufficient to induce immunogenic cell death, but that short-lived species in CAP are required. The discrepancy between these two studies might be explained by the low degree of induction of cell death by PAM in the study by Lin *et al*.^62^.

The preceding manuscript (Bauer *et al*.,^63^) has demonstrated that selective apoptosis induction in tumor cells by cold atmospheric plasma (CAP) generated by a portable air plasma ‘corona pen’ plasma source^64^ was essentially triggered by long-lived species from CAP-treated medium, i. e. by the components that are also found in plasma-activated medium (PAM). These are nitrite (NO_2_^−^) and H_2_O_2_, whose synergistic interaction is required for their antitumor action^33,40^. Our preceding study has shown that the direct effect of short-lived singlet oxygen (^1^O_2_) from the gaseous phase of CAP on treated cells seemed to be neglectable compared to the effects that can be attributed to PAM-related compounds. This finding can be explained by the preferential reaction (i.e. quenching) of ^1^O_2_ with medium components above or around the cells. These interactions prevent ^1^O_2_ derived from the gaseous phase of CAP from reaching its target, i. e. the tumor cells. In contrast, NO_2_^−^ and H_2_O_2_ are long-lived under the same conditions, but can lead to ^1^O_2_ generation in the vicinity of the target cells. Therefore, under our experimental conditions, the central effects of CAP and PAM treatment of tumor cells seem to be more or less the same, as they are both mainly based on long-lived species with the potential to generate ^1^O_2_ close to the target.

**Figure 1:**
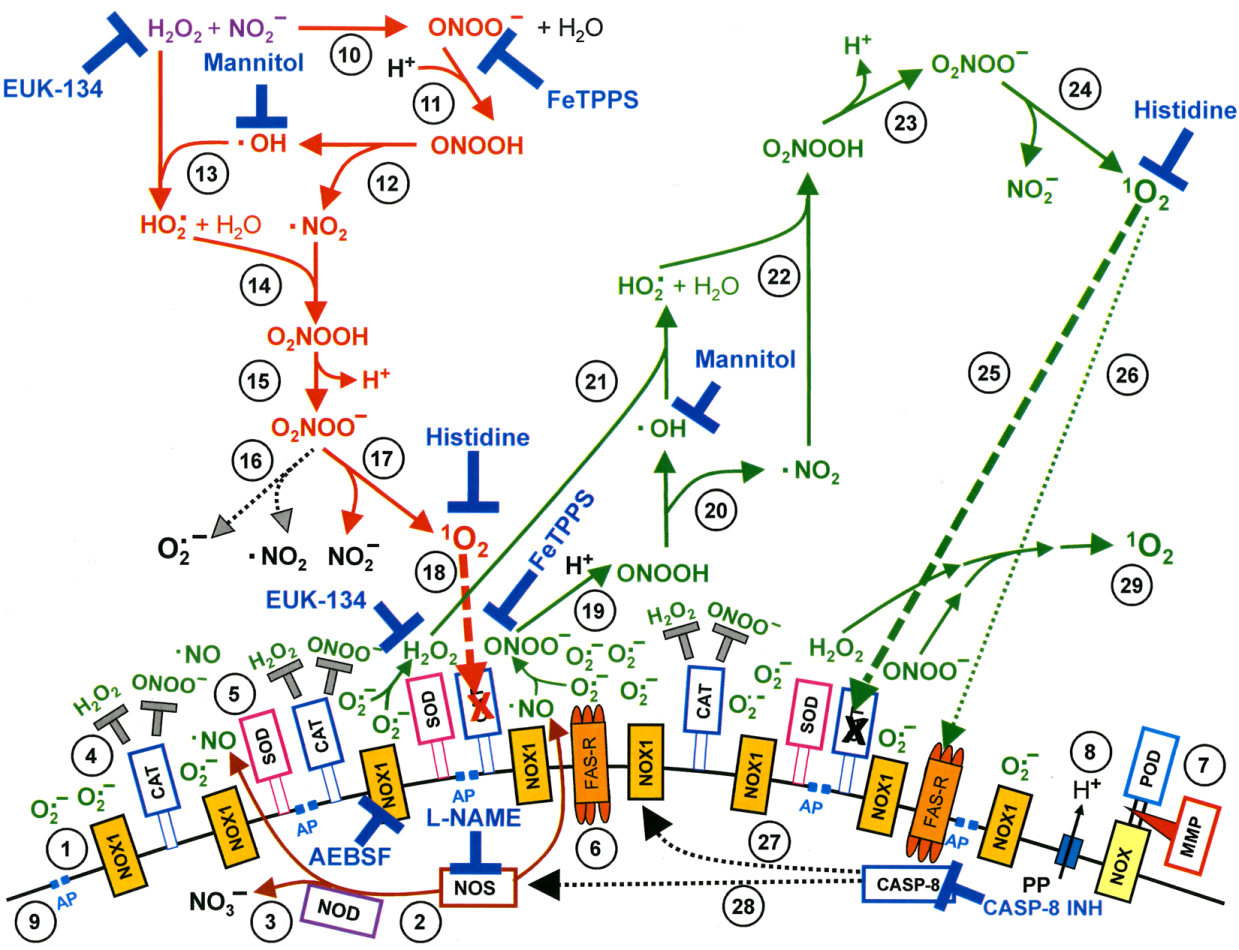
Apoptosis induction by CAP/PAM is mediated by the generation of primary and secondary singlet oxygen (^1^O_2_). NADPH oxidase 1 (NOX1) is expressed in the membrane of tumor cells and generates extracellular superoxide anions (O_2_^●−^) (#1). NO synthase (NOS) (#2) generates ^●^NO which can be either oxidated by ^●^NO dioxygenase (NOD) (#3) or pass through the cell membrane. Membrane-associated catalase (#4) protects tumor cells towards intercellular RONS-mediated signaling. Comodulatory SOD (#5) is required to prevent O_2_^●−^-mediated inhibition of catalase. Further important elements in the membrane are the FAS receptor (#6), Dual oxidase (DUOX) (#7), from which a peroxidase domain (POD) is split through matrix metalloprotease, proton pumps (#8) and aquaporins (#9). H_2_O_2_ and NO_2_^−^ derived from CAP treatment and stable in PAM interact and generate peroxynitrite (ONOO^−^) (#10). In the vicinity to membrane-associated proton pumps ONOO^−^ is protonated to peroxynitrous acid (ONOOH) (#11) and decomposes into ^●^NO_2_ and ^●^OH radicals (#12). ^●^OH radicals react with H_2_O_2_, resulting in the formation of hydroyperoxyl radicals (HO_2_^●^) (#13). The subsequent generation of peroxynitric acid (O_2_NOOH) (#14) and peroxynitrate (O_2_NOO^−^) (#15) allows for the generation of “primary singlet oxygen” (^1^O_2_) (#17). Primary ^1^O_2_ causes local inactivation of membrane-associated catalase (#18). Surviving H_2_O_2_ and ONOO^−^ at the site of inactivated catalase are the source for sustained generation of “secondary ^1^O_2_” through reactions #19- #24. Secondary ^1^O_2_ may either inactivate further catalase molecules (#25) and thus trigger autoamplification of ^1^O_2_ generation (#29), or activate the FAS receptor (#26) and in this way enhance the activities of NOX1 and NOS. This enhances the efficiency of secondary ^1^O_2_ generation. The site of action of specific inhibitors and scavengers are indicated. Please find details on the elements on the surface of tumor cells in references^74,80,97,118,124,125,130^, on singlet oxygen generation in references^59,96,97,118^, and on intercellular apoptosis-inducing signaling after catalase inactivation in references^78,80,83,132^.

The preceding manuscript, as well as reconstitution experiments with defined sources of H_2_O_2_ and NO_2_^−^ ^59,60^, presented evidence that the long-lived species H_2_O_2_ and NO_2_^−^ generated the “primary ^1^O_2_” through a complex cascade that started with the generation of peroxynitrite (ONOO^−^) after H_2_O_2_/NO_2_^−^ interaction^65,66^ (Fig. 1). This was followed by the reaction between ^●^OH  radicals, derived from peroxynitrous acid (ONOOH)^67^, with H_2_O_2_^68^. The resultant hydroperoxyl radicals (HO_2_^●^) then interacted with ^●^NO_2_ derived from ONOOH and thus enabled the formation of peroxynitric acid/peroxynitrate (O_2_NOOH/O_2_NOO^−^)^69^ as source for the generation of ^1^O_2_^69,70^. It was recognized that the generation of ONOOH from ONOO^−^ required the activity of membrane-associated proton pumps. This effect seemed to outcompete the consumption of ONOO^−^ through its favoured reaction with CO_2_^71–73^. In addition, protonation of ONOO^−^ through membrane-associated proton pumps directed the subsequent generation of ^1^O_2_ to the site where it is required, i. e. close to its target catalase.

The generation and action of primary ^1^O_2_ derived from the interaction between H_2_O_2_ and NO_2_^−^ in the presence of tumor cells seemed to be a relatively rare event^59,60^. This finding was not unexpected, as the generation of ^1^O_2_ in this biological system is limited by

- the decomposition of PAM-derived H_2_O_2_ by membrane-associated catalase of tumor cells,
- the relatively low reaction rate of ONOO^−^ generation through the interaction between NO_2_^−^ and H_2_O_2_^66^,
- the decomposition of ONOO^−^ generated by PAM through membrane-associated catalase of tumor cells^74,75^ and
- the reaction of ONOO^−^ with CO_2_ rather than with protons^71–73^, especially in locations realtively far from the proton pumps in the cell membranes.

Furthermore, the expected low concentration of primary ^1^O_2_ generated in the system only has a biological impact if it reaches its specific target on tumor cells, i. e. membrane-associated catalase, which is inactivated by ^1^O_2_^76,77^. This step is limited by the high reactivity of ^1^O_2_ and the resultant small free diffusion path length, due to ^1^O_2_ reaction with other competing substrates.

Therefore, primary ^1^O_2_ seems to have no chance to directly damage tumor cells to a degree that induces cell death^59,60^. However, primary ^1^O_2_ can utilize tumor cells like a switchboard, on which it triggers the sustained generation of “secondary ^1^O_2_” [*The term “secondary* ^1^*O*_2_*” is used in this paper for* ^1^O_2_ *that is generated by tumor cells after they have been triggered through local catalase inactivation by primary* ^1^O_2_]. This is achieved through the interaction between tumor cell-derived ONOO^−^ and H_2_O_2_. Both molecular species are constantly generated by tumor cells, due to their active NOX1 and NOS, and are no longer decomposed at sites of catalase that has been inactivated by primary ^1^O_2_ (Fig. 1). This biochemical scenario seems to result in a strong autoamplificatory process that drives catalase inactivation to a point at which intercellular apoptosis-inducing RONS signaling gets activated^60^. In parallel, substantial inactivation of membrane-associated catalase allows an influx of H_2_O_2_ into the cells^59^. This leads to the depletion of intracellular glutathione and thus sensitizes the cells for the apoptosis-inducing effect of lipid peroxidation. This step explains the dominant role of aquaporins for plasma-mediated apoptosis induction, as reported by Yan *et al*.^57,58^ though the time frame of action is different from the model proposed by these authors. Intercellular apoptosis induction was mainly based on the HOCl signaling pathway, which depends on H_2_O_2_-dependent HOCl synthesis by the peroxidase domain of DUOX, and subsequent HOCl/O_2_^●−^ interaction, leading to ^●^OH radicals that induce apoptosis through lipid peroxidation^78–80^.

**Figure 2:**
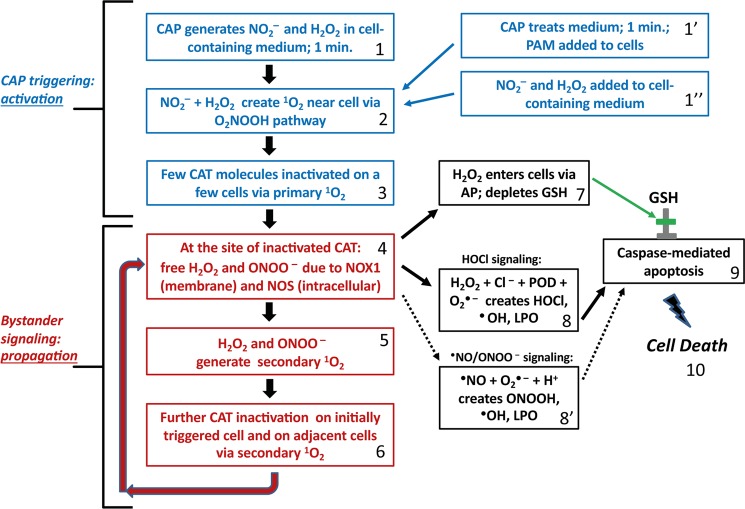
Flow chart of major steps in CAP leading to selective apoptosis of tumor cells. Step 1: CAP generates NO_2_^−^ and H_2_O_2_ in cell containing medium for 1 minute. Alternatively, CAP is used to treat medium, creating PAM (step 1′). Defined concentrations of NO_2_^−^ and H_2_O_2_ containing medium are used in reconstitution experiments (step 1”). Step 2: NO_2_^−^- and H_2_O_2_ create primary ^1^O_2_ near cells following O_2_NOOH pathway, as described in Figure 1. Step 3: Few catalase molecules on a few cells are inactivated due to primary ^1^O_2_ near cells. Step 4: At the site of inactivated catalase, H_2_O_2_ and ONOO^−^ (generated through NOX1 and NOS) are no longer decomposed. Step 5: The reaction between H_2_O_2_ and ONOO^−^ is leading ultimately to secondary ^1^O_2_. Step 6: This additional ^1^O_2_ leads to further catalase inactivation and the process cycles back to step 4. Step 7: Increased H_2_O_2_ resulting from catalase loss from secondary ^1^O_2_ leads to H_2_O_2_ entering cells via aquaporins, leading to antioxidant glutathione depletion. Step 8: In parallel with step 7, increased H_2_O_2_ resulting from catalase loss from secondary ^1^O_2_ also leads to HOCl generation by peroxidase, in the presence of Cl^−^. The interaction between NOX1-derived O_2_^●−^ leads to ^●^OH formation near the cell membrane and lipid oxidation. Step 8′: If HOCl signaling is suppressed, an alternative ^●^NO/ONOO^−^ signaling can also lead to lipid peroxdiation. Step 9: If both lipid peroxidation and glutathione depletion occurs, then caspase-associated apoptosis can take place, finally leading to cell death. Steps 1–3 correspond to CAP triggering or activation of a few cells, thereby initiating propagating bystander signaling in steps 4–6. Steps 7–9 are the steps that lead to the final cell apoptosis. These steps are activated only if the repeated performance of steps 4–6 has caused a sufficiently high degree of catalase inactivation for reactivation of HOCl or ^●^NO/ONOO^−^ - mediated apoptosis-inducing signaling.

In this way, even low concentrations of primary ^1^O_2_ trigger a massive, yet highly selective process directed towards tumor cells. The central overall process is summarized in the flowchart illustrated in Fig. 2, and further discussed in the light of this study and in relation to other models on CAP and PAM action, in Supplementary Figs 24 and 25. Nonmalignant cells remain unaffected by this process, as long as the H_2_O_2_ concentration in the system does not reach a level that leads to H_2_O_2_-mediated apoptosis induction in nonmalignant cells. Importantly, the apoptosis-inducing level of direct H_2_O_2_ application is lower for nonmalignant cells compared to tumor cells, due to the lack of membrane-associated catalase on nonmalignant cells^59,74,81^, (Bauer *et al*.,^63^. Therefore, selective apoptosis induction in tumor cells through the synergistic action of H_2_O_2_ and NO_2_^−^ requires that the H_2_O_2_ level is below the damaging level for nonmalignant cells.

Although Fig. 2 summarizes the key steps involved in selective tumor cell apoptosis following CAP treatment, it says nothing of the dynamics associated with each step. The present manuscript aims at the elucidation of the dynamics that drives the concerted action of primary and secondary ^1^O_2_ generation of CAP and PAM-treated tumor cells.

## Materials and Methods

### Materials

The NOX1 inhibitor 4-(2-Aminoethyl)benzenesulfonyl fluoride (AEBSF), the singlet oxygen (^1^O_2_) scavenger histidine, the NOS inhibitor N-omega-nitro-L-arginine methylester hydrochloride (L-NAME) and the HOCl scavenger taurine were obtained from Sigma-Aldrich (Schnelldorf, Germany).

The peroxynitrite decomposition catalyst 5-, 10-, 15-, 20-Tetrakis(4-sulfonatophenyl)porphyrinato iron(III) chloride (FeTPPS), was obtained from Calbiochem (Merck Biosciences GmbH, Schwalbach/Ts, Germany).

The catalase mimetic EUK-134 [chloro([2,2′-[1,2-ethanediylbis[(nitrilo-κN)methylidyne]]bis[6-methoxyphenolato-κO]]]-manganese was a product of Cayman (Ann Arbor, Michigan, U.S.A.) and was obtained from Biomol (Hamburg, Germany).

Detailed information on inhibitors has been previously published^74,82–86^. The site of action of inhibitors and scavengers has been presented in detail in the Supplementary Material of references #81 and #85.

### Cells and media for cell culture

The human gastric adenocarcinoma cell line MKN-45 (ACC 409) (established from the poorly differentiated adenocarcinoma of the stomach (medullary type) of a 62 year-old woman), was purchased from DSMZ, Braunschweig, Germany. MKN-45 were cultured in RPMI 1640 medium, containing 10% fetal bovine serum (FBS).

Fetal bovine serum (Biochrom, Berlin, Germany) was heated for 30 minutes at 56 °C prior to use. Medium was supplemented with penicillin (40 U/ml), streptomycin (50 µg/ml), neomycin (10 µg/ml), moronal (10 U/mll) and glutamine (280 µg/ml). Care was taken to avoid cell densities below 300 000/ml and above 10^6^/ml.

### Methods

#### The plasma sources

The portable air plasma ‘corona pen’ plasma source used here employs a neon-sign transformer with a rectifier and a high voltage multiplier and was developed in the framework of the frugal plasma biotech applications^64^. A high voltage needle electrode was inserted in a quartz tube. A DC-positive streamer corona discharge was generated on the needle electrode in ambient air, in a geometry similar to the discharge previously presented in^87,88^. The grounded electrode was a tin wire submerged in the cell culture medium at the bottom of the container. The distance of the needle tip to the medium surface was kept at 1 cm. The plasma discharge was directly hiting the liquid surface of the medium. The discharge voltage was kept at 10.7 kV and the maximum streamer pulse current was typically 17 mA with a repetitive frequency of 10 kHz. The streamer corona discharge generates RONS, such as O_3_, NOx, and ^●^OH radicals at very low deposited power (<0.1 W). All experiments described in this manuscript were performed with the streamer corona regime.

#### Treatment of cells with cold atmospheric plasma (CAP)

All treatments were performed in 24 well tissue culture clusters, 1 ml of medium and a grounded electrode. MKN-45 cells were used at a density of 125 000 cells/ml. The cells remain in suspension and only few cells attach firmly.

Standard treatment with CAP was by the streamer corona regime, with a distance of the plasma source from the top of the medium of 1 cm. Typical electrical parameters were voltage 10.7 kV, pulse amplitude 17 mA, and pulse frequency 10 kHz. The standard time of treatment was 1 min, unless otherwise indicated.

After treatment with CAP, the cells were either further incubated at 37 °C for the indicated times or subjected to washing steps and resuspension in fresh medium, depending on the protocol of the experiments. Whenever possible, culture was continued in 96 well plates with 100 µl medium after the washing step. These manipulations, which were essential for the analysis, are explained under “Strategy and design of our study”, as well as specified in the legends of the respective figures. The final step was always to determine the percentage of apoptotic cells induced by the treatment.

#### Generation and application of plasma-activated medium (PAM)

Complete medium without cells was treated with CAP for 1 min, unless otherwise specified. After 10 min, PAM was added to the cells that had been prepared at higher cell density, to reach a final concentration of PAM between 80–50%, as indicated. In some experiments, PAM was first serially diluted and then equal volumes of the dilution steps and cells of double standard density were mixed.

#### Determination of the percentage of apoptotic cells

After the indicated time of incubation at 37 °C and 5% CO_2_, the percentage of apoptotic cells was determined by inverted phase contrast microscopy based on the classical criteria for apoptosis, i.e., nuclear condensation/fragmentation or membrane blebbing^74,82,89,90^. The characteristic morphological features of intact and apoptotic cells, as determined by inverted phase contrast microscopy have been published^74,82,91–93^. At least 200 neighbouring cells from randomly selected areas were scored for the percentage of apoptotic cells at each point of measurement. Control assays ensured that the morphological features ‘nuclear condensation/fragmentation’ as determined by inverse phase contrast microscopy were correlated to intense staining with bisbenzimide and to DNA strand breaks, detectable by the TUNEL reaction^83,91–93^. A recent systematic comparison of methods for the quantitation of apoptotic cells has shown that there is a perfect coherence between the pattern of cells with condensed/fragmented nuclei (stained with bisbenzimide) and TUNEL-positive cells in assays with substantial apoptosis induction, whereas there was no significant nuclear condensation/fragmentation in control assays^82,93^. Further controls ensured that ROS-mediated apoptosis induction was mediated by the mitochondrial pathway of apoptosis, involving caspase-9 and caspase-3^93,94^.

#### Statistics

In all experiments, assays were performed in duplicate. Quantitative data are presented as means ± standard deviations. The statistical analysis comprised the comparison of groups such as assay without apoptosis induction/assay with apoptosis inducer or assay without inhibitor/assay with inhibitor. Therefore, the differences between two groups were analyzed by Student’s t-test (two-tailed), with N = 500 in all tests, and double checked with the Yates continuity corrected chi-square test. The confidence interval used was 95%. P < 0.01 was defined as “significant”; P < 0.001 as “highly significant”. The modules for the calculation of the tests were taken from https://www.quantitativeskills.com/sisa/statistics/t-test.htm (t test) and from http://www.quantpsy.org/chisq/chisq.htm (Chi-square test).

#### Strategy and design of our analysis

In the first part of this study, tumor cells were treated with CAP or PAM, and the resultant kinetics of apoptosis induction were used to define central molecular species and their interactions, that are involved in this process. This approach was instrumentalized for the comparison between the unrestricted response of tumor cells to CAP and PAM, and responses in which distinct signaling events had been blocked. For example, secondary ^1^O_2_ generation was selectively prevented either by inhibition of NOX1 through AEBSF or NOS through L-NAME. The generation of primary ^1^O_2_ from long-lived species was prevented by decomposing ONOO^−^ and H_2_O_2_ through FeTPPS and EUK-134, respectively. Alternatively, the focus was put on the potential role of ^1^O_2_ derived from the gaseous phase of CAP. This was achieved through initial treatment of cells under the conditions where ^1^O_2_ generation by long-lived CAP-derived species as well as the generation of secondary ^1^O_2_ were blocked. To achieve a final read-out of apoptosis induction in all of these treatments, the initial treatment with its specific restrictions to defined signaling events needed to be followed by conditions that allow resumption of secondary ^1^O_2_ generation and subsequent catalase inactivation and apoptosis-inducing RONS signaling. In total, this approach gave insight into the dynamics and connection of the processes in our biological system of CAP and PAM-treated tumor cells.

**Figure 3:**
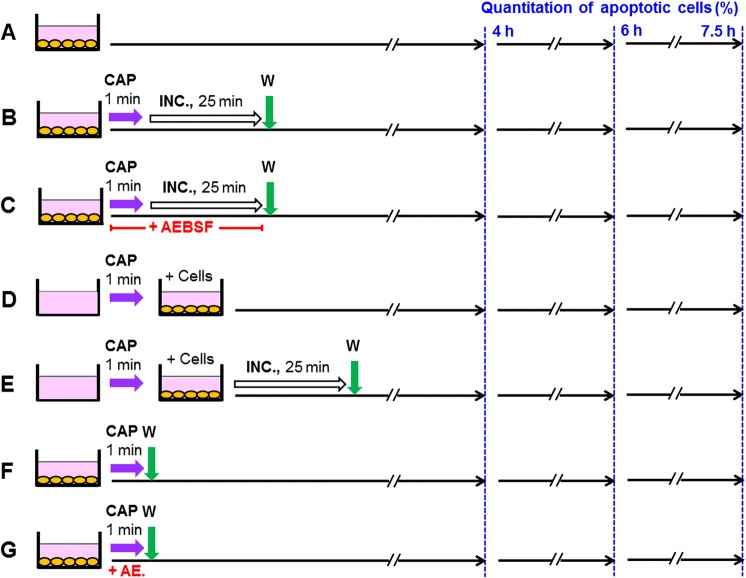
Scheme of the experimental procedures used in the experiment described in Fig. 8. (**A**) Untreated control cells (MKN-45, human gastric carcinoma cells) were cultivated in parallel to the other assays. (**B**,**C**) MKN-45 cells (125 000 cells/ml) in 24 well tissue culture clusters were treated with CAP for 1 min. After CAP treatment, the cells remained in contact with the same medium for 25 min. 100 µM of the NOX inhibitor AEBSF was present during CAP treatment and incubation in assay C. Assays were washed after the 25 min incubation step and were further cultivated in fresh medium. (**D**,**E**) Medium was treated with CAP for 1 min in the absence of cells and was then transferred to cells. The cells in assay (**E**) were washed after the incubation step and further cultivated in fresh medium. (**F**,**G**) Cells were treated with CAP for 1 min and then washed immediately. In assay (**G**), 100 µM AEBSF was present during CAP treatment. In all assays, the percentages of apoptotic cells were determined at 4, 6 and 7.5 h.

Figure 3 explains the procedures that were used in the experiment described later in Fig. 8. This introductory experiment already defined some of the key concepts and findings that were essential for our study. Figure 3 A describes the control without CAP treatment. Under 3B, cells in medium were treated with CAP for 1 min and the cells remained in contact with the treated medium for another 25 min (at 37 °C), before a washing step (three cycles) was applied and the cells received fresh medium. The assays described under 3C was analogous to 3B, except for the presence of the NOX inhibitor AEBSF during CAP treatment and subsequent incubation. This prevented the generation of secondary ^1^O_2_, but allowed the generation of primary ^1^O_2_ through the interaction between long-lived species derived from CAP. The washing step stopped the generation of primary ^1^O_2_, but allowed the resumption of secondary ^1^O_2_ generation, as AEBSF is a reversible inhibitor. Therefore, tumor cells that had got an “imprint” (i. e. inactivation of some catalase molecules on their outside) by primary ^1^O_2_ were now able to generate cell-derived, secondary ^1^O_2_ and thus to induce a bystander effect in neighbouring cells. This finally resulted in a kinetics of apoptosis induction that was effective, but delayed compared to the assays without interference with signaling, such as in 3B. In the assays under 3D, E the medium was treated with CAP in the absence of cells. Cells were added to the treated medium afterwards. Under 3E, the contact between CAP-treated medium and cells was restricted to 25 min through a washing step. In the assays described by 3F, G, the cells were treated with CAP (in the absence or presence of AEBSF) and the treated medium was immediately removed after CAP treatment. This regime prevented the contact between the cells and primary ^1^O_2_ from long-lived species in the treated medium. Under G, also the generation of secondary ^1^O_2_ during the time of CAP treatment was prevented through AEBSF. 3F and G therefore allowed to focus on the imprint by primary ^1^O_2_ derived from the gaseous phase of CAP. In all assays, the percentage of apoptotic cells was determined at three different time points.

**Figure 4:**
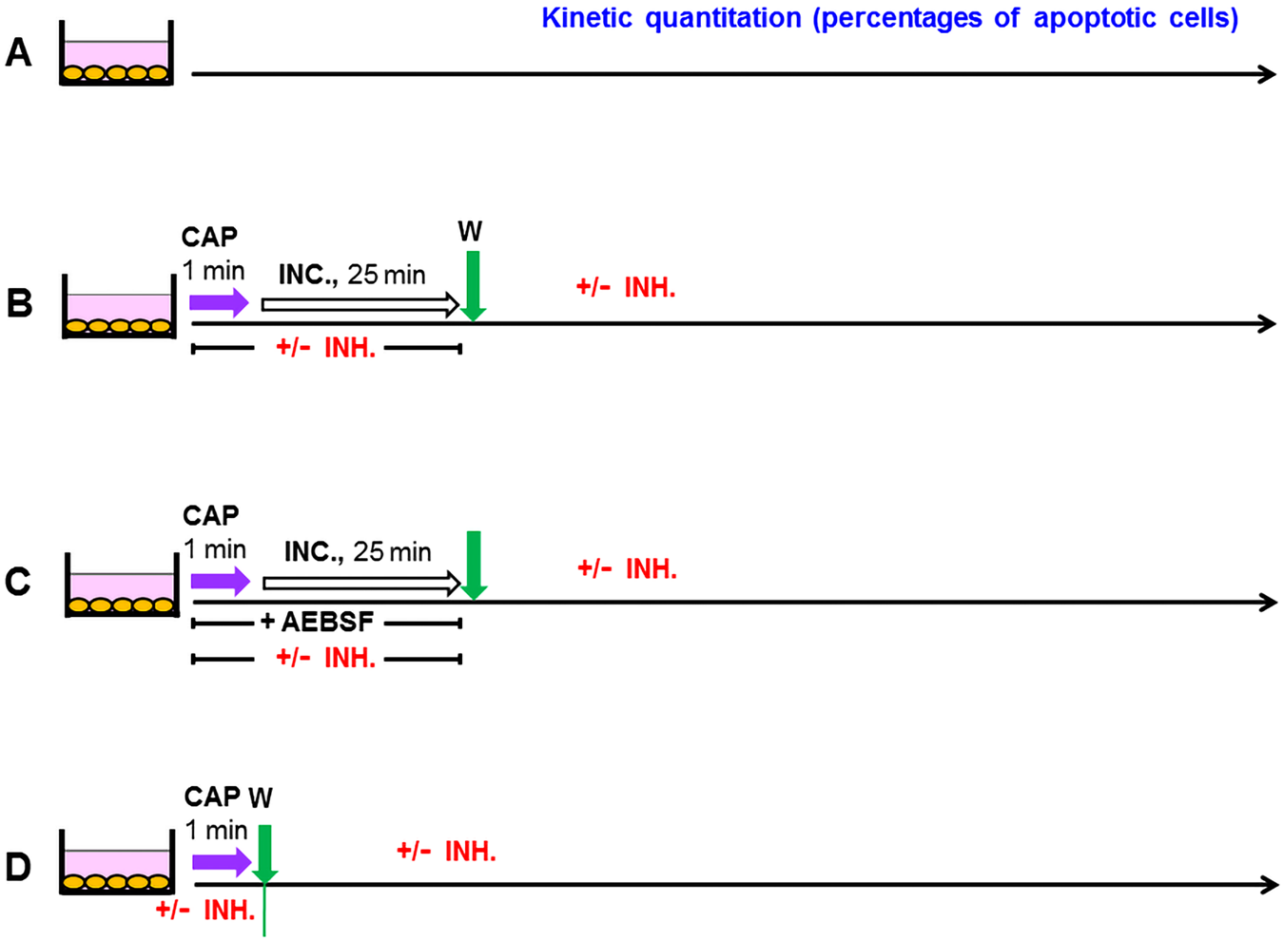
Basic scheme of the experiments described in Figs 9–13. (**A**) Untreated MKN-45 cells (control). (**B,C**) MKN-45 cells were treated with CAP for 1 min. The cells were further incubated in the same medium for additional 25 minutes and then washed (3 cycles) and resuspended in fresh medium. Inhibitors (INH) (as indicated in the respective figures) were present during CAP treatment and the incubation step. In the assays described under C, 100 µM AEBSF was present in all assays (for prevention of the generation of secondary ^1^O_2_), together without or with additional inhibitors, as indicated. Inhibitors were also added after the washing step, where indicated. (**D**) Cells were treated with CAP for 1 min, in the absence or presence of the indicated inhibitors and were then washed immediately and resuspended in fresh medium. Where indicated, inhibitors were added and cultivation was continued. The percentages of apoptotic cells were determined kinetically.

The subsequent kinetic analysis, as described in Figs 9–12 all followed a protocol that is illustrated in Fig. 4. Treatment with CAP was for 1 min and was followed by an incubation for 25 min in the treated medium, before a washing step was performed (4B,C) or alternatively, the washing step was performed immediately after the CAP treatment (4D). Assays according to 4 C always contained AEBSF during CAP treatment and the subsequent incubation. Further inhibitors or scavengers, as specified in the respective figures were added either during treatment before the washing step or thereafter. The quantitation of the percentages of apoptotic cells was performed kinetically. The interpretation of the data requires to recapitulate that (i) histidine scavenges primary ^1^O_2_ as well as secondary ^1^O_2_, (ii) FeTPPS decomposes ONOO^−^ that is required for the generation of primary ^1^O_2_ through the interaction between H_2_O_2_ and NO_2_^−^ as well as for the generation of secondary ^1^O_2_ and (iii) that AEBSF inhibits NOX1 and thus prevents the generation of cell-derived H_2_O_2_ and peroxynitrite that are required for the generation of secondary ^1^O_2_.

The analysis shown in Fig. 13 used the same principle approach as described in Fig. 9, except that medium was treated with CAP in the absence of cells. The resultant plasma-activated medium (PAM) was then brought into contact to cells.

**Figure 5:**
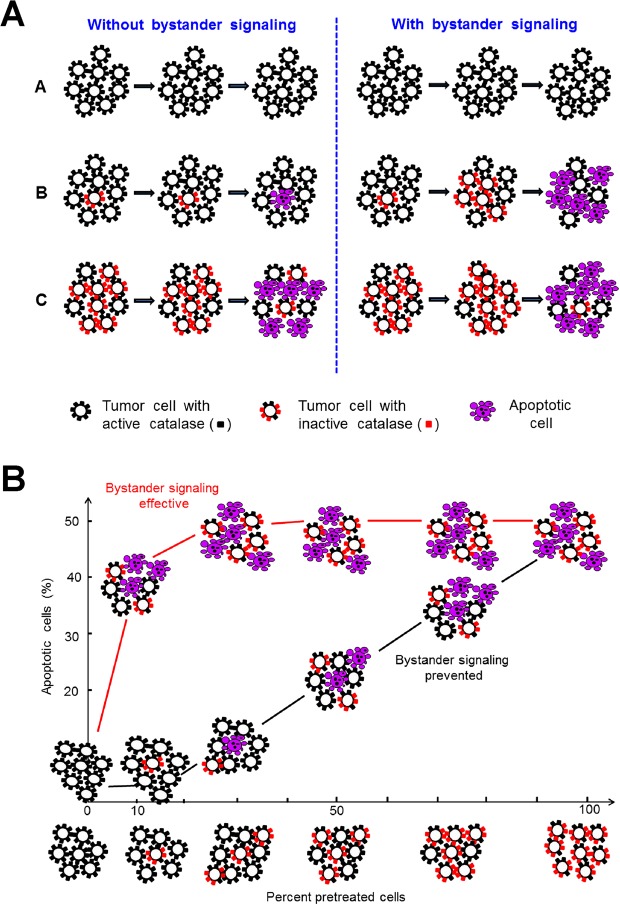
The principle of bystander signaling between tumor cells with inactivated catalase and untreated tumor cells. (**A**) The principle. Left side: In a mixture between tumor cells with inactivated membrane-associated catalase and untreated tumor cells the final percentage of apoptotic cells would be strictly correlated to the percentage of pretreated cells in the population. Right side: Experimental evidence has been provided^60,95,96^ that tumor cells with inactivated catalase drive a singlet oxygen-mediated bystander signaling that causes inactivation of membrane-associated catalase, reactivation of intercellular RONS signaling and apoptosis induction. As a result, the percentages of apoptotic cells are much higher than to be expected from the percentage of pretreated cells in the mixedpopulation. (**B**) Quantitative analysis of bystander signaling. The percentage of apoptotic cells is much higher than the percentage of pretreated cells with inactivated catalase, when bystander signaling is effective. Inhibition of bystander signaling leads to percentages of apoptotic cells that strictly correlate with the percentages of pretreated cells. This system allows analysis of the underlying molecular mechanisms through application of defined inhibitors or scavengers at distince steps. It also allows to determine the percentage of bystander-inducing cells in a population through titration of cells from this population on untreated cells and determining the percentage of apoptotic cells. If the induction of bystander inducing potential is a rare effect, a large portion of a cell population will be needed to induce bystander signaling, whereas a population with a high percentage of bystander effect-inducing cells will allow bystander induction even with a low percentage of these cells within an untreated population.

**Figure 6:**
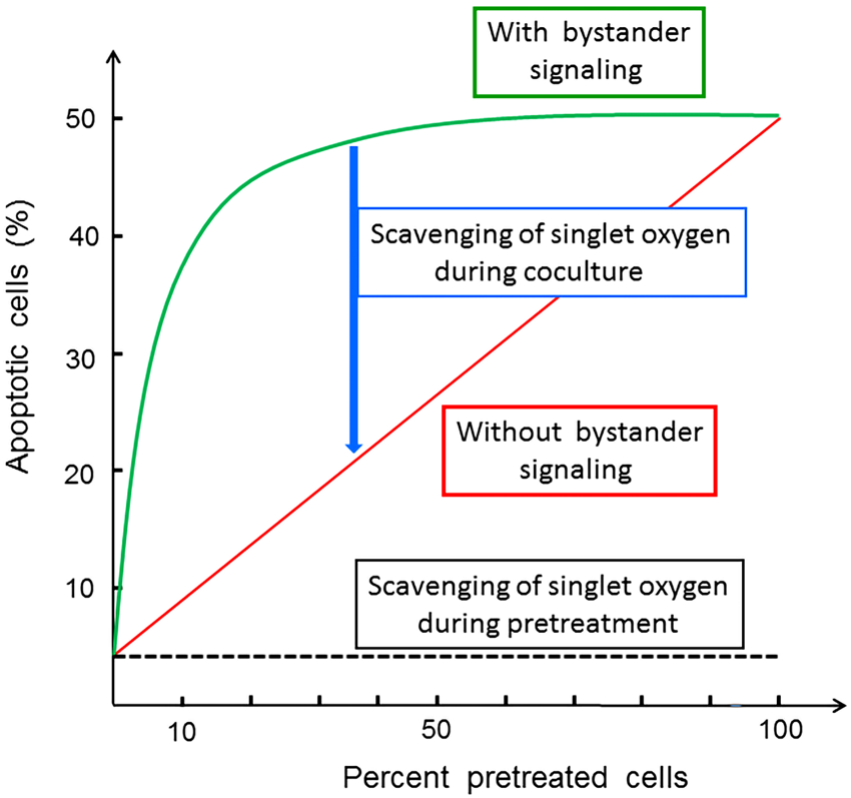
Interpretation of the results from bystander experiments. If a tested population of pretreated cells shows apoptotic potential, but no bystander effect induction, the dependency of apoptosis induction on the percentage of pretreated cells is a strict line. In the case of bystander signaling, the resultant curve is hyperbolic. It can be reduced to the linear relationship when inhibitors of bystander signaling (such as the singlet oxygen scavenger histidine or other relevant inhibitors) are present during the coculture phase. If pretreatment of tumor cells is a singlet oxygen-dependent pathway as well (as in the case of CAP and PAM), the presence of certain inhibitors will neither allow apoptosis induction nor bystander induction. If the number of bystander inducing cells in the pretreated population is low, the hyperbolic curve will be shifted more to the right, thus allowing quantitation of the bystander-inducing population.

In the second part of the manuscript, tumor cells were pretreated with CAP or PAM under defined analytical conditions and were then added at increasing percentages to untreated tumor cells. This approach was based on a proof of concept experiment for “bystander effect inducing potential” of tumor cells with inactivated catalase^60,95,96^. The principle of this test system is shown in Figs 5 and 6.

**Figure 7:**
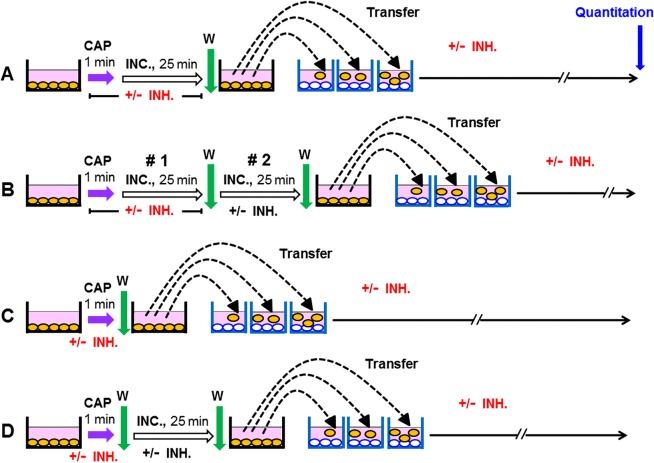
Basic scheme of the bystander experiments performed in this study (Figs 14–19). Tumor cells were treated with CAP for 1 min and then either further cultivated in the same medium for additional 25 min, and then washed (**A,B**), or washed immediately after CAP treatment (**C,D**). Pretreated cells from assays A and C were added at increasing percentages to untreated tumor cells immediately after the washing step. Pretreated cells from assays B and D  were subjected to 25 min incubation after the first washing step, were then washed and added to untreated tumor cells at increasing percentages. As indicated in this figure and specified in the respective legends, defined inhibitors or scavengers could be applied at various steps. The titration of the pretreated cells, in combination with their potential for bystander signaling, allows to quantitatively determine the number of cells that had obtained an “imprint” during a specific, experimentally defined step. This allows to conclude back on the dynamics of the underlying processes. The use of inhibitors and scavengers thereby allows to define the chemical biology involved in these dynamic interactions.

Combined with differential treatment and use of inhibitors/scavengers at defined steps, this approach allowed at least a semiquantitative estimation of the contribution of the individual signaling elements to the overall process and its underlying dynamics. The experimental procedures for the study of bystander signaling of tumor cells after treatment with CAP or PAM are summarized in Fig. 7.

The variability of the mode and time of CAP treatment, the placement of the washing steps, the length of the incubation steps and of the inhibitors/scavengers opens the chance to selectively pinpoint all intermediate steps and to obtain quantitative information on them.

The interpretation of the data obtained in this study was also based on and counter-controlled by results that had been obtained in reconstitution experiments, in which defined long-lived species from CAP and PAM, i. e. H_2_O_2_ and NO_2_^−^ had been tested for their effects on tumor cells and nonmalignant cells^59,60^. Furthermore, experiments with the same plasma source as used in this study had allowed to define the essential steps in tumor cell/plasma interaction, such as generation of primary and secondary ^1^O_2_, catalase inactivation, glutathione depletion after H_2_O_2_ flux through aquaporins, intercellular apoptosis-inducing RONS signaling and activation of the mitochondrial pathway of apoptosis^63^.

## Results

### Dissection of the experimental system and kinetic analysis

**Figure 8:**
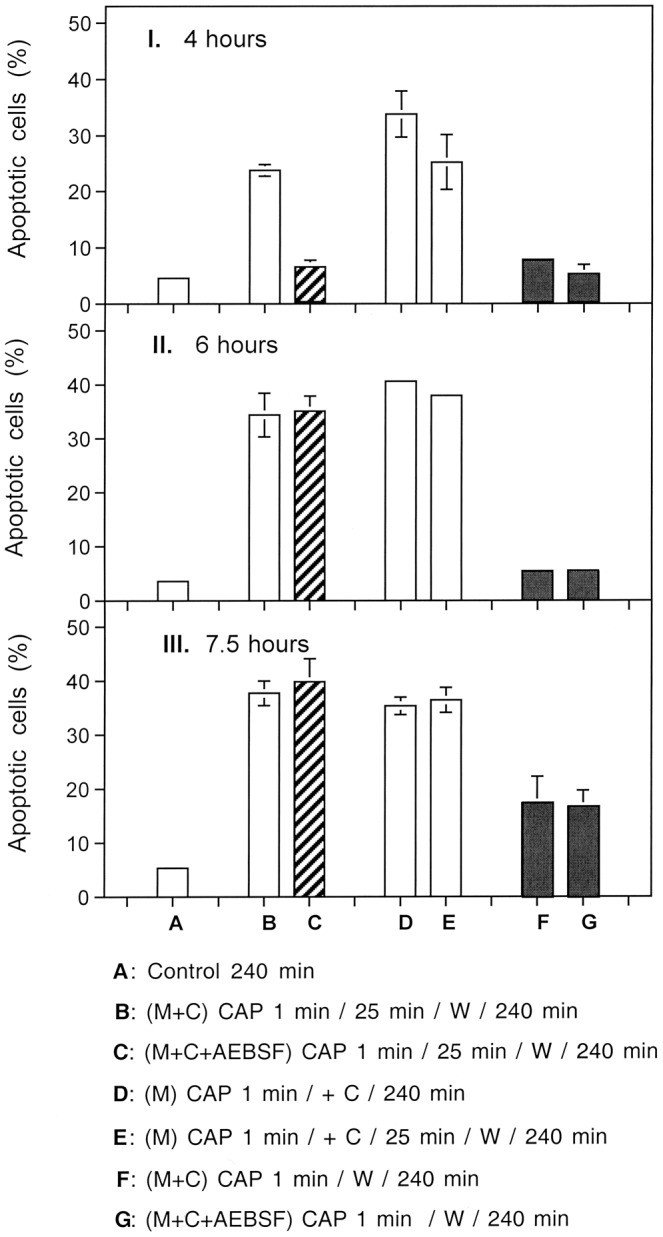
Kinetic and mechanistic aspects of different modes of CAP treatment of tumor cells. I. (**A**) MKN-45 cells remained untreated and were incubated for 4 h. (**B**) Medium and cells were treated with CAP for 1 min, followed by 25 min of incubation, a washing step and further incubation in fresh medium up to 4 h; (**C**) The NOX1 inhibitor AEBSF (100 µM) was present during the preatreatment of cells + medium with CAP for 1 min, followed by 25 min incubation, a washing step and further incubation in fresh medium. (**D**) Medium was treated with CAP for 1 min and then tumor cells were added to reach a final concentration of PAM of 80%. The assays were further incubated up to 4 h. (**E**) Medium was treated CAP for 1 min and then tumor cells were added. The assays were further incubated for 25 min, washed, and further incubated in fresh medium up to 4 h; (**F**) Medium plus cells were treated with CAP for 1 min and then washed immediately, resuspended in fresh medium and further cultivated. (**G**) The assay was performed as described undeer F, with the modification that AEBSF was present during CAP treatment. II., III.: The conditions and assays are identical to those described under I, but the time of assessment of apoptosis was at 6 h or 7.5 h after CAP treatment, respectively. The results show that CAP treatment of tumor cells in medium, followed by 25 min incubation in the same medium causes apoptosis induction to the same degree as treatment of medium with CAP, followed by incubation of cells in this plasma-activated medium (PAM). This points to the dominant action of long-lived species for apoptosis induction. The presence of AEBSF during CAP treatment and 25 min incubation causes a kinetic delay, which is explained by initial prevention of secondary singlet oxygen (^1^O_2_) generation, followed by resumption of secondary ^1^O_2_ generation after the washing step and incubation in fresh medium. Separation of the CAP-treated cells from their medium immediately after 1 min CAP treatment results in a very long delay in apoptosis induction, as ^1^O_2_ from the gaseous phase of CAP is the only initial trigger under the conditions in assays F and G.

Monitoring of apoptosis induction 4 h after treatment of tumor cells with CAP or PAM (Fig. 8 I) showed that direct treatment of tumor cells with CAP for 1 min, followed by 25 minutes of *incubation in the same medium*, and a subsequent washing step (condition B), caused a similar apoptosis-inducing effect as addition of PAM to untreated tumor cells (condition D,E). When the NOX inhibitor AEBSF had been present during CAP treatment plus the 25 min incubation step (condition C), no significant apoptosis induction was observed. Separation of the cells from the surrounding medium immediately after CAP treatment (condition F, G), also did not lead to detectable apoptosis induction within 4 h.

When the quantitation of apoptotic cells was performed two hours later (Fig. 8 II), the inhibitory effect through the presence of the NOX inhibitor during CAP treatment and 25 min incubation phase (C) was no longer observable, whereas cells treated according to regime F and G still showed no apoptosis induction. At six hours, the assays that had already been positive at four hours seemed to have reached their plateau phase of apoptosis induction, as can be concluded from comparing Fig. 8 I with Fig. 8 II. However, Fig. 8 III (measurement at 7.5 hours) indicated that conditions F and G seemed to allow late occurence of apoptosis induction. These data point to a complex system of multiple CAP and PAM interactions with the tumor cells. These interactions seemed to occur in parallel, but with different requirements, kinetics and efficiencies. The data also show that long-lived species in plasma-activated medium trigger the major effect achieved with CAP treatment. The effect of the NOX inhibitor AEBSF points to the role of tumor cell-specific NOX1 in this process, conceivably through generation of secondary ^1^O_2_.

**Figure 9:**
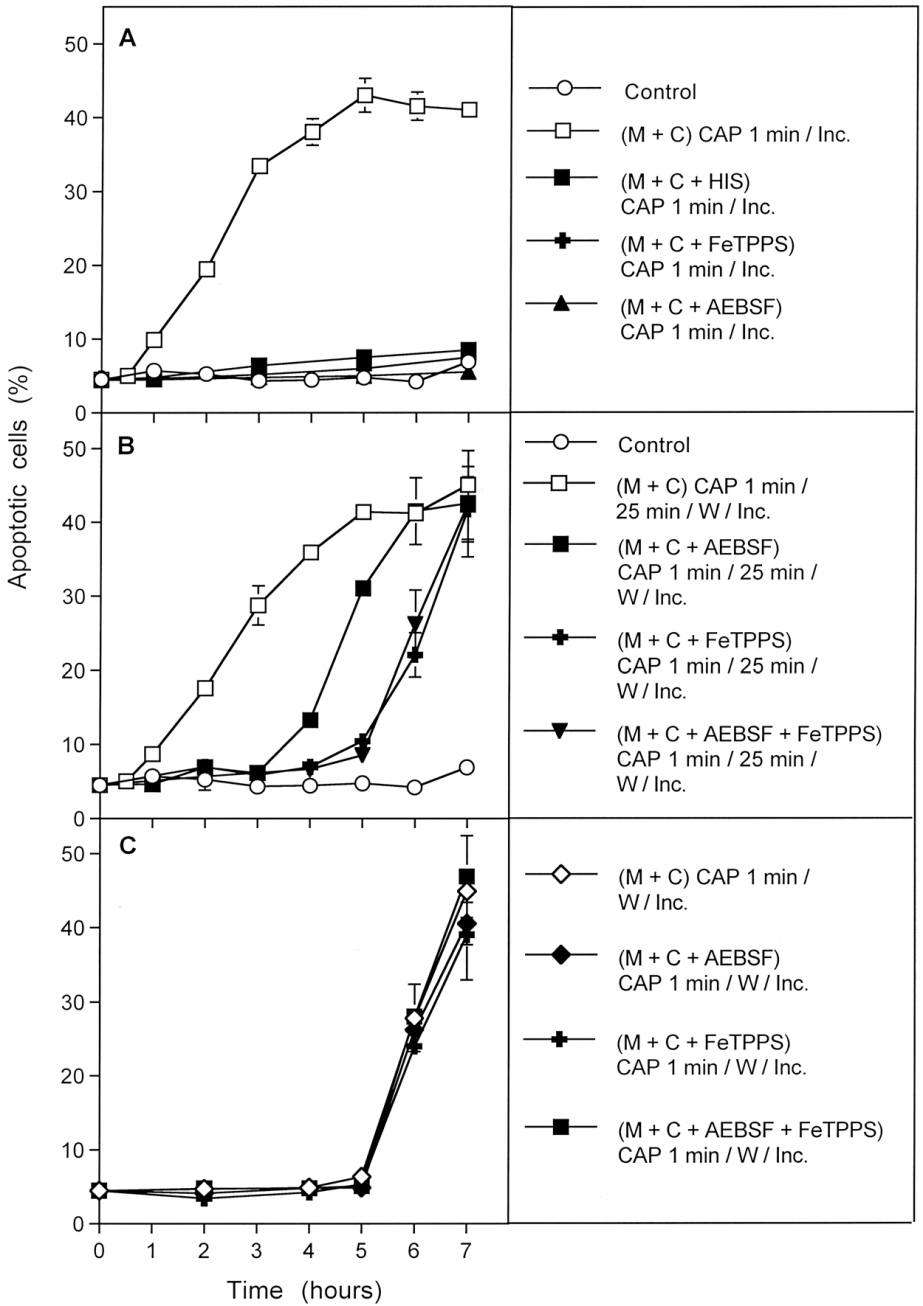
Dissection of CAP-mediated apoptosis into three kinetically defined processes. (**A**) MKN-45 cells in medium (“M + C”) were not pretreated (control) or treated with CAP for 1 min in the absence or presence of either 2 mM of the singlet oxygen (^1^O_2_) scavenger histidine, 25 µM of the peroxynitrite (ONOO^−^) decomposition catalyst FeTPPS or 100 µM of the NOX1 inhibitor AEBSF. The assays were incubated after CAP treatment for the indicated times (without any washing steps). (**B**) MKN-45 cells were not pretreated or treated for 1 min with CAP in the absence or presence of the indicated inhibitors. The assays were further incubated for 25 min and then subjected to three cycles of washing. The cells were resuspended in fresh medium and further incubated, as indicated. (**C**) The tumor cells were treated with CAP for 1 min in the absence or presence of the indicated inhibitors. Immediately after CAP treatment, the cells were subjected to three cycles of washing, resuspended in fresh medium and cultivated further, as indicated. In all assays, time zero in is the beginning of CAP treatment. The data show that experimental modifications after CAP treatment allow to define three kinetically different processes.

The next experiment allowed to differentiate between three kinetically distinct processes after CAP action. As shown in Fig. 9A, the overall process of apoptosis induction in CAP-treated tumor cells was completely inhibited when either scavenging of ^1^O_2_ by histidine, decomposition of ONOO^−^ by FeTPPS, or inhibition of NOX1 by AEBSF continued over the whole time of the experiment. This points to the dominating role of ^1^O_2_, ONOO^−^ and superoxide anions (O_2_^●−^) for this process, but does not yet allow to differentiate between the different phases of the process or between potentially existing multiple pathways.

However, the use of specific inhibitors during the initial phase of CAP treatment and 25 min incubation before the washing step allowed a clear differentiation between three kinetically different processes (Fig. 9B). In the absence of inhibitors, an overall process (#1) started after a lag phase of 30 minutes and reached a plateau at about 5 hours of incubation. When NOX1 had been inhibited by AEBSF, and thus autoamplification of ^1^O_2_ generation had been prevented during the first 25 minutes of the experiment, the kinetics of apoptosis induction in this process (#2) was delayed for about 3 hours. It then approached the efficiency of the overall process. Process #2 seemed to depend on ONOO^−^, as it was inhibited by FeTPPS. The curve obtained for process #2 inhibited by FeTPPS was indistinguishable from the kinetics of a further process (#3).

This kinetically different process (#3) started five hours after CAP treatment. It also was independent of NOX1 activity during CAP treatment and the first 25 minutes of incubation, but in addition did not require the presence of ONOO^−^ during this initial phase. Importantly, this third process was also demonstrated in full activity and unchanged kinetics in cells that had been removed from their surrounding medium immediately after CAP treatment (Fig. 9C), and therefore were not under the influence of the long-lived species in the medium for longer time. In contrast, process #1 and #2 were not observed in a cell population that had been separated from the medium containing long-lived CAP-derived species, immediately after the 1 min treatment with CAP. Process #3 was not inhibited by the presence of AEBSF or FeTPPS alone or in combination during CAP treatment, provided CAP treatment was followed by a washing step to remove the inhibitors.

**Figure 10:**
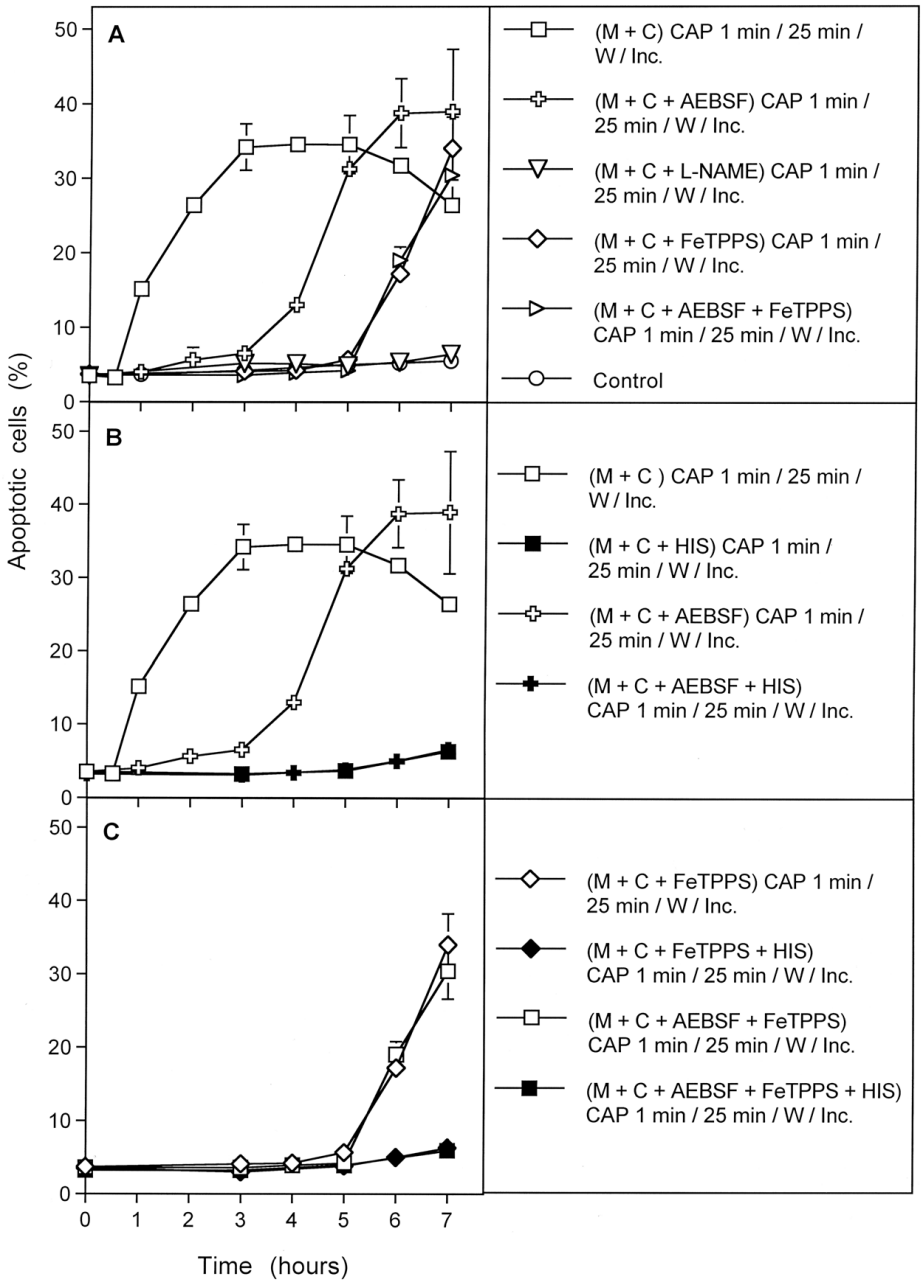
The CAP-induced processes are dependent on the action of singlet oxygen (^1^O_2_). MKN-45 cells in medium were treated for 1 min with CAP in the absence of inhibitors or in the presence of the indicated inhibitors. All assays were further incubated after CAP treatment for 25 min, before they were subjected to three cycles of washing. Further incubation was performed in fresh medium without any inhibitors and the percentages of apoptotic cells were monitored kinetically. The results show three kinetically different processes which all were completely blocked when the ^1^O_2_ scavenger histidine (HIS) was present during CAP treatment and the 25 min incubation step following CAP treatment. Assays containing L-NAME showed no apoptosis induction, as L-NAME is an irreversible inhibitor of NOS. The inhibitors were applied at the following concentrations: HIS (2 mM); AEBSF (100 µM), FeTPPS (25 µM), L-NAME (2.4 mM).

Processes #1–#3 were completely prevented when the ^1^O_2_ scavenger histidine had been present during CAP treatment and the subsequent 25 min incubation step (Fig. 10). This demonstrates the central function of ^1^O_2_ for each single one of these processes.

**Figure 11:**
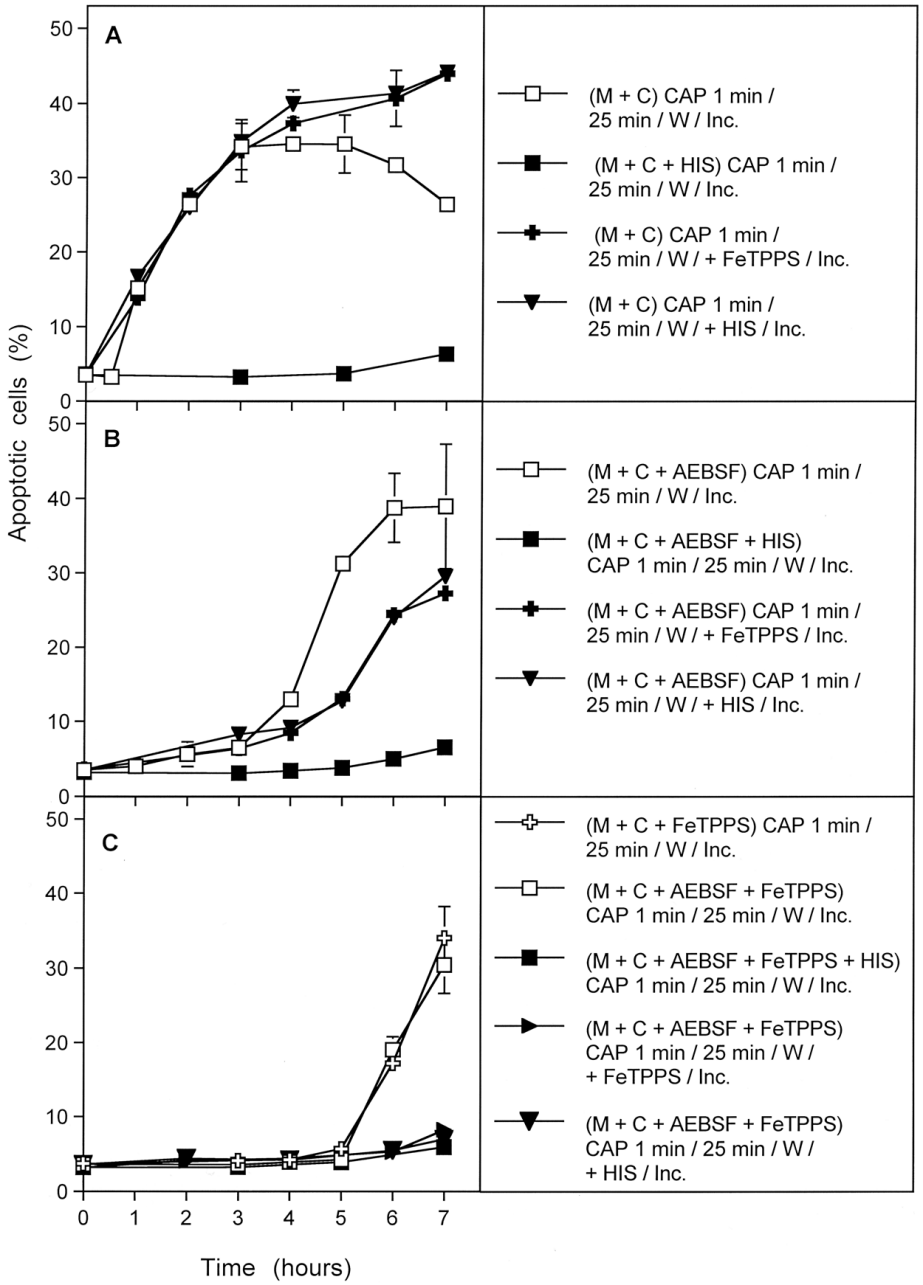
The kinetically determined, CAP-induced processes show differential response to early and late addition of inhibitors. The experiments were performed in analogy to those described in Fig. 10. In addition, parallel assays also received either 2 mM of the singlet oxygen (^1^O_2_) scavenger histidine or 25 µM of the peroxynitrite (ONOO^−^) decomposition catalyst FeTPPS after the washing step that followed CAP treatment and the first incubation step of 25 min. Assays without CAP treatment showed less than 5% apoptotic cells at all time points (not shown in the graph). (**A**) Process #1 was inhibited when histidine was present during CAP treatment and the first 25 min incubation step, but not when histidine had been added after the washing step. Likewise, FeTPPS added after the washing step had no inhibitory effect. (Note that addition of FeTPPS during CAP treatment would have shifted process #1 to process #3, shown below). *Process #1 is the overall process, based on unlimited primary and secondary singlet oxygen generation*. (**B**) Process #2 was induced when AEBSF was present during CAP treatment and the 25 min incubation step. Process #2 was completely inhibited by the presence of histidine during CAP treatment and the 25 min incubation step, and partially inhibited when histidine or FeTPPS had been added after the washing step. *Process #2 reflects the regime in which secondary* ^1^O_2_ *generation is blocked during CAP treatment and the subsequent 25 min incubation step. After the washing step, secondary* ^1^O_2_ *generation resumes, driven by the imprinted signature established initially by primary* ^1^O_2_*generated by long-lived species of CAP-treated medium*. (**C**) Process #3 is independent of ONOO^−^ and NOX derived O_2_^●−^ during CAP treatment and the 25 min incubation step, but completely dependent on ^1^O_2_ at this step. The process after the washing step is completely dependent on ^1^O_2_ and on ONOO^−^. *Process #3 depends on the imprinted signature by primary* ^1^O_2_ *from the gaseous phase of CAP. The imprinted signature drives secondary* ^1^O_2_ *generation after the washing step*.

Addition of the ^1^O_2_ scavenger histidine or the ONOO^−^ decomposition catalyst FeTPPS *after* the 25 min incubation step had a differential impact on processes #1–#3 (Fig. 11). The overall process (#1) was no longer inhibited by histidine or FeTPPS under this condition of late addition of inhibitor (Fig. 11A) indicating that an early singlet oxygen- and ONOO^−^-dependent process was completed at the time of addition of inhibitors. Process #2 was still partially inhibited by late addition of histidine and ONOO^−^ (Fig. 11B), indicating that a ^1^O_2_- and ONOO^−^-dependent step was only partially completed at the time of addition of inhibitors. Importantly, process #3 was completely blocked by the late addition of histidine and FeTPPS (Fig. 11C). This indicates a remarkable switch of process #3 from an initial independence of ONOO^−^ to the dependence on ONOO^−^ later on, and the requirement for ^1^O_2_ during this late phase. In other words, process #3 seemed to be triggered by ^1^O_2_ that was not generated by a process that required ONOO^−^ as intermediate, whereas the propagation of its apoptosis-inducing effects required ^1^O_2_ generation with ONOO^−^ as an intermediate later on.

**Figure 12:**
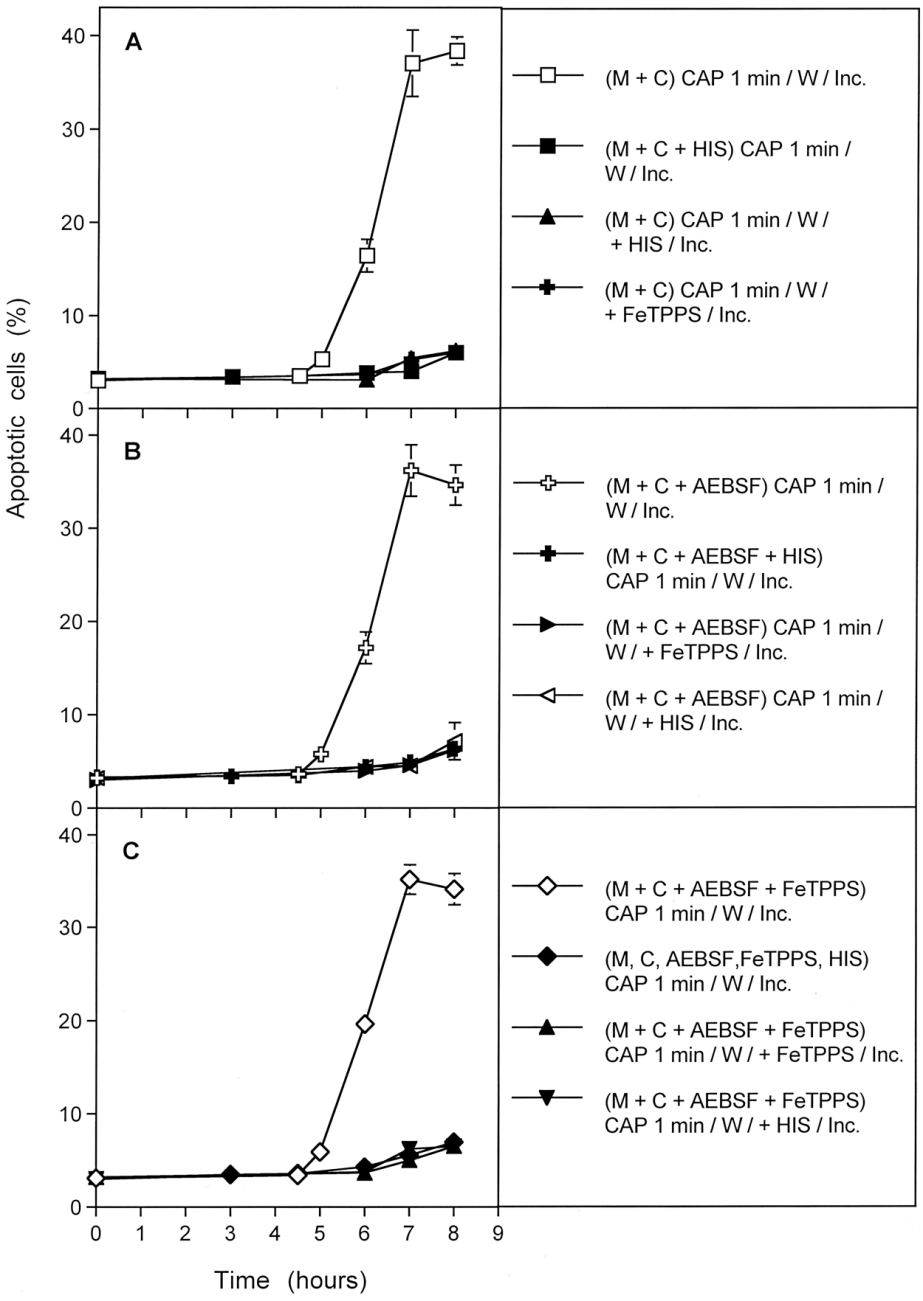
Further details of process #3. Process #3 was established either by treating tumor cells with CAP for 1 min, followed by an immediate washing step and further incubation in fresh medium. During CAP treatment, the tumor cells were not confronted with inhibitors (**A**), or with 100 µM of the NOX1 inhibitor AEBSF (**B**) or AEBSF plus 25 µM of the ONOO^−^ decomposition catalyst FeTPPS (**C**). As indicated, assays contained additional histidine or FeTPPS, either during CAP treatment, or during the incubation phase following the washing step. Control assays without CAP treatment did not show apoptosis induction above 4% (data not shown). The results show that process #3 is initiated by primary singlet oxygen from the gaseous phase of CAP, independent of H_2_O_2_/NO_2_^−^-dependent primary ^1^O_2_ (as the effect was not inhibited by FeTPPS during treatment) and independent of secondary ^1^O_2_, as it was not inhibited by AEBSF. However, the imprinted signature induced by primary ^1^O_2_ from the gaseous phase of CAP allowed the generation of secondary ^1^O_2_ after washing, as seen by the inhibitory effects of histidine and FeTPPS.

When process #3 was induced specifically in cells that had been removed from their surrounding medium immediately after CAP treatment (in the absence or presence of AEBSF or FeTPPS or both of them), the dependency of this step on ^1^O_2_ as well as ONOO^−^ at the time following the washing step was clearly shown. Furthermore the dependence on ^1^O_2_, but independence on ONOO^−^ of this step during CAP treatment was confirmed (Fig. 12).

**Figure 13:**
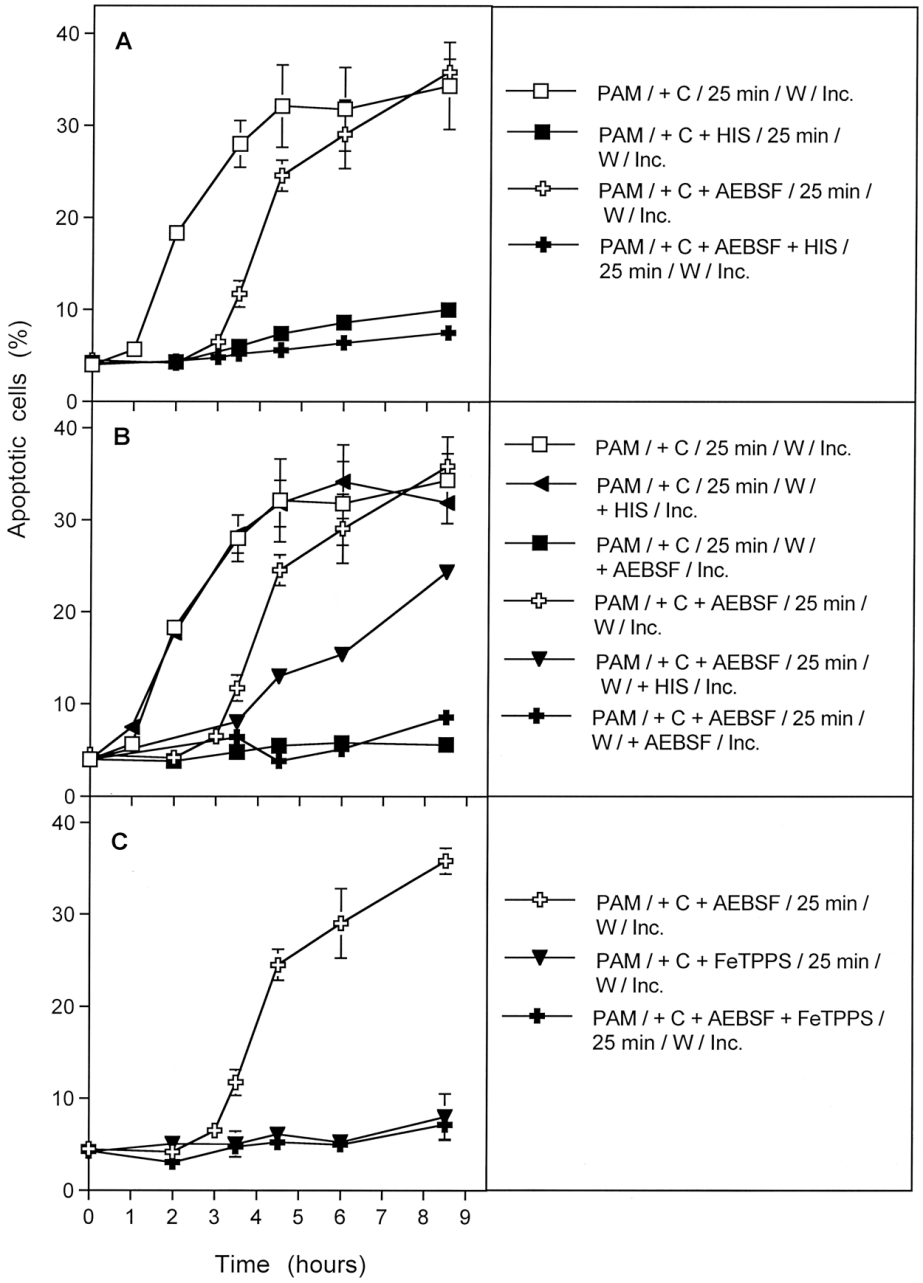
Plasma activated medium (PAM) triggers processes #1 and #2, but not #3. Medium was treated with CAP for 1 min and then an equal volume of cells of double standard density was added. Where indicated, inhibitors were added during the initial contact of PAM and cells. After 25 min of incubation, assays were subjected to three cycles of washing and the cells were resuspended in fresh medium. Where indicated, inhibitors were added at this step. Inhibitors were added at the following concentrations: AEBSF (100 µM); FeTPPS (25 µM), HIS (2 mM). The assays were further incubated and the percentages of apoptotic cells were determined kinetically. Time zero in all assays is the time of mixing PAM and cells. Control assays without PAM did not show apoptosis induction above 4% at all time points (data not shown). The results show that PAM induces the kinetic processes #1 and #2 with equal efficiency and characteristics as shown for direct CAP treatment in the preceding figures, whereas PAM has no potential to initiate process #3. This finding is in line with the short-lived nature of singlet oxygen derived from the gaseous phase of CAP that is responsible for process #3. These findings demonstrate the generation of primary ^1^O_2_ through the interaction of the long-lived species in PAM and to the role of secondary ^1^O_2_ that is generated by the tumor cells, dependent on their active NOX1.

A kinetic analysis of PAM-treated tumor cells showed that PAM induced process #1 and #2 with equal efficiency and with identical kinetics and inhibition profiles as shown for direct CAP treatment (Fig. 13A,B). However, process #3 with its initial independence of both O_2_^●−^ and ONOO^−^, was not observed after treatment of cells with PAM (Fig. 13C). This finding indicates a relationship between processes #1 and #2 to the long-lived species contained in PAM. It also is in line with the conclusion that process #3 seems to be directly triggered by ^1^O_2_ from the gaseous phase of CAP. This process cannot be triggered by PAM, due to the short-lived nature of ^1^O_2_.

Supplementary Fig. 1 demonstrates the independence of process #3 from NOX-derived O_2_^●−^, and ONOO^−^ during the 1 min CAP treatment, but its dependence on the primary ^1^O_2_ during CAP treatment. It also shows the action of a ^1^O_2_- and ONOO^−^-dependent step for more than one hour after the washing step that followed CAP treatment and separation of the cells from their treated medium. Finally, the strong inhibitory effect of the HOCl scavenger taurine added 4 h after the initial washing step showed that process #3 seems to be finalized by HOCl signaling.

Supplementary Fig. 2 shows that process #3 is also independent of the action of H_2_O_2_ during the 1 min of CAP treatment, as it was not inhibited in the presence of AEBSF and the catalase mimetic EUK-134. Also under these conditions, the process was dependent on ^1^O_2_ and ONOO^−^ after the washing step, in analogy to the results shown in Supplementary Fig. 1.

When CAP treatment of the cells was extended from 1 min to 5 minutes, the kinetics of apoptosis induction through process #3 started at 2 hours instead of 4 hours post treatment (Supplementary Fig. 3). The presence of the ^1^O_2_ scavenger histidine during CAP treatment, as well as after the washing step, confirmed that this process was initiated by ^1^O_2_ and required ^1^O_2_ for its kinetic propagation. The results are consistent with gas phase ^1^O_2_ triggering the effect. As expected, the onset of the kinetics is dependent on the time of CAP treatment. In contrast, ^1^O_2_ required for the propagation step must have been derived from the triggered tumor cells themselves.

### Quantification of the dynamics of singlet oxygen-mediated catalase inactivation in the cell population

The kinetic analysis of CAP-and PAM-mediated effects on tumor cells, as well as the mechanistic studies in the preceding manuscript^63^, have indicated that ^1^O_2_ generated through the interaction between long-lived RONS seemed to trigger the tumor cells to generate secondary ^1^O_2_ to an extend that causes extensive inactivation of membrane-associated catalase. This then seems to allow the reactivation of RONS-dependent intercellular apoptosis-inducing signaling. We therefore endeavoured to obtain quantitative data on the relative contribution of processes #1–3 to this autoamplificatory mechanism.

Autoamplification requires cell-to-cell communication, sometimes referred to as the “bystander effect”. Cells that did not experience the initial exposure of primary ^1^O_2_ and the associated inactivation of catalase must somehow be induced to undergo apoptosis. Our postulate is that this cell-to-cell bystander effect communication occurs because a few cells with inactivated catalase will generate enough H_2_O_2_ and ONOO^−^ to generate secondary ^1^O_2_ that will inactivate catalase on its neighboring cells. Due to their inactivated catalase, these neighboring cells are now allowed to generate their own secondary ^1^O_2_ and the process can continue in a kind of apoptotic wave. This postulate can be tested by pre-treating a subset of cells (thereby inactivating catalase and rendering them capable of generating secondary ^1^O_2_), then mixing and co-culturing these pre-treated cells with untreated cells. The judicious use of the various chemical species scavengers and enzyme inhibitors at different periods of the pre-treatment and co-culturing stages allows more refined testing of which species are key to the process.

Based on previous approaches that utilized siRNA-mediated knockdown of catalase^95^, the effect of direct ^1^O_2_ generators^96^, or high concentrations of H_2_O_2_^97^, as well as reconstitution experiments with defined concentrations of H_2_O_2_ and NO_2_^−^ ^60^ we utilized an experimental system that allows to define and characterize bystander signaling between CAP/PAM-pretreated cells and untreated tumor cells. It is based on the concept that apoptosis induction in mixtures of increasing concentrations of CAP/PAM-pretreated cells with untreated cells should merely be correlated to the percentage of pretreated cells if there was no bystander signaling. If bystander signaling occurred between pretreated and untreated cells, final apoptosis induction should be much higher than to be expected from the percentage of pretreated cells in the mixture. In other words, there should be an amplification effect: initially pre-treated cells co-cultured with untreated cells should induce the untreated cells to undergo apoptosis during co-culturing.

The validity and significance of this experimental system has been explicitely shown for mixtures of untreated tumor cells with increasing percentages of tumor cells that had been either pretreated by siRNA-mediated knockdown of their protective catalase^95^, inactivation of their protective catalase through ^1^O_2_ generated by an illuminated photosensitizer^96^, or by defined sources of H_2_O_2_ and NO_2_^−^ ^60^.

**Figure 14:**
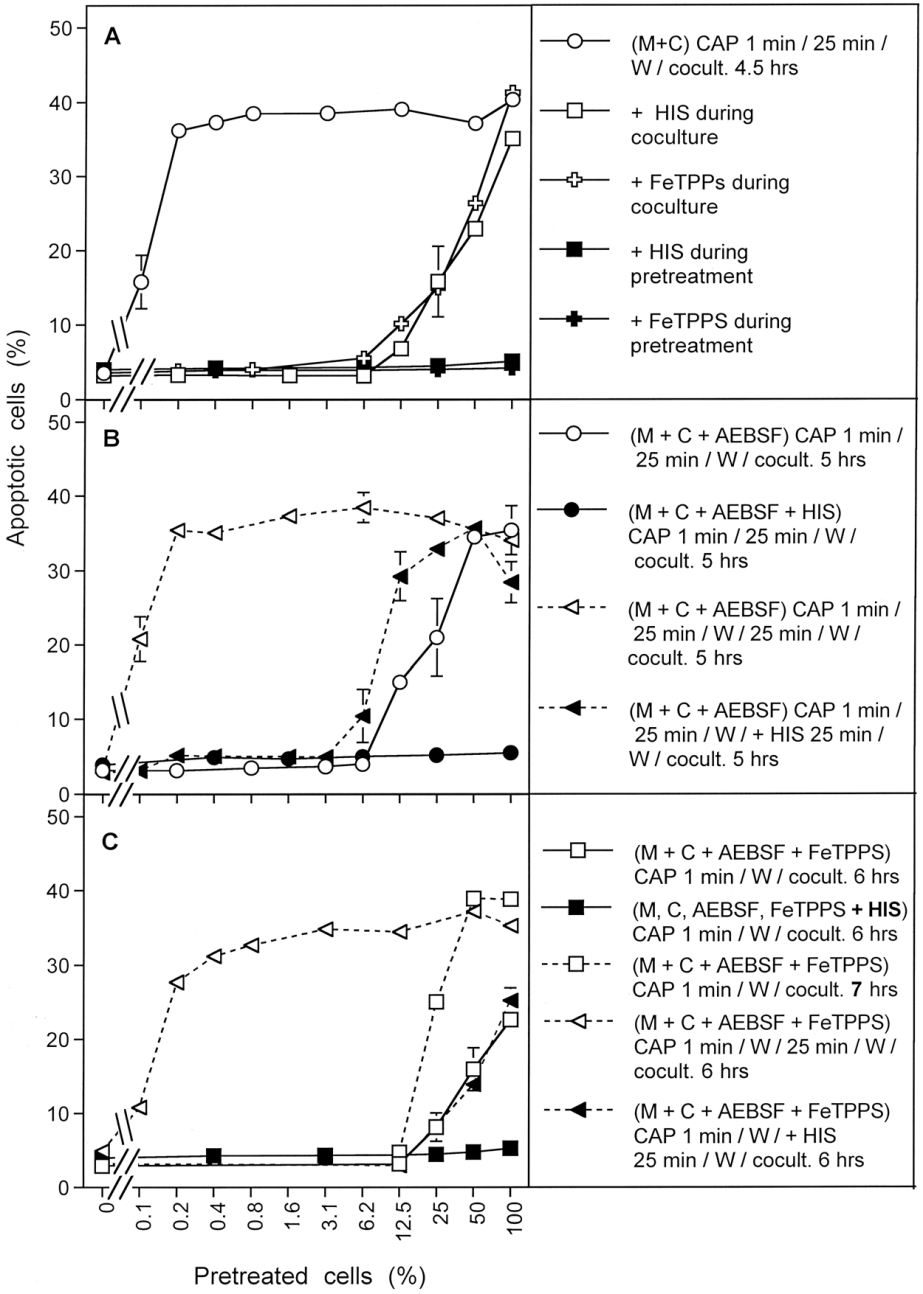
Bystander effects between CAP-treated and untreated tumor cells: a process controlled by singlet oxygen (^1^O_2_). (**A**) MKN-45 tumor cells were treated with CAP for 1 min, followed by 25 min incubation and a washing step (open circle), or histidine (“HIS”, 2 mM) (closed square) or FeTPPS (25 µM) (closed cross) was present during CAP treatment and subsequent incubation. After washing (W), the pretreated cells were mixed with untreated tumor cells at increasing percentages. In parallel, tumor cells pretreated with CAP, subsequent incubation and washing were added to untreated tumor cells and histidine (open square) or FeTPPS (open cross) were added. The assays were cultivated for 4.5 h. (**B**) MKN-45 cells in the presence of AEBSF (100 µM) (open circles) or AEBSF plus histidine (closed circles) were pretreated with CAP for 1 min, followed by 25 min incubation in the same medium and a subsequent washing step. The cells were then added to untreated tumor cells at increasing concentrations and cultivated for 5 h. Alternatively, tumor cells, pretreated with CAP + 25 min incubation in the presence of AEBSF were washed and then further incubated for 25 min (open triangle, dashed line), before they were washed again and added to untreated cells. In parallel, analogous assays received histidine during the second incubation step (closed triangles, dashed line). Apoptosis induction was determined after 5 h. (**C**) Tumor cells were treated with CAP for 1 min in the presence of AEBSF and FeTPPS. Immediately after the treatment, the cells were washed, resuspended in fresh medium and added to untreated tumor cells. Apoptosis induction was determined after 6 h (open squares) or 7 h (open squares, dashed line). In parallel, CAP treatment for 1 min was performed in the presence of histidine, in addition to AEBSF and FeTPPS (closed squares) and the assays were proceded as described above. In parallel assays, cells were treated with CAP for 1 min in the presence of AEBSF and FeTPPS, and were then immediately washed and respuspended in fresh medium. They were further incubated for 25 min either in the absence of inhibitors (open triangle, dashed line) or in the presence of histidine (closed triangle, dashed line). The cells were washed and added to untreated cells. Apoptosis induction was determined after 6 h.

When MKN-45 cells were pretreated with CAP for 1 min, followed by an incubation of 25 min in the same medium, and were then washed and added to untreated cells at increasing concentrations, a remarkable bystander effect on apoptosis induction was observed (Fig. 14A). The first of five experiments illustrated in Fig. 14A is pre-treating and co-culturing with no inhibitors (open circles), with varying percentages of pre-treated cells used in the co-culture stage. With zero percent pretreated cells, i. e. the pure population of untreated cells, no apoptosis above background was observed. With 100 percent pretreated cells, i. e. the pure population of pretreated cells, the reference (maximum) value of approximately 40% apoptotic cells was observed. This case corresponds to the previous experiments. The addition of only 0.1% pretreated cells (i. e. 12 cells) to an excess of untreated cells caused significant apoptosis induction. The presence of 0.2% pretreated cells (25 cells) was sufficient to induce the maximal effect of apoptosis induction in the cell population. This is clear evidence that only a few cells that have been activated by CAP or PAM exposure will trigger the maximal level of apoptosis in the entire populations of cells.

If our hypothesis regarding apopotic signaling is correct, then the presence of both ^1^O_2_ and ONOO^−^ are needed for this bystander signaling. The second and third sets of experiments in Fig. 14A involve the use of histidine (^1^O_2_ scavenger; closed squares) or FeTPPs (ONOO^−^ scavenger; closed crosses) during the pre-treatment stage. As can be seen in Fig. 14A, the presence of either histidine or FeTPPS during pretreatment completely prevented the bystander effect-inducing potential of the CAP-treated cells, indicating that ^1^O_2_ and ONOO^−^ were both necessary for achieving bystander effect-inducing potential of treated cells, consistent with the mechanistic hypothesis.

The third set of experiments in Fig. 14A involve the use of either histidine (open squares) or FeTPPS (open crosses) during the coculture of pretreated and untreated cells. Both of these inhibitors caused a marked reduction of apoptosis induction to values that were expected if only the pretreated cells took part in apoptosis induction. Only pre-treated cells underwent apoptosis. This finding indicates that the spread of bystander signaling from pretreated to untreated cells, and further on in the cell population, also depends on both ^1^O_2_ and ONOO^−^. This is consistent with the proposed hypothesis.

Figure 14B contains results from a set of four similar experiments in which the NOX1 inhibitor AEBSF was employed during pre-treatment. This approach prevents secondary singlet oxygen generation, but allows catalase inactivation by primary singlet oxygen derived from CAP-treated medium. When pretreatment of tumor cells with CAP and the subsequent 25 min incubation step were performed in the presence of AEBSF (open circles), and thus the contribution of NOX1-dependent generation of secondary ^1^O_2_ was prevented, subsequent bystander signaling of this population was quite low (Fig. 14B). The addition of 12.5% pretreated cells (1560 cells) to an excess of untreated cells was necessary to obtain significant apoptosis induction. Maximal apoptosis induction was obtained when pretreated and untreated cells were equally mixed. The second set of experiments in Fig. 14B show that the presence of histidine during pretreatment (filled circles) prevented this poor bystander effect. This result is consistent with the experiments with histidine only added during pre-treatment, shown by the filled squares in Fig. 14A. Histidine presence and therefore ^1^O_2_ absence during pre-treatment (or co-culturing) stops any bystander effect signaling.

An alternative explanation for residual apoptosis-inducing potential despite the presence of AEBSF might be based on a hypothetical insufficient inhibition of NOX1 by AEBSF. This explanation has been experimentally excluded through the quantitation of NOX1 inhibition and through experiments in which tumor cell apoptosis was initiated by NO-mediated catalase inhibition, in the absence of primary ^1^O_2_ and fully dependent on secondary ^1^O_2_. Under these conditions, complete inhibition by AEBSF was achieved, demonstrating the strong inhibitory potential of AEBSF^60^.

In the third set of experiments shown in Fig. 14B, cells were pre-treated with CAP in the presence of AEBSF and further incubated in the presence of AEBSF for 25 minutes. The cells were then washed and incubated an additional 25 minutes in the absence of AEBSF and then were washed a second time (open triangles). With this additional incubation in the absence of AEBSF and second washing step added, the full potential for induction of bystander signaling of the cell population was recovered. Presumably, during the second incubation secondary ^1^O_2_ generation had resumed, due to the absence of the NOX1 inhibitor. It seems, that starting from the very low population of cells that had been triggered by CAP-derived primary ^1^O_2_ under conditions of prevention of secondary ^1^O_2_ generation, bystander signaling had efficiently caused autoamplification of secondary ^1^O_2_ generation, catalase inactivation and the potential for apoptosis induction. Therefore, it was possible to transmit maximal apoptosis induction with only 0.2% added pretreated cells.

In the final set of experiments shown in Fig. 14B (solid triangles), the experimental procedure was identical to the one used for open triangles (i.e. a second AEBSF-free incubation and washing after pre-treatment and first incubation in the presence of AEBSF) except now the ^1^O_2_ scavenger histidine was added during the second 25 minute incubation. The cells were then washed a second time. This addition of histidine during the second incubation followed by washing to remove this histidine significantly reduced bystander signaling compared to the case with no histidine added in the second incubation step. These findings indicate that the strong increase in bystander induction during the second (AEBSF-free) incubation was based on a singlet oxygen- and O_2_^●−^ -dependent mechanism, presumably autoamplification of secondary ^1^O_2_ generation by the tumor cells.

Based on our working hypothesis, one would predict that the curves characterized by open circles and closed trigangles in Fig. 14B should be congruent. The slight difference between these two curves is explained by an unavoidable technical problem: in the case characterized by the closed triangles, the cells are subject to an additional washing step (including three cycles of washing), during which secondary ^1^O_2_ generation can already resume, as histidine is only added after this step.

Figure 14C adds the effects of ONOO^−^ inhibition with the addition of FeTPPs during pre-treatment, coupled with elimination of the incubation period following pre-treatment and changes in incubation time for bystander signaling. Under these conditions, potential effects of primary ^1^O_2_ generated by long-lived species in CAP-are prevented, as ONOO^−^, a necessary intermediate for the generation of primary ^1^O_2_ from long-lived species was decomposed and as the time of contact between CAP-treated medium and cells was minimized to 1 minute. In addition, the presence of AEBSF prevented the generation of secondary ^1^O_2_ before the washing step.

Tumor cells pretreated with CAP in the presence of AEBSF and FeTPPS, and that had been separated from the medium immediately after CAP treatment (open squares with solid lines), showed no potential for the induction of bystander signaling within 6 hrs of incubation, but went into apoptosis by themselves. Apoptosis induction was completely prevented when the ^1^O_2_ scavenger histidine had been present during CAP treatment (solid squares), indicating that ^1^O_2_ was involved in the initiation of this process. An increase in the coculture time to 7 hrs (open squares, dashed lines) allowed for an increase in apoptosis induction, but not in bystander signaling. However, pretreatment of the cells with CAP in the presence of AEBSF and FeTPPS for 1 minute, followed by a washing step and incubation in inhibitor-free medium for 25 min (open triangles), allowed to unleash the bystander effect inducing potential of these cells in an impressive way. This induction of bystander signaling was prevented in the presence of the ^1^O_2_ scavenger histidine during the 25 min incubation step (solid triangles). Taken together, these data show that singlet oxygen derived from the gaseous phase of CAP only can induce bystander signaling if it has a chance to induce the generation of secondary ^1^O_2_ before the coculture step with untreated tumor cells.

These data were confirmed by the experiment shown in Supplementary Fig. 4.

This figure also illustrates the impact of the time of coculture on apoptosis induction and the dependence of bystander signaling on the time of contact between the tumor cells and medium containing long-lived species derived from CAP treatment.

**Figure 15:**
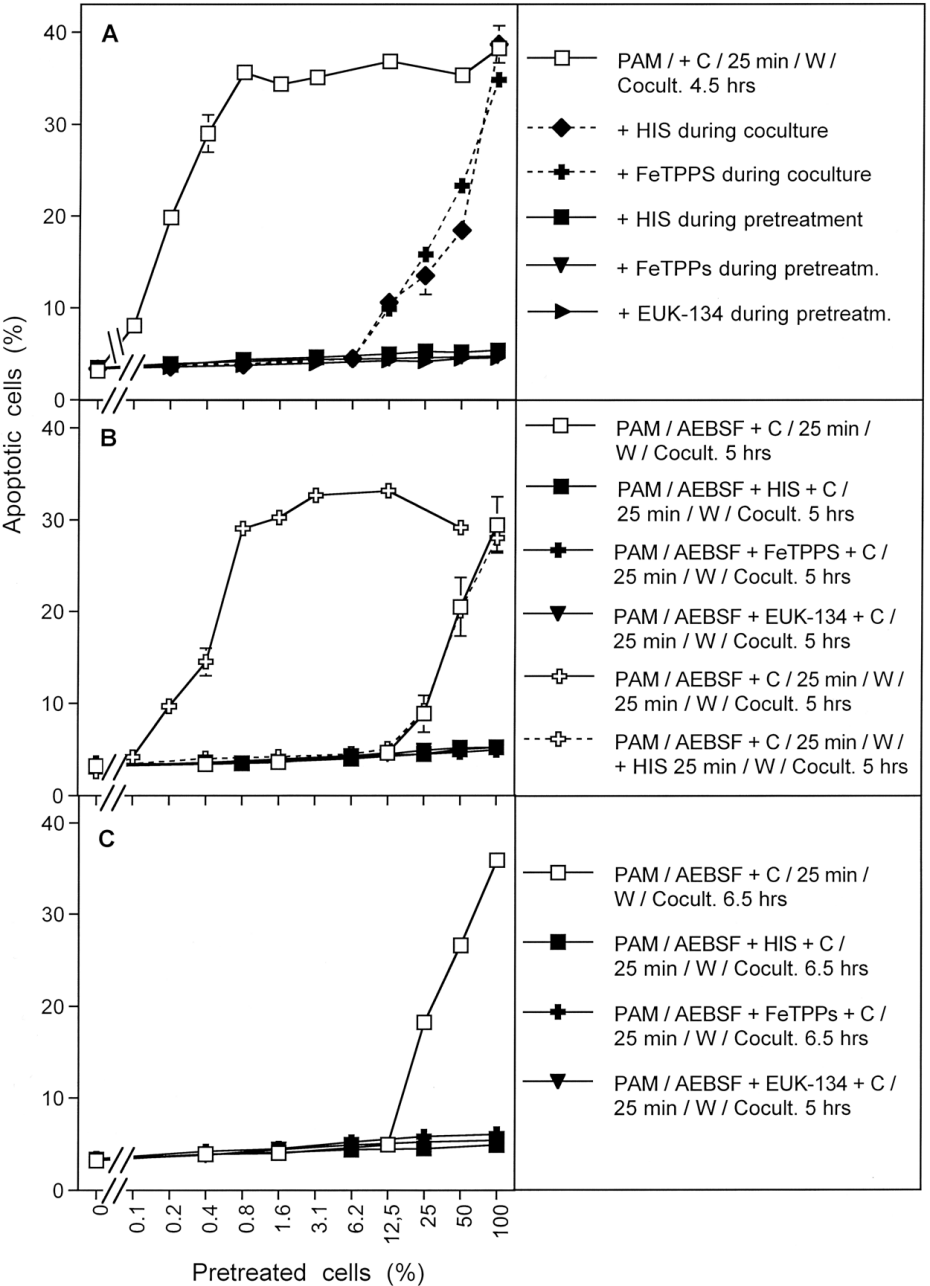
Bystander effect-inducing potential after treatment of tumor cells with PAM. (**A**) Medium was treated with CAP for 1 min. The resultant PAM was added to cells and the mixture was incubated for 25 min. After a washing step of three cycles, increasing percentages of pretreated cells were added to untreated cells and the percentages of apoptotic cells were determined after 4.5 h. Parallel assays were performed analogously, with the modification that histidine (“HIS”, 2mM) or FeTPPS (25 µM) or EUK-134 (25 µM) were either present during pretreatment of cells with CAP, or during the coculture between pretreated and untreated cells. (**B**) PAM was mixed with tumor cells and AEBSF was added to all assays. Then the assays received either no further addition, or addition of 2 mM histidine, 25 µM FeTPPS or 25 µM EUK-134. All assays were incubated for 25 min and then washed. Increasing concentrations of pretreated cells were added to untreated cells and the percentages of apoptotic cells were determined after 5 h In additional assays, coculture of PAM and cells in the presence of AEBSF was performed for 25 minutes. Then the cells were washed, resuspended in fresh medium and incubated for 25 min in the absence of inhibitors (open cross) or in the presence of histidine (open cross, dashed line). Then the cells were washed again and added to untreated cells at increasing concentrations. Apoptosis induction was determined after 5 h. (**C**) The assays defined by open squares, closed squares, closed crosses and closed triangles under B were cultivated for 6.5 h and then apoptosis induction was determined. These data show that PAM induces strong bystander inducing potential, provided the generation of primary and secondary ^1^O_2_ is not prevented. Prevention of secondary ^1^O_2_ caused a dramatic loss of bystander inducing cells. A high potential can, however, be recovered, when AEBSF is removed from the cells and an incubation step of 25 min is allowed in fresh medium. The recovery of the inducing potential is mediated by ^1^O_2_.

The results shown in Fig. 15 summarize analogous experiments made following PAM exposure. When tumor cells were treated with PAM for 25 minutes, a strong bystander effect-inducing potential in the cell population was observed (Fig. 15A; open squares). The presence of the ^1^O_2_ scavenger histidine, the ONOO^−^ decomposition catalyst FeTPPS and the H_2_O_2_ decomposition catalyst EUK-134 during pre-treatment of tumor cells with PAM completely prevented the establishment of their potential for apoptosis induction as well as for inducing bystander signaling. This finding is consistent with the concept that PAM effects on tumor cells are mediated by ^1^O_2_ which is generated through the interaction between the long-lived species H_2_O_2_ and NO_2_^−^, with ONOO^−^ as an intermediate. When histidine or FeTPPS were present during coculture, bystander signaling was inhibited and only apoptosis induction of the pretreated subpopulation in the cocultures was observed. This finding confirms the role of ^1^O_2_ and ONOO^−^ for bystander signaling.

Figure 15B shows that the presence of the NOX1 inhibitor AEBSF during pre-treatment of the tumor cells with PAM (open squares) allowed for subsequent apoptosis induction in the pretreated subpopulation, but not for bystander signaling. This finding points to the role of NOX1 derived O_2_^●−^ for the establishment of bystander signaling, presumably based on the generation of secondary ^1^O_2_. The apoptosis-mediating effect of PAM under these conditions seemed to be depending on ^1^O_2,_ ONOO^−^ and H_2_O_2_, as demonstrated by the strong inhibitory effects by histidine, FeTPPS and EUK-134. This finding confirms the role of ^1^O_2_ generated by PAM, based on the interaction between H_2_O_2_ and ONOO^−^. When tumor cells had been pretreated with PAM for 25 min in the presence of AEBSF, and then were subjected to a second incubation for 25 min in the absence of AEBSF, a strong bystander effect-inducing potential of these cells was observed (open crosses, solid line, in Fig. 15B). The induction of this potential was completely prevented when the ^1^O_2_ scavenger histidine was present during the AEBSF-free incubation (open crosses, dashed line). These findings show that PAM imprints a signature on tumor cells through the action of ^1^O_2_. This imprinted signature seems to allow the generation of secondary ^1^O_2_ and impressive bystander signaling after removal of the NOX1 inhibitor. This finding further confirms the role of NOX1-derived O_2_^●−^ for bystander signaling. The central findings for AEBSF-pretreated cells were also confirmed after extension of the time of coculture up to 6.5 hours and were completely attributed to primary ^1^O_2_ derived from long-lived species in PAM, without any indication for an effect by ^1^O_2_ derived from the gaseous phase of CAP. The latter finding is different for the findings obtained after CAP treatment (Fig. 14) and is consistent with the very short life time of ^1^O_2_.

**Figure 16:**
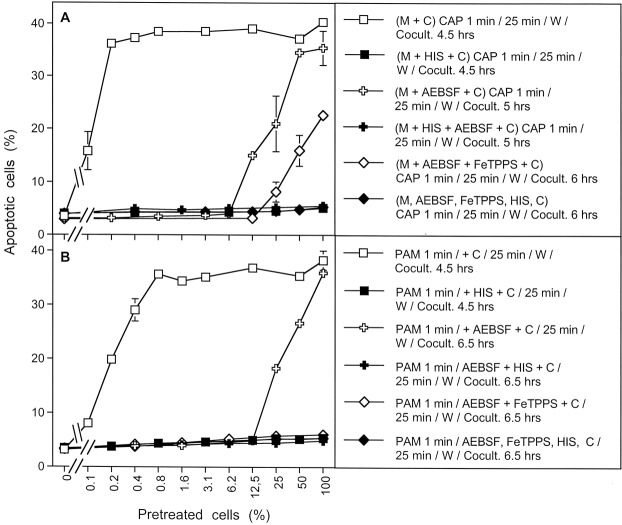
Induction of bystander effects by CAP and PAM. (**A**) MKN-45 cells were treated with CAP for 1 min, followed by an incubation step of 25 min in the same medium. After a washing step of three cycles, increasing percentages of pretreated cells were added to untreated tumor cells (open squares). The percentages of apoptotic cells in these cocultures were determined at the indicated times. This basis approach was modified by either adding the NOX1 inhibitor AEBSF (100 µM) during CAP treatment and initial incubation (open crosses), and in this way blocking the generation of secondary ^1^O_2_, or adding AEBSF and the ONOO^−^ decomposition catalyst FeTPPS (25 µM) (open diamonds), and in this way allowing to focus on ^1^O_2_ derived directly from the gaseous phase of CAP, as the generation of ^1^O_2_ from long-lived species in the medium and secondary ^1^O_2_ generation by the cells was prevented under these specific conditions. All modifications were studied in parallel in the presence of histidine during pretreatment (closed symbols) to allow the evaluation of the role of ^1^O_2_. (**B**) The experiment was performed in an analogous mode to A, with the exception that the cells were not treated with CAP in medium, but were mixed (50%) with PAM that had been prepared by treating medium with CAP for 1 min. The results show that maximal bystander inducing potential is achieved when CAP or PAM treatment is applied without experimental interference with primary or secondary ^1^O_2_ generation (open squares). Prevention of secondary ^1^O_2_ generation causes a dramatic loss of bystander effect-inducing cells both after CAP and PAM treatment (open crosses). This effect is essentially due to primary ^1^O_2_ generated by long-lived species (i. e. H_2_O_2_ and NO_2_^−^). Direct treatment of cells in medium in the presence of AEBSF and FeTPPS (Fig. 11A) allows to detect the effect of ^1^O_2_ derived from the gaseous phase of CAP (open diamonds) which is lower than the effect by ^1^O_2_ generated by long-lived species (open crosses). In assays treated with the PAM regime (Fig. 11B), no effects due to ^1^O_2_ derived from the gaseous phase of CAP were detected, consistent with the short life time of ^1^O_2_.

The direct comparison of the effects of CAP and PAM on bystander signaling shows that the induction of bystander signaling was strong for conditions that correlated with process #1 (open squares) as determined in the preceding kinetic analysis, both for CAP and PAM-treated tumor cells (Fig. 16). When NOX1 had been blocked by AEBSF initially and thus process #2 was set (open crosses), apoptosis induction of the pretreated cells but no significant bystander effect was observed for CAP and PAM pretreatment. In contrast, apoptosis induction through process #3 (open diamonds) was only observed in the case of CAP treatment and was completely lacking after PAM treatment, as well as in cells treated with CAP in the presence of the ^1^O_2_ scavenger histidine. These findings indicate that process #3 is triggered by ^1^O_2_ from the gaseous phase of CAP.

Supplementary Figs 5 and 6 extend the analysis of bystander signaling through processes #1–3 and analyze the role of the length of CAP or PAM pretreatment for the intensity of the effect observed.

**Figure 17:**
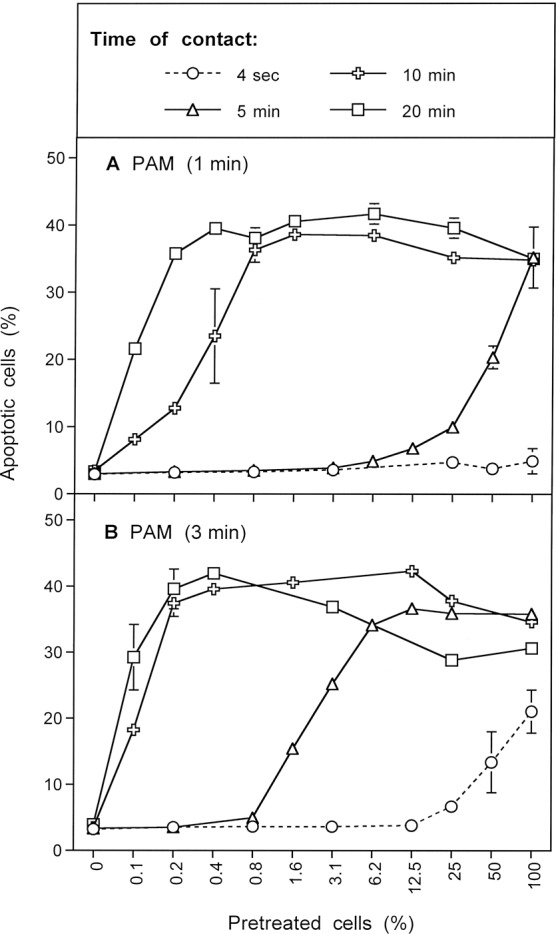
Dependency of induction of bystander inducing potential of tumor cells on the preparation of PAM and on the time of contact between PAM and cells. PAM was generated by treatment of the medium with CAP for 1 min (**A**) or 3 minutes (**B**). MKN-45 tumor cells were mixed with PAM (50%) for the indicated times of contact and then washed by three cycles. Increasing percentages of pretreated cells were mixed with untreated tumor cells and the percentages of apoptotic cells in the cocultures were determined 4.5 hours after the beginning of the coculture. The results show that the induction of bystander inducing potential depends on the time of CAP treatment during the preparation of PAM and on the time of contact between PAM and the tumor cells. This is in line with the assumption that CAP generates long-lived species in PAM in a time-dependent fashion and that these compounds need to interact among themselves and with the cells in a time-dependent way.

**Figure 18:**
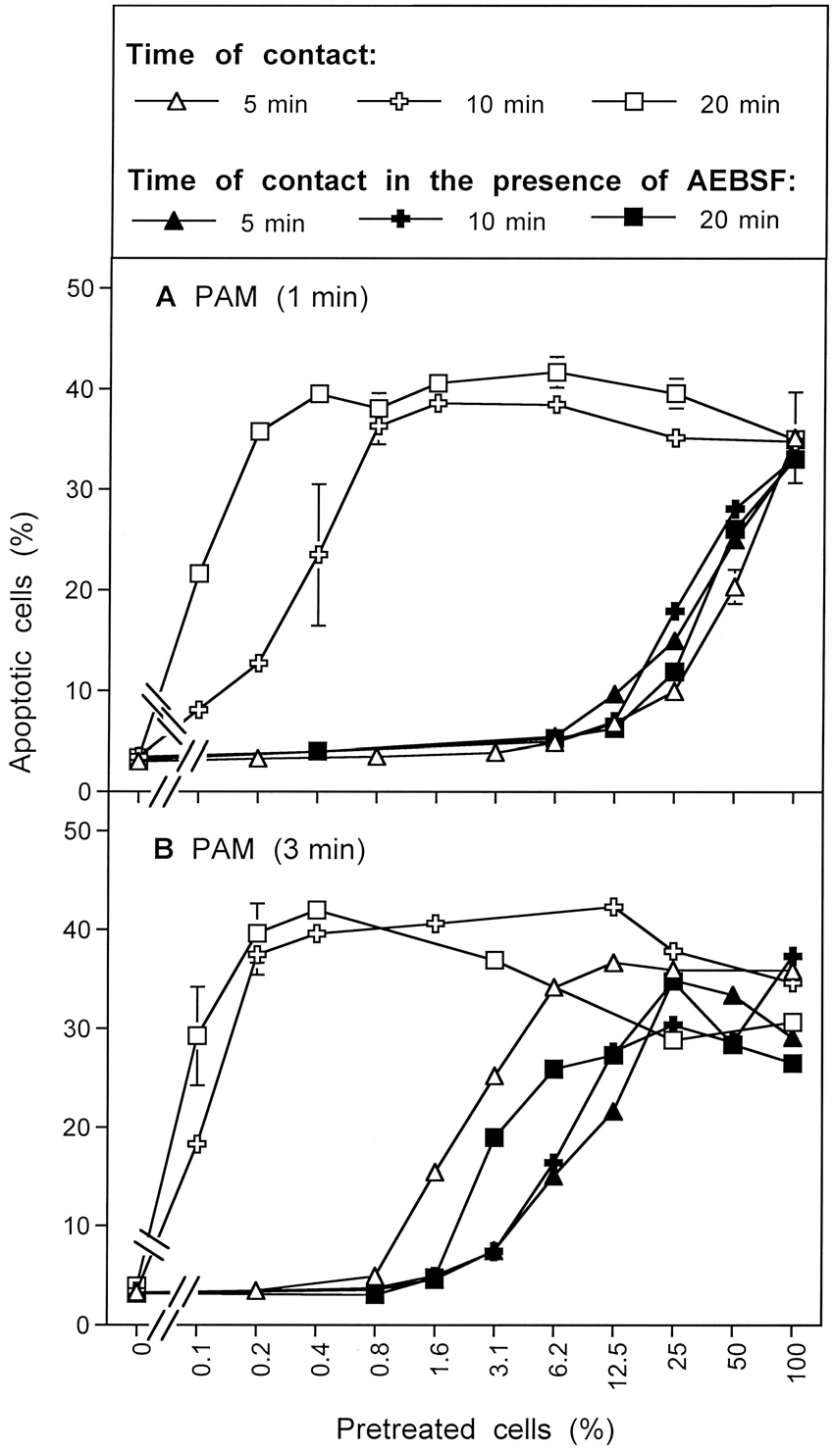
Dependency of the induction of bystander effect inducing potential on the generation of secondary singlet oxygen (^1^O_2_). The experiment described in Fig. 12 is presented here, with the addition that 100 µM AEBSF had been present during the time of contact between PAM and cells. In this way the generation of secondary ^1^O_2_ was prevented specifically during the time of contact, but not after the washing step and during final incubation. The separation of the data into two figures has been chosen to allow an easier evaluation by the reader. The results show the dominating effect of secondary ^1^O_2_ generation for the induction of bystander effect inducing potential, on top of the initial effect of primary ^1^O_2_ from PAM.

Figure 17 illustrates that the induction of apoptosis as well as expression of bystander effect-inducing potential of PAM-treated tumor cells depends on the length of treatment of medium with CAP and the time of contact between tumor cells and the activated medium. When NOX1 of tumor cells was blocked by AEBSF, induction of bystander effect inducing potential was strongly reduced (Fig. 18). Therefore, it was only observable for PAM generated through 3 min treatment with CAP, whereas it was not significant in PAM generated through CAP treatment of 1 min only (Fig. 18).

### Decrease in PAM efficiency by tumor cells

**Figure 19:**
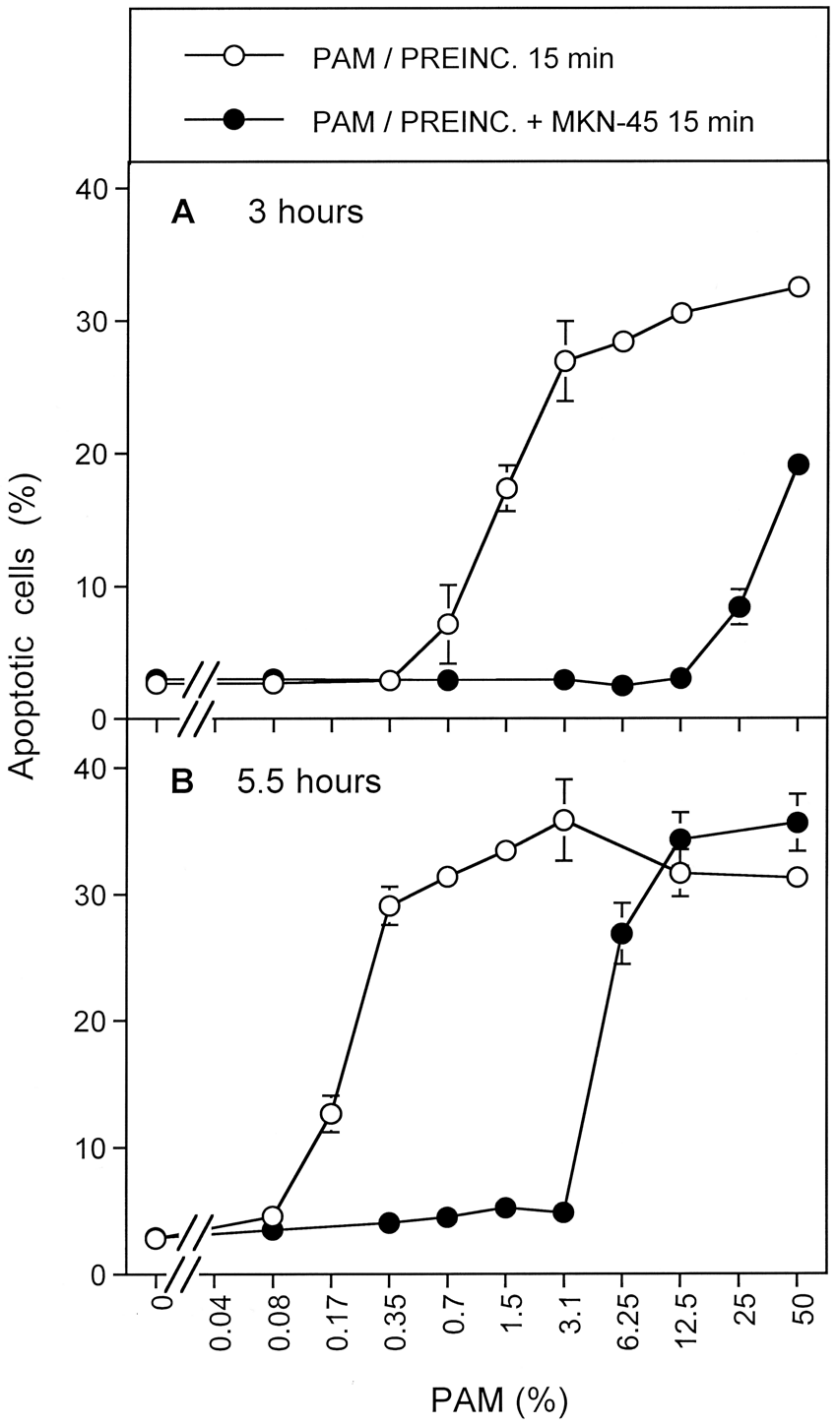
Consumption of PAM constituents by tumor cells. PAM was generated by CAP treatment of medium for 1 min. PAM was preincubated (stored) either in the absence of cells (open circle) or in the presence of MKN-45 at standard density (closed circles) for 15 min. The preparations were then centrifuged to remove the cells or to perform mock treatment, and the supernatants were added at increasing concentrations to fresh MKN-45 cells. The percentages of apoptotic cells in this fresh group were determined after 3 h (**A**) and 5.5 h (**B**). The result shows that pretreatment of PAM with tumor cells substantially lowers the potential of PAM to induce apoptosis in the fresh cells. The most likely explanation is the consumption of H_2_O_2_ by membrane-associated catalase, and potentially also the oxidation of NO_2_^−^ to ^●^NO_2_ by catalase.

When PAM was incubated with tumor cells for 15 min before it was applied to fresh tumor cells, its efficiency on the fresh tumor cells was decreased by more than 90 percent, compared to PAM incubated in the absence of cells (Fig. 19). This finding illustrates that besides the action of PAM on tumor cells, as characterized in the preceding experiments, there is also a strong impact of the cells on the potential of PAM to trigger sensitization of tumor cells for catalase inactivation and apoptosis induction. The dynamics of PAM decomposition by the cells is therefore in competition with PAM effects on the cells, creating a highly dynamic and complex system of interactions. Based on established chemical biology, PAM decomposition by the tumor cells may reflect decomposition of H_2_O_2_ and oxidation of NO_2_^−^ to ^●^NO_2_. Both processes can be catalyzed by catalase^74,98^.

### Statistical analysis of the results

Figure 8: (4 h): Apoptosis induction in assays B, D, E was highly significant (p < 0.001), whereas there was no significant apoptosis induction in C, F, G. II (6 h): Apoptosis induction in B,C,D, E was highly significant (p < 0.001), without significant differences between these assays. III (7.5 h): Apoptosis induction in assays B-G was highly significant (p < 0.001). The difference between apoptosis induction in the group B-E compared to F and G was highly significant (p < 0.001).

Figure 9: A: Apoptosis induction in CAP-treated assays without inhibitors was highly significant at 2 h and later (p < 0.001). The effect of all inhibitors was highly significant (p < 0.001). B: The shift in the kinetics between assays treated with CAP in the absence of inhibitors, in the presence of AEBSF or in the presence of FeTPPS was highly significant (p < 0.001). C: Apoptosis induction was highly significant at 6 h and later (p < 0.001), without significant difference between the assays.

Figure 10: A: Apoptosis induction reached by the different groups after 1 h, 4 h and 6 h respectively was highly significant (p < 0.001). The kinetic differences between the groups were highly significant (p < 0.001). Assays containing L-NAME did not show significant apoptosis induction. B: The inhibitory effects of histidine were highly significant (p < 0.001). C: Apoptosis induction in the absence of histidine was highly significant at 6 h and later (p < 0.001) without significant differences between the two groups of assays. The inhibitory effects of histidine were highly significant (p < 0.001)

Figure 11: A: Inhibition of CAP-mediated apoptosis induction by histidine added during CAP treatment was highly significant (p < 0.001), whereas addition of histidine or FeTPPS after the washing step caused no significant inhibition. B: The kinetic differences observed were highly significant (p < 0.001). C. Apoptosis induction in assays described by open symbols was significant at 6 h and later (p < 0.001), without significant differences between the two groups. The difference between assays described by open and closed symbols was significant (p < 0.001).

Figure 12: A–C: Apoptosis induction in all assays described by open symbols was significant at 6 h and later (p < 0.001), as were all inhibition curves described by closed circles (p < 0.001).

Figure 13: A: Apoptosis induction in the assays described by open symbols, the shift between the curves with open squares and open crosses, as well as inhibition of these processes by histidine (closed symbols) were significant (p < 0.001). B: The addition of histidine to PAM-treated cells after the washing step did not cause significant inhibition, whereas addition of histidine (after the washing step) to cells incubated in the presence of PAM and AEBSF caused highly significant inhibition of apoptosis (p < 0.001). C. The inhibitory effect of FeTPPS present during PAM treatment was highly significant (p < 0.001).

Figure 14: A: Apoptosis induction in assays containing at least 0.1% pretreated cells was highly significant (p < 0.001). Inhibition by histidine or FeTPPS added during pretreatment or during coculture was highly significant (p < 0.001). B: The differences between curves characterized by a) open triangles and open circles, b) open and closed circles and c) open and closed triangles were highly significant (p < 0.001). C: The differences between curves characterized by a) open squares and open triangles, b) open and closed triangles or squares were highly significant (p < 0.001).

Figure 15: A: Apoptosis induction in assays containing at least 0.2% pretreated cells was highly significant (p < 0.001). Inhibition by histidine or FeTPPS present during pretreatment or coculture was highly significant (p < 0.001). B: The differences between the curves characterized by open crosses (solid line) and open squares or open crosses (dashed line) were highly significant (p < 0.001). The inhibitory effects by histidine, FeTPPS and EUK-134 were highly significant (p < 0.001). C: Open squares: apoptosis induction was highly significant at 25% pretreated cells and higher (p < 0.001). The effect of all inhibitors was highly significant (p < 0.001).

Figure 16: A: Apoptosis induction reached by curves characterized with open symbols was highly significant at 0.1%, 12.5% or 50% respectively. Inhibition by histidine was always highly significant (p < 0.001). B: Apoptosis induction reached by curves characterized with open symbols was highly significant at 0.2%, or 25% respectively. Inhibition by histidine was always highly significant (p < 0.001).

Figure 17: A: Curves characterized by open squares, crosses and triangles reach highly significant apoptosis induction at 0.1%, 0.2% or 50% pretreated cells respectively. The differences in the response to increasing concentrations of pretreated cells were highly significant. B: Curves characterized by open squares and open crosses were not significantly different and reach significant apoptosis induction at 0.1% pretreated cells. Apoptosis induction reached by curves characterized with open symbols was highly significant at 0.1%, 12.5% or 50% respectively. Inhibition by histidine was always highly significant (p < 0.001). Curves characterized by open triangles and open circles reach significant apoptosis induction at 1.6 or 50% pretreated cells, respectively Apoptosis induction reached by curves characterized with open symbols was highly significant at 0.1%, 12.5% or 50% respectively. Inhibition by histidine was always highly significant (p < 0.001). The differences between the corresponding curves in A and B were highly significant Apoptosis induction reached by curves characterized with open symbols was highly significant at 0.1%, 12.5% or 50% respectively. Inhibition by histidine was always highly significant (p < 0.001), except for the curves describing 20 min of contact time.

Figure 18: The effects of AEBSF were highly significant for all assays (p < 0.001), except for 5 min treatment time under A.

Figure 19: All curves reach statistically significant values for apoptosis induction. The differences between the curves with open and closed circles were highly significant (p < 0.001).

## Discussion

The established scheme on the generation of primary and secondary ^1^O_2_ through, defined CAP- and PAM-derived compounds^59,60^, the availability of scavengers and inhibitors that interfere with defined players in this signaling system (Fig. 1), and the possibility to dissect the system into defined steps or to reconstitute defined conditions^59,60^, allowed us to elucidate the dynamics of CAP and PAM-mediated apoptosis induction in tumor cells.

Treatment of cells with CAP in the presence of medium, or initial treatment of medium, followed by transfer of this plasma-activated medium (PAM) to cells, allowed to elucidate defined signaling processes, when this treatment was combined with the variation of incubation times, washing steps, as well as the addition or removal of inhibitors and dissecting/reconstituting the experimental system.

Besides the insights into the biochemistry and dynamics of CAP- and PAM-triggered apoptosis induction in tumor cells, this approach also strenghtened the view that “direct treatment” of tumor cells with CAP and treatment of tumor cells with PAM are strongly overlapping in the mode of their action, provided that tumor cells are treated with CAP under standard cell culture conditions, where the cells are overlaid by medium. This setup was essential for the function of the plasma source used in our studies. Under these conditions, highly reactive species from CAP seem to react with medium components before they have a chance to reach the target cells. Therefore, the approach used in our studies is focusing on the long-lived species which are generated by CAP and survive in PAM.

It shows that the long-lived species are sufficient to explain the known effects of CAP and PAM on tumor cells, but does not exclude that if CAP-generated gas phase species interact directly with cell membrane surfaces, additional species and pathways may contribute to tumor cell death as well.

Several key conclusions were established from our biochemical analysis:

- Primary ^1^O_2_ generated by long-lived species, resultant from CAP treatment of medium, is sufficient to trigger the onset of efficient apoptosis induction in tumor cells, provided there is a sustained generation of secondary ^1^O_2_ (driven by tumor cell NOX1 and NOS) following the original primary ^1^O_2_ trigger. This conclusion is essentially based on the analogous effects of the direct CAP treatment of cells in medium and the treatment of cells with plasma-activated medium. This conclusion is in good agreement with the findings by Canal *et al*.^35^, Koensgen *et al*.^50^ and Liedtke *et al*.^36^ and many others, who described a sustained effect of long-lived species derived from CAP and PAM on tumor cells.
- The effect of the long-lived species is sufficiently explained by the reaction scheme shown in Fig. 1, which starts with the interaction between NO_2_^−^ and H_2_O_2,_ and leads to the generation of primary ^1^O_2_ through decomposition of peroxynitrate (O_2_NOO^−^). As a consequence of primary ^1^O_2,_ local inactivation of membrane-associated catalase is achieved and leads to the sustained generation of secondary singlet oxygen, starting with the interaction between H_2_O_2_ and ONOO^−^ generated by the tumor cells. This conclusion is essentially based on the results of inhibition studies with inhibitors/scavengers that target central steps in the scheme shown in Fig. 1 ^59,60^.
- When the generation of secondary ^1^O_2_ is initially inhibited, local catalase inactivation by primary ^1^O_2_ derived from NO_2_^−^/H_2_O_2_ interaction leaves an imprinted “signature” on the cells that allows them to resume secondary ^1^O_2_ generation after removal of the specific inhibitor. As a result, although apoptosis induction is delayed, apoptosis induction in the cell population is ultimately effective. This shows the astonishing dynamics that is inherent to this biological system. These conclusions are mainly based on kinetic analysis, including inhibition of NOX1 for defined periods and on reconstitution experiments. These experiments have been performed in this study and are confirmed by a study that utilized defined chemicals rather than a plasma source^59,60^.
- Tumor cells taken from an assay that had been pretreated with CAP or PAM induce ^1^O_2 _-dependent amplification of secondary ^1^O_2_ generation and catalase inactivation in a population of untreated tumor cells with high efficiency. These reconstitution experiments, based on bystander signaling, allowed the identification of the biochemical mechanism underlying the individual steps of this process and to quantify the frequencies of “imprinting” through primary and secondary ^1^O_2._
- As less than 1% of tumor cells pretreated with CAP or PAM were sufficient to cause cell death of the surrounding 99% of untreated tumor cells, the essential contribution of the cells to their own cell death is unequivocally proven. It is demonstrated, that CAP and PAM are simply needed as a trigger to initiate this selective process.
- In addition to primary ^1^O_2_ generated in liquid phase by NO_2_^−^/H_2_O_2_ with ONOO^−^ as intermediate, the action of primary ^1^O_2_ derived from the gaseous phase of CAP can be detected. This approach requires to establish conditions in which the generation of ^1^O_2_ from H_2_O_2_/NO_2_^−^ and the generation of secondary ^1^O_2_ by the cells had been prevented. In addition, it was only successful with tumor cells grown at least partially in suspension, thus increasing the chance of CAP-derived ^1^O_2_ to reach at least a few cells. This experimental approach and its results are visualized in more details in Supplementary Figs 19–22.

Direct ^1^O_2_ from the gaseous phase of CAP plays a minor role compared to ^1^O_2_ generated by the long-lived species, and therefore is not determining the overall reaction under the conditions of our assays and with the specific plasma source used. In line with this conclusion, direct treatment of medium containing cell cultures with CAP (followed by an incubation step) is not more efficient than treatment of cells with PAM, in which direct ^1^O_2_ from CAP is missing due to its limited life time. However, when long-lived species in CAP-treated medium and their reaction products were catalytically decomposed, the effect of ^1^O_2_ from the gaseous phase of CAP on tumor cells can be specifically demonstrated and analyzed. This effect was also dependent on the subsequent generation of secondary ^1^O_2._ It was characterized by a rather delayed kinetics. This is explained by a starting point of this particular process, in which very few tumor cells had been imprinted and therefore more time was required for the autoamplication process. Please see Supplementary Figs 19–22 for visualization of this scenario.

Importantly, the biological effect of the direct primary ^1^O_2_ from the gaseous phase of CAP is triggered independent of ONOO^−^, but requires cell-derived ONOO^−^ for the subsequent step of formation of secondary ^1^O_2_. Furthermore, the primary ^1^O_2_ from the gaseous phase of CAP has been shown to only affect tumor cells in suspension, but not monolayers of tumor cells. This is due to the short free diffusion path length of ^1^O_2_. It can be predicted that an increase of the concentration of ^1^O_2_ in the gaseous phase of CAP, combined with a lower level of medium covering the tumor cells, might substantially increase the chance of ^1^O_2_ from the gaseous phase to trigger apoptosis induction in tumor cells. Based on model experiments and on the data obtained in this study, it can be predicted that the biological effect of directly acting ^1^O_2_ from the gaseous phase should utilize the same biochemical mechanisms as ^1^O_2_ generated by the long-lived species in CAP and PAM.

All of these initial conclusions were confirmed in a parallel reconstitution approach, in which the effects of defined concentrations of H_2_O_2_ and NO_2_^−^ towards tumor cells were studied, thereby mimicking essentially the effect of PAM^59,60^. The significance and validity of these reconstitution experiments is ensured, as a study in which an illuminated photosensitizer was used as a defined source of ^1^O_2_ led to congruent results and conclusions^96^.

Previous work^60,^^63^, as well as the kinetic approach of this study, revealed that the primary ^1^O_2_ triggers a rare process that allows for sustained, highly efficient and fast generation of the secondary ^1^O_2_. The interaction of the primary as well as the secondary ^1^O_2_ with the catalase on the membrane of tumor cells retains a signature that seems to allow this cell to autoamplify ^1^O_2_ generation. The nature of this imprinted signature is inactivation of membrane-associated catalase.

The induction of this signature solely by primary ^1^O_2_, under conditions of inhibited secondary ^1^O_2_ generation is proven to be a rare process, as very large numbers of cells pretreated under these specific conditions have to be transfered to an untreated cell population in order to establish bystander signaling. It seems that only few tumor cells in the cell population pretreated under these conditions are the inducers of bystander signaling. Therefore, it is unlikely that several catalase molecules on the same cell have been inactivated.

This finding points to the inducing power of individual inactivated catalase molecules.

When, however, the action of primary ^1^O_2_ was immediately inducing the generation of secondary ^1^O_2_, neighbouring catalase molecules on the same cell and on neigbouring cells seem to be inactived and rapidly propagated the bystander signaling. As a result, after few minutes past initial treatment, only few cells taken from a pretreated cell population were sufficient to transmit the bystander signaling to an untreated cell population.

**Figure 20:**
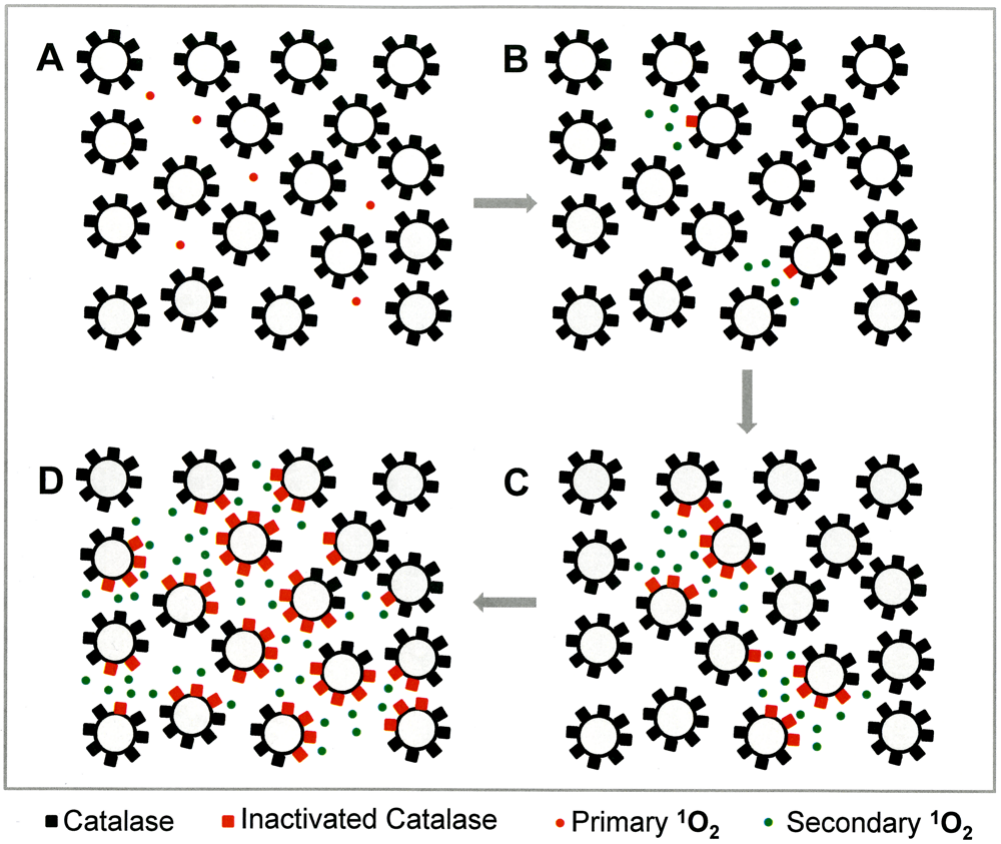
The principle of bystander signaling. Primary singlet oxygen (^1^O_2_) generated through the interaction of long-lived species in CAP-treated medium or directly derived from the gaseous phase of CAP (red dots) (**A**) causes local inactivation of membrane-associated catalase on very few tumor cells (**B**). Subsequent generation of secondary ^1^O_2_ (green dots) causes spreading of the catalase inactivation on the cell membrane of the initially triggered cells and their neighbours (**C**,**D**). Finally, the majority of cells in the population show substantial inactivation of their protective catalase and thus can foster intercellular apoptosis-inducing ROS signaling, but also can transmit bystander signaling to previously untreated cells, as shown in the next Figures.

**Figure 21:**
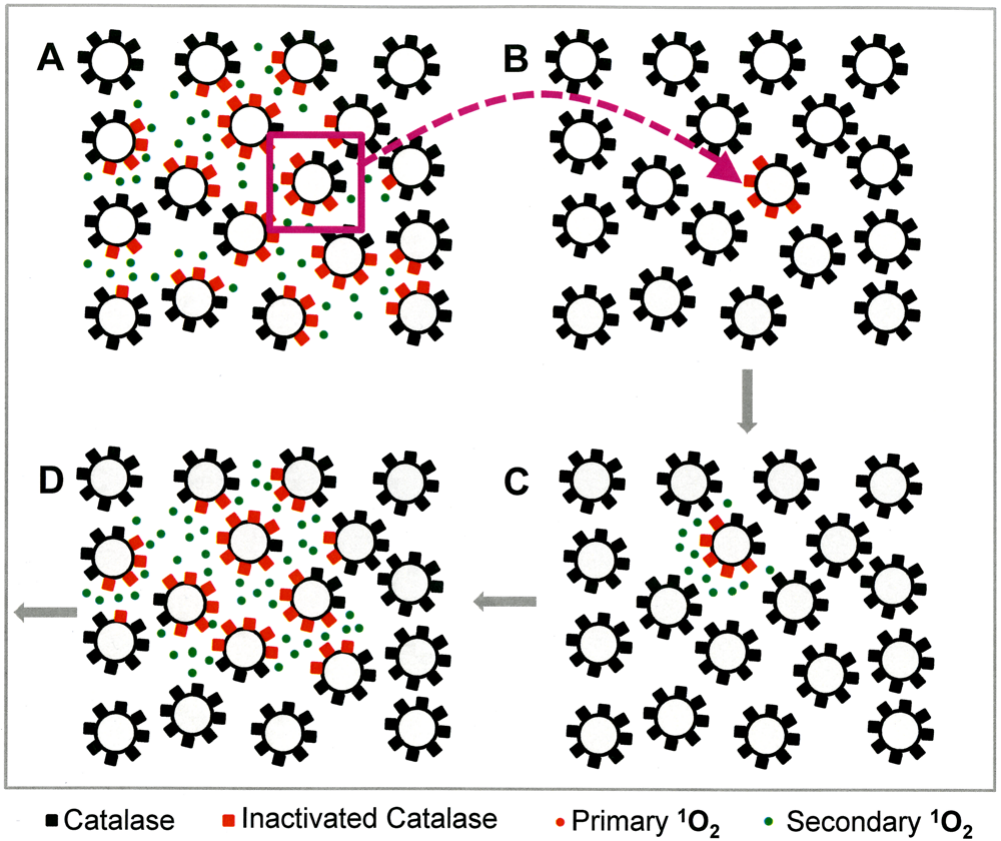
Propagation of bystander signaling. When a tumor cells have been pretreated with CAP or PAM, many of the cells in the population have acquired the potential to transmit bystander signaling mediated by secondary ^1^O_2_ generation. Therefore, the transfer of very few cells (symbolized by one cell with partially inactivated catalase here) has the potential to transmit bystander signaling to an untreated population (**B**). After short time at the right density, the majority of cells will have inactivated catalase, generate secondary singlet oxygen and will be ready to drive intercellular apoptosis induction.

**Figure 22:**
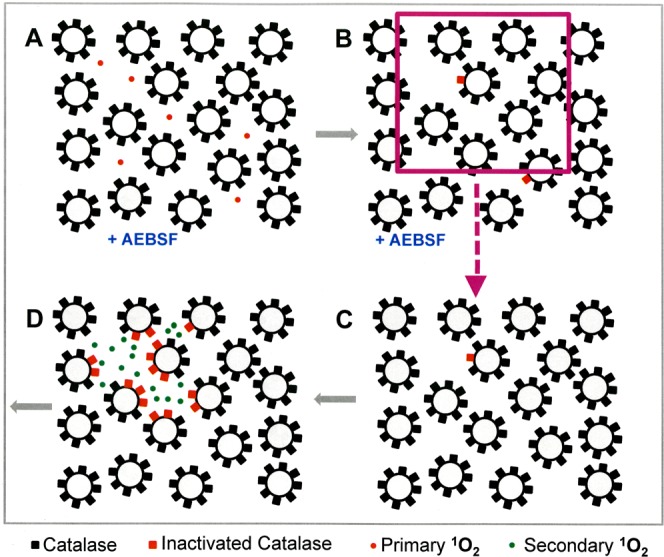
Bystander signaling is hampered through inhibition of NOX1. The presence of the NOX1 inhibitor AEBSF during CAP or PAM treatment prevents the generation of secondary ^1^O_2_. Therefore, only few cells will carry the “imprinted signature” of primary ^1^O_2_ action, i. e. a partially inactivated catalase population on their surface. To transmit bystander effect induction from this cell population to untreated cells requires to apply a much higher percentage of pretreated cells as under conditions without AEBSF. For this reason, the curves in the bystander experiments in this study show a strong right-ward shift in the presence of AEBSF during pretreatment and the kinetic analysis is characterized by a delay in apoptosis induction.

This starts presumably on the membrane of the cell that is triggered and is extended there, but definitely must also spread between cells (Please see Supplementary Fig. 23 for visualization). The basic prinicple of the biology of secondary ^1^O_2_ generation and its bystander effect-inducing potential is shown in Fig. 20. This scenario immediately indicates that transmission of few cells from a population that has undergone primary and secondary generation of ^1^O_2_ is sufficient to induce efficient bystander signaling in untreated tumor cells (Fig. 21). In contrast, a cell population in which secondary ^1^O_2_ generation was experimentally prevented through AEBSF-mediated inhibition of NOX1, the rare effect of primary ^1^O_2_ action leads to a constellation in which a very large proportion of the cell population has to be transfered to untreated cells, in order to have a chance to transmit bystander signaling (Fig. 22). This basis of quantitation of ^1^O_2_-medidated effects has been proven to be valid in our experiments and it has been extremely useful to verify the highly dynamic process of secondary ^1^O_2_ generation that follows the rare effect of primary ^1^O_2_.

The high efficiency of the process mediated by secondary ^1^O_2_ is based on the sustained NOX1- and NOS-driven generation of ONOO^−^ and H_2_O_2_ at the site of locally inactivated catalase. In contrast, primary ^1^O_2_ generation from long-lived CAP-derived species is limited by several restraints, as presented under Introduction.

Details of the analysis of the mechanisms of primary and secondary ^1^O_2_ generation are shown in Supplementary Figs 7–18.

^1^O_2_ not only inactivates catalase, but also SOD, as both enzymes carry histidine at their active center^76,77^. The parallel inactivation of membrane-associated catalase and SOD by ^1^O_2_ most likely causes a synergistic effect for apoptosis induction, as can be deduced from the synergistic effect that has been shown after parallel inhibition of membrane-associated catalase and SOD by neutralizing single domain antibodies^99^. Importantly, the effects due to ^1^O_2_-dependent inactivation of membrane-associated catalase and SOD are enhanced by ^1^O_2_-dependent activation of the FAS receptor (please find details in references #95 and #99).

It is not unlikely that ^1^O_2_ may react with other histidine-containing cellular components that are not related to RONS signaling. However, as the reaction of primary ^1^O_2_ with the cell membrane seems to be relatively rare effect, a nonspecific damaging effect through ^1^O_2_ seems to be either too weak to damage nonmalignant cells, or it can be repaired by the cells. This conclusion is derived from model experiments with a direct extracellular singlet oxygen source that do not show nonselective effects of ^1^O_2_ on nonmalignant cells^95^. In contrast, the rare interaction between ^1^O_2_ and the catalase on the surface of tumor cells induces a strong and selective effect, as it triggers an autoamplificatory system that is inherent specifically to malignant cells. Secondary ^1^O_2_ generated by CAP- or PAM-treated cells is, however, not sufficient to cause apoptosis induction directly, as seen from the inhibition of apoptosis induction by inhibitors that interfere with intercellular apoptosis-inducing RONS signaling, but not with ^1^O_2_ generation^60,^^63^.

It also needs to be considered that the concentration of the CAP-derived H_2_O_2_ is very rapidly decreasing through the consumption by membrane-associated catalase oftumor cells, as shown in Fig. 19. This finding is in good agreement with the results of other investigators^46,57,101^. It is also conceivable that the concentration of NO_2_^−^ is lowered through the oxidation to ^●^NO_2_ by catalase^98^. These catalase-mediated effects are thus establishing conditions where the generation of primary ^1^O_2_ is rapidly turning to suboptimal conditions. However, as the long lived species from CAP or PAM only have a triggering function in this biological system, at the time of their disappearance they have done their job and the targeted tumor cells have taken over.

Our data show that the induction of bystander effect-inducing signaling has the same biochemical characteristics as described for the inactivation of catalase in the preceding manuscript^63^, i. e. mediation by ^1^O_2_ and the role of ONOO^−^, O_2_^●−^, H_2_O_2_ as intermediates. The identity of these two processes is further indicated by the finding that siRNA-mediated knockdown of protective catalase^95^, inactivation of protective catalase by an exogenous ^1^O_2_ source^96^, as well as inactivation of catalase through modulation of the ^●^NO level^100^ allow for bystander signaling, in which ^1^O_2_ plays the central role. Furthermore, our preceding manuscript shows that conditions that allow induction of bystander effect-inducing potential, either by primary ^1^O_2_ alone or in combination with secondary singlet oxygen, are correlated with the inactivation of membrane-associated catalase.

In the preceding studies, catalase inactivation was detected and quantified by the challenge with ONOO^−^. This required that a substantial concentration of membrane-associated catalase has been inactivated. On the other hand, the analysis of bystander effect-inducing and receiving processes as studied here also allow to determine discrete inactivation of a few catalase molecules on the surface of tumor cells that would not be possible to detect by the challenge with ONOO^−^. These discrete levels of catalase inactivation have been termed “imprinted signatures” in this manuscript. They enable to establish the fullblown effect of catalase inactivation after the experimental conditions allow the imprinted signatures to be the starting point for the generation of secondary ^1^O_2_, driven by tumor cell-specific NOX1 and its NOS (see Supplementary Figs 17–22 for visualization). These findings show the dynamics of this biological system and offer a chance for profound analysis of the underlying biochemistry.

- The biochemical nature of the “imprint” is based on the local inactivation of few catalase molecules on the surface of tumor cells and the resultant “survival” of NOX1- and NOS-derived H_2_O_2_ and ONOO^−^ at that site. The activity of NOX1 and NOS thus determines the functionality of the imprint in subsequent ^1^O_2_-determined bystander signaling. It is important to recall that successful imprinting is absolutely dependent on the covalent attachment of the catalase molecules to the surface of the tumor cells, which allows the establishment of a site of protection of H_2_O_2_ and ONOO^−^ after inactivation of few catalase molecules. If protection of tumor cells towards extracellular H_2_O_2_ and ONOO^−^ would be achieved by soluble catalase, the inactivation of a few catalase could not establish an imprint, due to the compensation through mobile catalase molecules with their extreme turnover number of 16–44 000 substrate molecules per second^102^.

At present we see no chance to determine the autoamplificatory spreading of ^1^O_2_ generation and catalase inactivation *on individual cells* that have been triggered by primary ^1^O_2_ either from the gaseous phase of CAP or generated through interaction of the long-lived species in CAP treated medium.

However, there are no conceivable objections to the conclusion that the mechanism of bystander effect that is characterized in this manuscript, which causes spreading of ^1^O_2_ generation and catalase inactivation within a cell population, can be also applied to the process that hypothetically must occur on the originally targeted cell. This aspect is visualized in Supplementary Fig. 23.

The analysis shown in this manuscript confirms the highly dynamic effect, especially that of the secondary ^1^O_2,_ and offers a semiquantitative determination of its abundance compared to the primary ^1^O_2_. This finding confirms the driving role of the tumor cells for their own and selective cell death, after they have been triggered by the primary ^1^O_2_ oxygen from CAP/PAM. The dissection of the biological system also allowed to confirm that generation of the ^1^O_2_ from long-lived species of PAM and generation of the secondary ^1^O_2_ by the cells have a strong mechanistic overlap in the final part of the interactions, whereas the starting points are clearly different and can be relatively easily and clearly differentiated. All central findings of this manuscript have been evaluated with H_2_O_2_ and NO_2_^−^ as defined model compounds, using the analysis of bystander signaling as described here^60^. The results are congruent and thus strengthen the concept of CAP/PAM action as presented in this study. Furthermore, earlier studies^96^, utilizing an illuminated photosensitizer as a source for ^1^O_2_ are consistent with all conclusions presented in this and the preceding manuscripts^59,60^,^63^ on the same subject. Riethmüller *et al*.^96^ showed that extracellular ^1^O_2_ was not sufficient to kill tumor cells directly, but triggered autoamplification of secondary ^1^O_2_ through the interaction between tumor cell-derived H_2_O_2_ and ONOO^−^. The resulting substantial inactivation of the population of catalase molecules on the cell membrane was experimentally confirmed. Likewise, the consequence of the catalase inactivation, i. e. reactivated intercellular apoptosis-inducing ROS/RNS signaling was shown to be the essential trigger for the subsequent mitochondrial pathway of apoptosis. It was also shown that bystander signaling was the principle for autoamplification of the secondary ^1^O_2_ generation and catalase inactivation, in an experimental setting that was analogous to the approach used in this manuscript.

It is conceivable that the dynamics of this ^1^O_2 _-driven process is one of the explanations for the finding that CAP treatment of a tumor causes biological effects distant of the site of CAP application^6,16,17,103^, summarized in reference #94).

As cell death by chemotherapeutics, photodynamic therapy or radiation is often connected to subsequent immunological process that elicit a cytotoxic T cells response^104^; please find more details in Suppl. Materials), and as CAP treatment has been shown to trigger immunogenic cell death^105–111^, it may be speculated that CAP- and PAM-triggered, RONS-mediated apoptosis induction as described in this manuscript might be involved in triggering an immune response. This immunological process then might add an additional level of dynamics to the overall process of CAP and PAM-mediated antitumor effect, through intercalation of these two rather selective, highly efficient and dynamic processes. This interplay would ensure the efficiency and selectivity of CAP- and PAM-dependent tumor therapy.

This study and preceding work^59,60^,^63^ have shown that CAP-and PAM-mediated selective apoptosis induction in tumor cells depends on the interplay between a relatively rare initial triggering effect by primary CAP or PAM-derived singlet oxygen and a sustained, strong and selective response by the targeted tumor cells. It has also been shown that the long-lived species NO_2_^−^ and H_2_O_2_, derived from CAP and present in PAM are sufficient to establish the primary triggering effect. This opened the path for the understanding of selective RONS-mediated apoptosis induction in tumor cells by CAP and PAM. However, it would be naive to suggest that future tumor therapy should be based simply on the application of NO_2_^−^ and H_2_O_2_. Rather, it is adequate to use the frame of chemical biology established here for trying to understand, further develop and to use potential advantages of CAP and PAM application for several reasons.

Based on the comparison of the effects induced by PAM with those mediated by a combination of NO_2_^−^ and H_2_O_2_, Kurake *et al*.^33^ already concluded that PAM seems to contain additional, so far not defined inducers of tumor cell death. Though short-lived species derived from CAP are no longer existing in PAM, potential reaction products with lipids, or proteins modified by CAP at their thiol or tryptophan residues^112,113^ might inherit direct signaling functions or interact synergistically with the signaling pathway established by NO_2_^−^ and H_2_O_2_. A beneficial effect of these conceivable compounds on apoptosis induction and/or the quality of immunogenic cell death beyond the effects mediated by ﻿NO_2_^−^/H_2_O_2_ interaction seems not to be unlikely and therefore deserves further experimental analysis. The role of PAM for tumor therapy is particularly promising in cases where tumors or their metastases cannot be reached by surgery or CAP treatment. Therefore, any attempts to further optimize PAM application seem to be very desirable.

Theoretical considerations and initial findings^54^ allow to conclude that, whenever a tumor can be directly reached, CAP treatment might be advantageous compared to PAM treatment. CAP, in addition to its long-lived species, contains molecular species that may also cause direct inactivation of tumor cell catalase, such as singlet oxygen^76,77,95,96^ and ozone^114,115^. Furthermore, CAP-derived ozone (O_3_) might generate singlet oxygen after its reaction with amino acids^116,117^. The conceivable reaction of the short-lived CAP-derived species ^●^NO_2_ with CAP-derived superoxide anions or hydroperoxyl radicals may lead to the generation of peroxynitrate (O_2_NOO^−^) and peroxynitric acid (O_2_NOOH). These interaction may finally result in ^1^O_2_ generation through decomposition of peroxynitrate (O_2_NOO^−^)^95,118^. Furthermore, peroxynitrite (ONOO^−^) directly derived from CAP might enhance the formation of primary ^1^O_2_ generation through its interaction with H_2_O_2_, thereby avoiding the relatively slow formation of ONOO^−^ through the interaction between H_2_O_2_ and NO_2_^−^. It is predictable that the biological effects of these short-lived compounds from CAP will trigger the same mechanistic response of tumor cells as primary ^1^O_2_ derived from long-lived species, but may result in a kinetically determined advantage with respect to their antitumor effect.

It also seems to be very promising to further evaluate the effects of chlorine/chloride-related compounds in CAP, due to the connection between atomic oxygen and the formation and biological significance of hypochlorite, hypochlorous acid (OCl^−^/HOCl) as shown by Bekeschus *et al*.^119^, the multifaceted chemical biology of OCl^−^/HOCl and dichloride anion radicals (Cl_2_^●−^) as established by Wende *et al*.^38^, the potential of HOCl to interfere with catalase activity, as shown by Krych-Madej and Gebicka^120^ and the generation of ^●^OH through interaction between HOCl and tumor cell-derived O_2_^●−^ ^80,121^. The major aspects to be addressed for potentially successful application of short-lived CAP-derived species seems to be the relative increase in their concentration, as well as development of regimes of application that are less affected by the short-lived nature of the species discussed above, and that allow a close contact between the plasma source and the tumor.

It is an interesting task to discuss several important recent findings by Keidar’s group in the light of the model on CAP and PAM action on tumor cells, as presented in this manuscript. The findings by Yan *et al*.^46,57,101^ on consumption of H_2_O_2_ by tumor cells can be explained by the activity of membrane-associated catalase on tumor cells. It is in perfect agreement with the findings presented in Fig. 19 of this manuscript. This phenomenon seems to describe an essential process that is counteracting the biological effect of PAM. The strength of this effect needs to be taken into consideration when PAM effects are optimized. The findings by Yan *et al*.^101^ on a reciprocal correlation between cytotoxicity of CAP on tumor cells and the extracellular H_2_O_2_-scavenging rate of the tumor cells is also rationally explained by the presence of catalase on the surface of tumor cells, according to our model.

The strong cell-based H_2_O_2_ generation by CAP, but not by PAM^54^ can be explained by our model, under the premise that the plasma source used in their experiments generated substantial concentrations of ^1^O_2_ or precursors of ^1^O_2_. In the experiments performed by Yan *et al*.^54^, the cells were accessible to direct plasma treatment, due to a low volume of surrounding medium. Therefore, inactivation of membrane-associated catalase directly by ^1^O_2_ from the gaseous phase of CAP might have been sufficient to cause a net increase in cell-derived H_2_O_2_ that was not decomposed at the sites of inactivated catalase. Compared to this obviously massive effect, the relatively slow and time-requiring effect of ^1^O_2_ generated from the long-lived species H_2_O_2_ and NO_2_^−^ seemed to be suboptimal to give a comparable rise in free H_2_O_2_ within very short time, compared to the directly acting ^1^O_2_ from CAP. These findings are in good agreement with our model, as are the findings that under these conditions, cell death induced by direct CAP treatment is faster and more effective than cell death induced by PAM. The apparent delay of action is explained by the requirement for several rounds of autoamplification of catalase inactivation, in contrast to the immediate inactivation of catalase by CAP-derived ^1^O_2_. This scenario seems to represent a conclusive example for benefitial effects of CAP compared to PAM, dependent on the relative direct accessibility of the target cells. It should be relatively easy to verify or falsify the connection between these important findings and our model. If the conclusions drawn in this discussion are correct, the strong H_2_O_2_ generation after direct CAP treatment should be prevented a) by the singlet oxygen scavenger histidine during treatment, b) by inhibition of NOX1 of the tumor cells by AEBSF or c) through siRNA-based knockdown of NOX1. Based on the kinetics of the process, histidine should only be inhibitory, when applied before the beginning of the treatment and without effect even if it is added a few seconds after beginning of the treatment. A challenge with exogenous ONOO^−^ immediately after CAP treatment would clarify wether membrane-associated catalase was indeed the primary target of CAP^59,81^.

The activated state of tumor cells after direct CAP treatment^122^ might be related to the same chemical biology and reflect a state a partial inactivation of membrane-associated catalase. The reported desensitization from this step fits perfectly to findings on the dynamic control of catalase expression by free H_2_O_2_^81^. This conclusions might also be verified or falsified by monitoring the activity of membrane-associated catalase by ONOO^−^ challenge experiments and by modulation of NOX1 activity. Independent of underlying mechanisms, these findings point to the chances of direct CAP treatment.

The study of CAP-derived molecular species that trigger or enhance immunogenic cell death is a progressing field of experimentation. It will be important to find out whether CAP-derived RONS are also directly involved in the induction of the release of damage-associated molecular patterns (DAMPs) or whether these are a consequence of RONS-mediated apoptosis induction (or both).

The combination of active NOX1 and membrane-associated catalase on the membrane of tumor cells combines a hallmark of transformation (NOX1)^123^, reviewed in references # ^124,125^, with the hallmark of successful tumor progression, i. e. membrane-associated catalase^74,81,126,127^. Therefore, this redox-related system on the surface of tumor cells represents a unique target for antitumor strategies. As shown in this report, CAP- and PAM-derived RONS act as very direct, effective and selective trigger to induce this specific redox control system to generate a sustained RONS-based response that utilizes bystander signaling and culminates in selective apoptotic cell death. Other modes of targeting this redox system have been discussed as well^100,124,125,128^. These are modulators of NO metabolism (such as arginine, interferons, inhibitors of NO dioxygenase like anthocyanidins/flavonoids/azoles)^93,100,129^, extracellular singlet oxygen generators^96^ and neutralizing single domain antibodies directed towards catalase or SOD^99^.

SiRNA-mediated knockdown of catalase or cell-permeable inhibitors of catalase seem to be less promising, as they do not provide selective inhibition of *membrane-associated* catalase of tumor cells. Inhibition of intracellular catalase might cause unwanted side effects for nonmalignant cells.

What might be the possible merits to use CAP- and PAM-based tumor therapy in the future? The answer to this question comprises seven strong points: 1) the direct confrontation of the redox system of tumor cells with CAP- and PAM-derived RONS, with 2) its inherent chance to successfully analyze these interactions and establish a rational regime for therapy; 3) the high selectivity of action at the right dosage; 4) the recognizable connection to immunogenic cell death; 5) the low side effects due to low doses of RONS that are initially required; 6) the chance to use the established knowledge on RONS signaling for the establishment of synergistic interactions (e. g. with other therapeutics that also target redox elements) and 7) the predictable favorable costs of the therapy, with its obvious positive implications for the individual and the community.

### The relationship of this study to previous studies of the authors

D.B. Graves and G. Bauer first cooperated in a primarily theoretical and deductive approach, in which established knowledge on the composition of CAP and PAM was connected to published results on the differential effects of defined RONS on nonmalignant and malignant cells. This work allowed to propose a model that was focused on the role of singlet oxygen for selective apoptosis induction in tumor cells^95^. The theoretical approach based on Bauer and Graves^95^ was further developed by connecting it to the concepts of immunogenic cell death and the dominant control function of aquaporins^118^. In parallel, a theoretical approach allowed to establish a model for the generation of ^1^O_2_ through the interaction between H_2_O_2_ and NO_2_^−^ ^130^ and for potential synergistic effects during H_2_O_2_/NO_2_^−^ interaction^131^. This model has been confirmed in a bottom-up experimental approach, utilizing defined concentrations of H_2_O_2_ and NO_2_^−^. These data have been presented at ICPM-7 in Philadelphia and have been recently published^59,60^. Based on these theoretical and experimental preparations, we were ready for an inductive experimental approach for the elucidation of the potential mechanism of selective apoptosis induction in tumor cells by CAP and PAM, using a plasma source developed in the group of Z. Machala (Bratislava). The results of the cooperation between the four authors of this study are presented in this manuscript (with the focus on the dynamics of the process) and a parallel manuscript^63^ (with the focus on the basic mechanism of CAP and PAM action).

## Supplementary information

supplementary informations

## Data Availability

All data generated or analyzed during this study are included in this published article (and its Supplementary Information Files).
