# Supplementary material for: Dynamics of Singlet Oxygen-Triggered, RONS-Based Apoptosis Induction after Treatment of Tumor Cells with Cold Atmospheric Plasma or Plasma-Activated Medium
Source: Sci Rep. 2019 Sep 26;9:13931. doi: 10.1038/s41598-019-50329-3 (PMC6763425; doi:10.1038/s41598-019-50329-3)
Supplement: Supplementary file 1 — supplementary informations [file 41598_2019_50329_MOESM1_ESM.pdf]

Supplementary Materials

Dynamics of singlet oxygen-mediated catalase inactivation after treatment of tumor cells with Cold Atmospheric Plasma or Plasma Activated Medium

Georg Bauer, Dominika Sersenová, David B Graves and Zdenko Machala

Guide through Supplementary Materials:

The Supplementary Materials provide additional experimental results (**"Supplementary Results"**) that complement the results shown in the main part of this study. They are intended to provide additional information about our experimental system.

The "Supplementary Discussion" illustrates principles and details of the complex biological and biochemical aspects of this study in a way that was not possible in the main part of the manuscript due to the length and complexity (B.1-4). B.5 then discusses the extended flowchart of CAP/PAM-mediated apoptosis induction in tumor cells in the light of the dynamic processes studied here and in relationship to other models of CAP/PAM action.

Contents:

A. Supplementary Results

A.1 Kinetic analysis of the biological effects of primary singlet oxygen ($^1O_2$) derived directly from CAP.

A.2 Quantitation of the effect of secondary $^1O_2$
A.3 Quantitation of the effect of primary $^1O_2$ generated by PAM
A.4 Bystander signaling effects by primary and secondary $^1O_2$

B. Supplementary Discussion:

B.1 The significance of inhibitor studies for the elucidation of the mechanism of the generation of primary and secondary $^1O_2$
B.2 Visualization of the dynamic processes triggered by primary and secondary singlet oxygen ($^1O_2$)
B.3 Dissection of the effects of primary $^1O_2$ (derived either from the gaseous phase of CAP or generated through the reaction between H_2O_2 and NO_2^-) and secondary $^1O_2$, generated by the tumor cells.
B.4 Autoamplification of secondary $^1O_2$ *generation on the initially triggered tumor cell and adjacent cells*
B.5 Detailed flow chart of CAP/PAM-mediated apoptosis induction in tumor cells
B. 6 Comparison to other models for CAP and PAM-mediated apoptosis induction
B. 7 The potential connection between CAP-and PAM-mediated apoptosis induction and specific T cell activation

A. Supplementary Results

A.1 Kinetic analysis of the biological effects of primary singlet oxygen ($^1O_2$) derived directly from CAP.

CAP treatment of tumor cells in the presence of the NOX1 inhibitor AEBSF and the $ONOO^-$ decomposition catalyst FeTPPS allows to focus on the effects induced by the primary $^1O_2$ derived directly from the gaseous phase of CAP. FeTPPS prevents the generation of primary $^1O_2$ derived from NO_2^-/H_2O_2 interaction, and AEBSF and FeTPPS prevent the generation of secondary $^1O_2$ by the tumor cells.

When MKN-45 gastric carcinoma cells were treated with CAP for 1 min in the presence of the NOX1 inhibitor AEBSF and the $ONOO^-$ decomposition catalyst FeTPPS, followed by a washing step and further incubation in fresh medium,

apoptosis induction started 4 h after CAP treatment (Supplementary Figure 1 A). The presence of the $^1O_2$ scavenger histidine during CAP treatment or after the washing step completely prevented apoptosis induction. As AEBSF and FeTPPS prevented the generation of secondary $^1O_2$, and as FeTPPS in addition counteracted potentially occurring primary $^1O_2$ generation through NO_2^-/H_2O_2 interaction with $ONOO^-$ as intermediate (see Supplementary Figure 14 for details), the triggering $^1O_2$ in this experiment must have derived directly from the gaseous phase of CAP. $^1O_2$ that seemed to be required after the washing step is indicative for the generation and action of secondary $^1O_2$ by the tumor cells themselves. The generation of secondary $^1O_2$ and its action seemed to be nearly completed after 1 h, as addition of histidine at 1 h only had a minor inhibitory effect (Supplementary Figure 1 B). Addition of histidine 4 h after CAP treatment even had a slight stimulatory effect on apoptosis induction which can be explained by prevention of excess catalase inactivation that can lead to supraoptimal inhibition of HOCl signaling by H_2O_2 (Bechtel and Bauer, 2009, b). The inhibitory effect of FeTPPS added at different time points after the washing step showed the same profile as that of histidine (Supplementary Figure 1 C), which is consistent with the conclusion that $ONOO^-$ acts as intermediate during secondary $^1O_2$ generation.

A repeat experiment in which CAP treatment for 1 min was carried out in the presence of AEBSF, with FeTPPS substituted by the catalase mimetic EUK-134 (Supplementary Figure 2). It allowed the same conclusions as the experiment described in Supplementary Figure 1. This is consistent with the preventive effect of the catalase mimetic EUK-134 on the primary $^1O_2$ generation through NO_2^-/H_2O_2 interaction.

Supplementary Figure 1

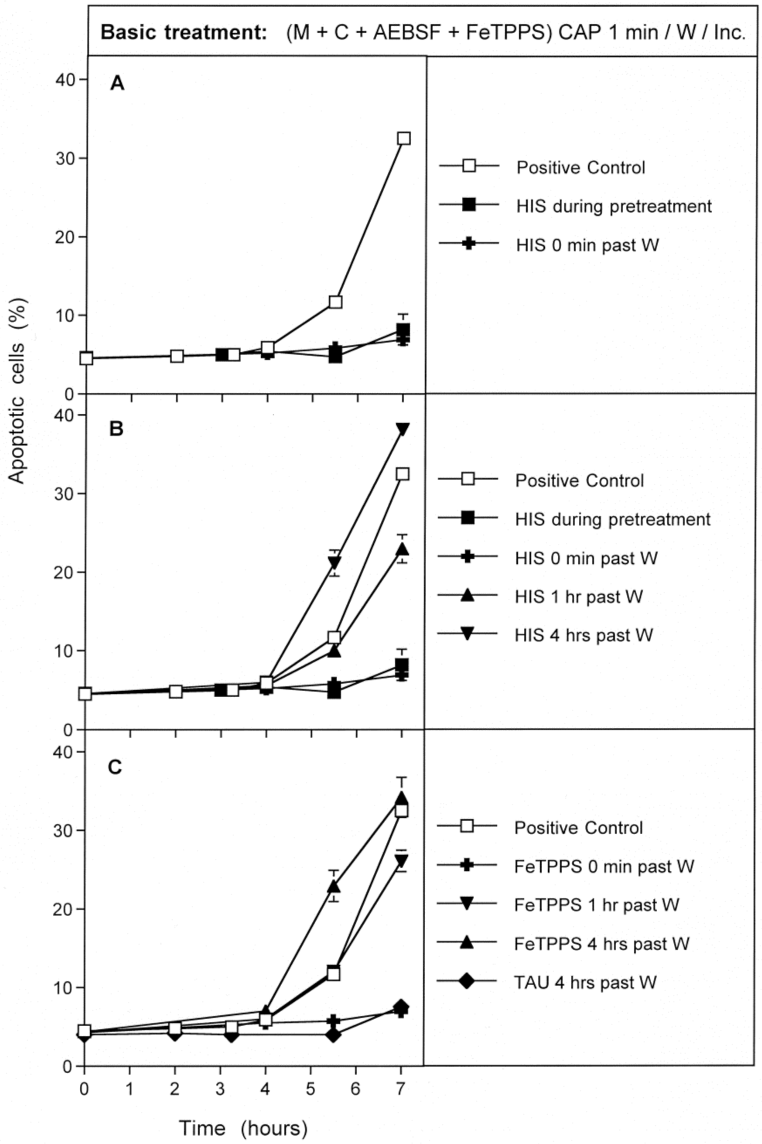

Supplementary Figure 1. The role of $^1O_2$ and $ONOO^-$ for apoptosis induction in tumor cells by the primary $^1O_2$ derived from the gaseous phase of CAP.

Cells (“C”) in medium (“M”) were treated with CAP for 1 min in the presence of 100 µM AEBSF and 25 µM FeTPPS. Immediately after CAP treatment, the assays were washed three time and further incubated (“Inc”). Inhibitors were added at the indicated steps. The results show that apoptosis induction in this regime is dependent on $^1O_2$ both before and after the washing step (A). They also show that the $^1O_2$-dependent step after washing (conceivably by secondary $^1O_2$) requires time (B). The same is true for the $ONOO^-$ -dependent step after washing (C). The strong inhibitory effect of taurine even four hours post washing shows the role of HOCl signaling for apoptosis-inducing signaling late in this process.

Statistical analysis: A: Apoptosis induction at 5.5 h and later was highly significant ($p<0.001$). Inhibition by histidine during pretreatment and after the washing step was highly significant ($p<0.001$). B, C: Apoptosis induction at 5.5 h and later, as well as inhibition by histidine added at 0 min past washing and by taurine was highly significant ($p<0.001$).

Supplementary Figure 2

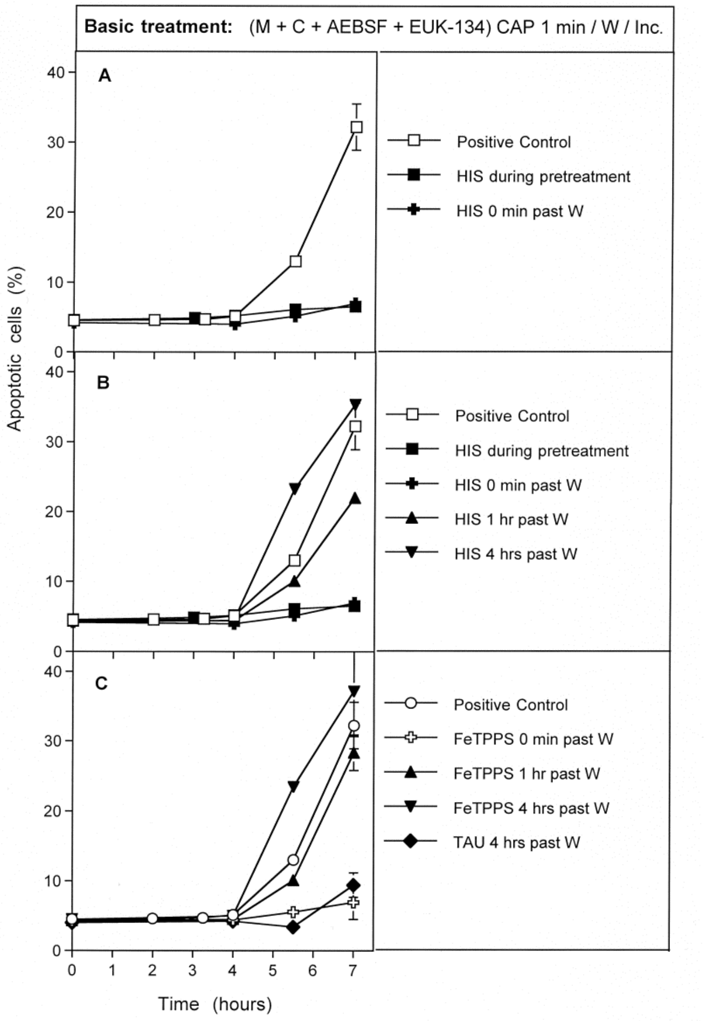

Supplementary Figure 2. The role of $^1O_2$ and $ONOO^-$ for apoptosis induction in tumor cells by $^1O_2$ derived from the gaseous phase of CAP.
The experiment was performed as described in Supplementary Figure 1, with the modification that treatment with CAP was in the presence of the catalase mimetic EUK-134 instead of FeTPPS.
Statistical analysis: A: Apoptosis induction at 5.5 h and later was highly significant (p<0.001). Inhibition by histidine during pretreatment and after the washing step was highly significant (p<0.001). B, C: Apoptosis induction at 5.5 h and later, as well as inhibition by histidine added at 0 min past washing and by taurine was highly significant (p<0.001).

When CAP treatment in the presence of either AEBSF plus FeTPPS or AEBSF plus EUK-134 was performed for 1 min or 5 min, and then the cells were washed and further incubated in fresh medium, cells that had been treated for 5 min started with

apoptosis induction two hours earlier than the cells that had been treated for 1 min (Supplementary Figure 3). Control experiments in which histidine or FeTPPS were

Supplementary Figure 3

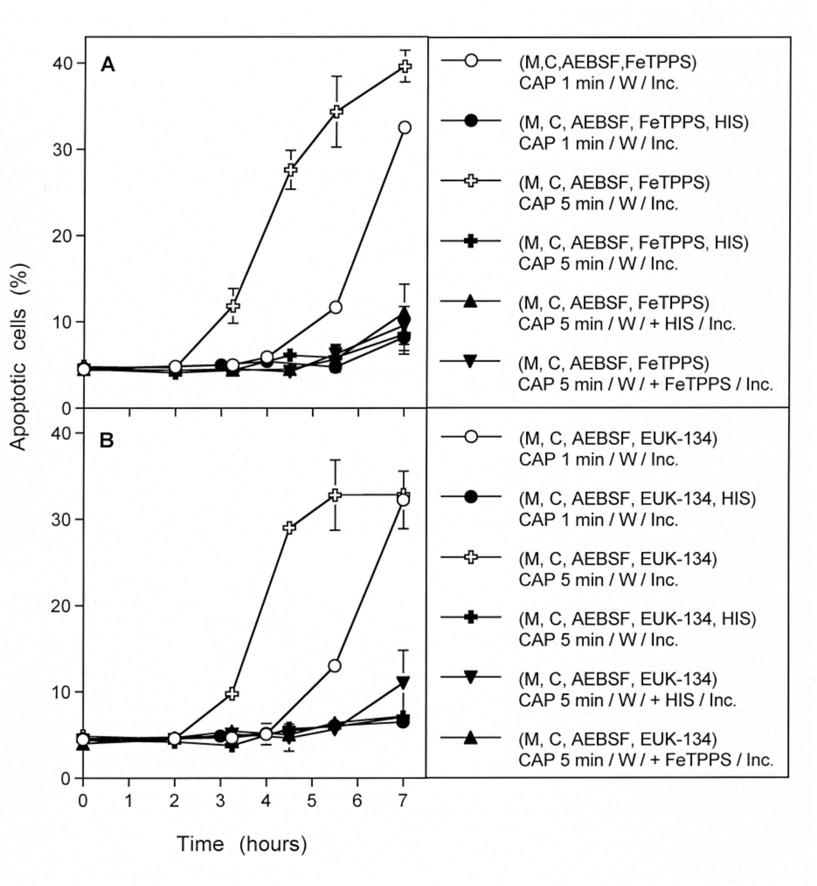

Supplementary Figure 3. This figure analyses the kinetics of tumor cells that had been pretreated with CAP for 1 or 5 min under conditions that prevented primary $^1O_2$ generation from long-lived species from CAP, and also inhibited the secondary $^1O_2$ generation, i. e. in the presence of FeTPPS or alternatively EUK-134, and AEBSF. The data show the profound effect of treating time on the starting point of the kinetics of apoptosis induction.
Statistical analysis: Apoptosis induction defined by open crosses (3 h and later) and by open circles (5.5 h and later), as well as inhibition by all inhibitors was highly significant (p<0.001).

added to cells that had been treated with CAP for 5 min and then had been washed, showed that apoptosis induction after the washing step was dependent on secondary $^1O_2$ with $ONOO^-$ as an essential intermediate for its generation. This finding demonstrates that the length of treatment with primary $^1O_2$ directly derived from CAP determined the onset of apoptosis. *This is consistent with the concept of bystander signaling shown in Figure 15, as more hits by primary $^1O_2$ should increase the*

number of starting points for bystander signaling and thus have a direct impact on the onset of apoptosis induction.

A.2 Quantitation of the effect of secondary $^1O_2$

The effect of secondary $^1O_2$ was determined in experiments that utilized the potential of CAP-treated cells to trigger a bystander-like effect in untreated cells. The percentage of pretreated cells that are required to lead to apoptosis induction in an untreated population allow us to estimate the spreading of secondary $^1O_2$ in the population.

Treatment of tumor cells with CAP for 1 min, immediately followed by a washing step was not sufficient to allow these cells to trigger apoptosis induction 5.5 h after mixing to untreated cells (Supplementary Figure 4 A). However, when the CAP-treated cells were incubated in the same medium for 25 min before they were washed and added to untreated cells, a remarkable bystander signaling-inducing effect was observed. As little as 0.4 % of treated cells within the population (i. e. 50 treated cells per 12,500 cells in total) allowed for nearly maximal apoptosis induction. This result indicates that during the 25 min incubation after CAP treatment, $^1O_2$ derived from the long-lived species in plasma-treated medium and the resultant secondary $^1O_2$ generated by the triggered tumor cells must have imprinted the potential to induce bystander signaling in a large number of cells. As selective inhibition of secondary $^1O_2$ generation by the NOX1 inhibitor AEBSF caused drastically reduced bystander signaling (reflected in the need to transfer large numbers of treated cells to untreated cells for triggering apoptosis induction), secondary $^1O_2$ generated by triggered tumor cells seemed to contribute the most to this effect, whereas the effect of primary $^1O_2$ seemed to play a minor role only. In the presence of histidine, FeTPPS or EUK-134 during CAP treatment and initial 25 min incubation, no apoptosis induction was

observed at all volumes of treated cells transferred. This finding is consistent with the inhibition of both primary and secondary ${}^1O_2$ generation,

as both processes require H_2O_2 and $ONOO^-$ as intermediates. The effect of

Supplementary Figure 4

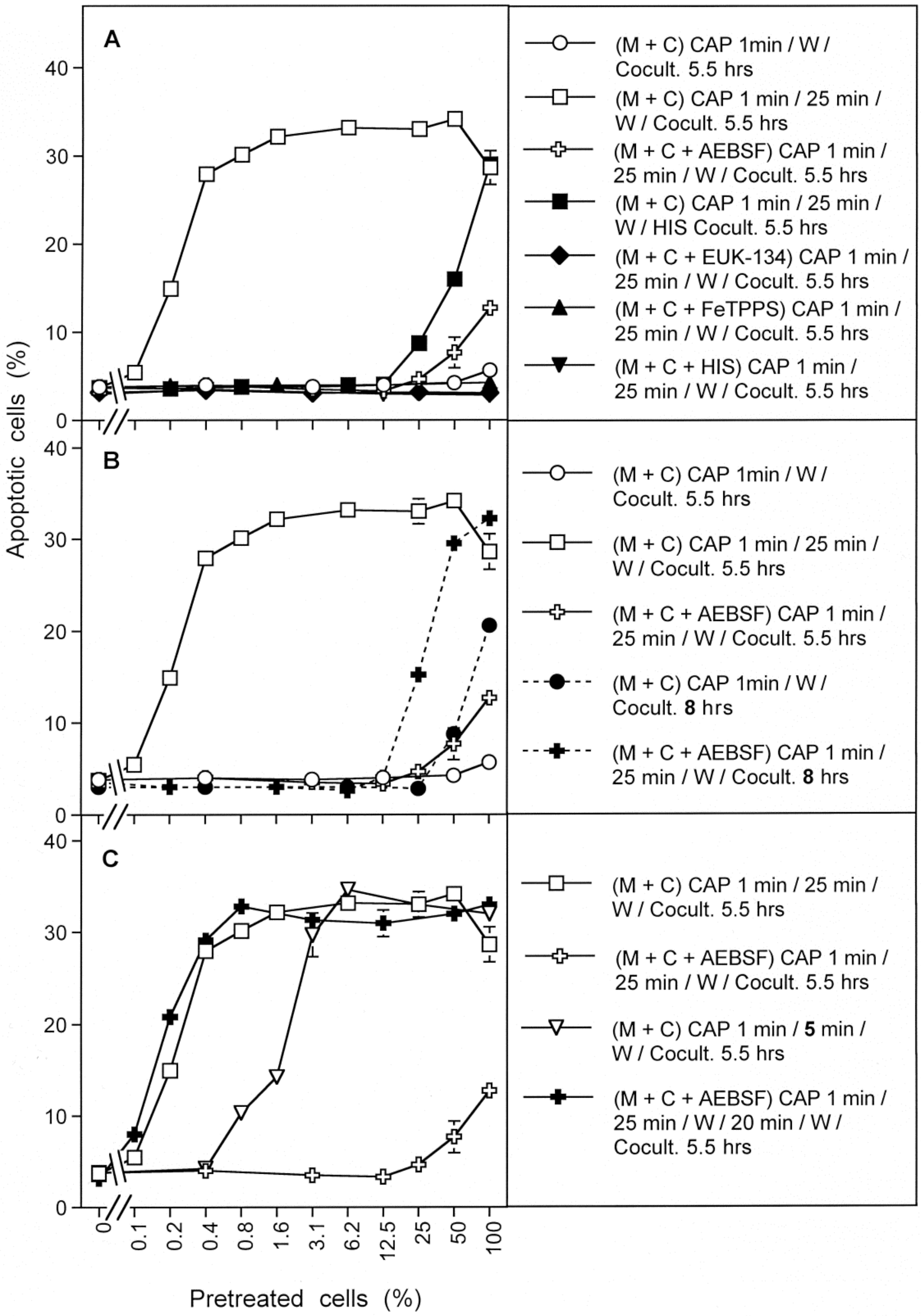

Supplementary Figure 4. Analysis and quantitation of bystander effect induction by CAP. Experimental details and conclusions are in the text.

Statistical analysis: A, B: Open squares: apoptosis induction by 0.2 % pretreated cells and higher, as well as the effects by all inhibitors are highly significant (p < 0.001). C: Apoptosis induction is highly significant for 0.2 % pretreated cells (open squares and closed crosses), 1.6 % pretreated cells (open triangles) and 100 % pretreated cells (open crosses) (p<0.001). The shift between the curves characterized by open squares and open triangles, open and closed crosses, as well as open squares and open crosses are highly significant (p<0.001).

increasing the coculture time on the kinetics of apoptosis induction in this experiment is shown in Supplementary Figure 4 B. Supplementary Figure 4 C shows that reduction of the incubation time from 25 min to 5 min immediately after CAP treatment causes the expected decrease in the bystander signaling effect.

A.3 Quantitation of the effect of the primary $^1O_2$ generated by PAM

When PAM was generated by the treatment of medium with CAP for 1 min or for 3 min, subsequent treatment of tumor cells with PAM in the presence of AEBSF (preventing secondary $^1O_2$ generation) showed that the increased concentration of long-lived species in PAM generated by 3 min CAP treatment caused a strong increase in the bystander effect inducing potential of the cells (Supplementary Figure 5).

Supplementary Figure 5

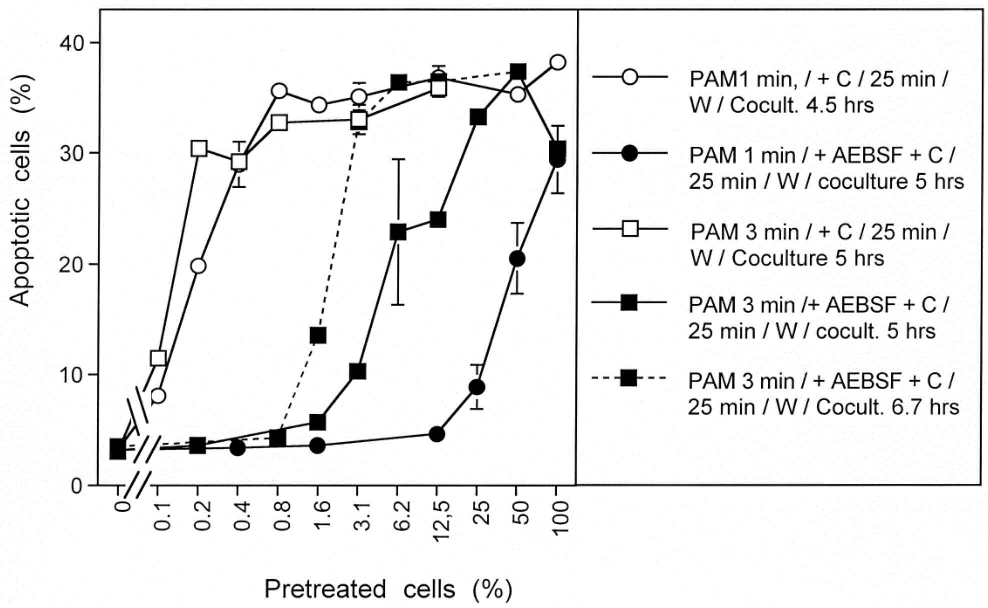

Supplementary Figure 5. An increase in the treatment time with CAP during the generation of PAM increases the bystander effect inducing potential of PAM. Details are in the legend and in the text.

Statistical analysis: Apoptosis induction is highly significant for open circles and open squares at 0.2 % pretreated cells, closes squares (dashed line) at 1.6 % pretreated cells, open squares (solid line) at 6.2 % pretreated cells and closed circles at 50 % pretreated cells. The shift between the curves are highly significant, except for the difference between open squares and open circles.

A.4 Bystander signaling effects by primary and secondary $^1O_2$

CAP treatment for 10 min, followed by a washing step and transfer of cell dilutions to untreated cells caused a massive bystander effect that is explained by the signature of primary, as well as secondary $^1O_2$ on the cells (Supplementary Figure 6). When the generation of secondary $^1O_2$ was prevented through the NOX1 inhibitor during CAP treatment, the bystander inducing signaling was reduced, which was reflected in rightward shift of the curve. This shift is due to prevention of generation of secondary $^1O_2$ during CAP treatment. When the generation of secondary $^1O_2$ was prevented

Supplementary Figure 6

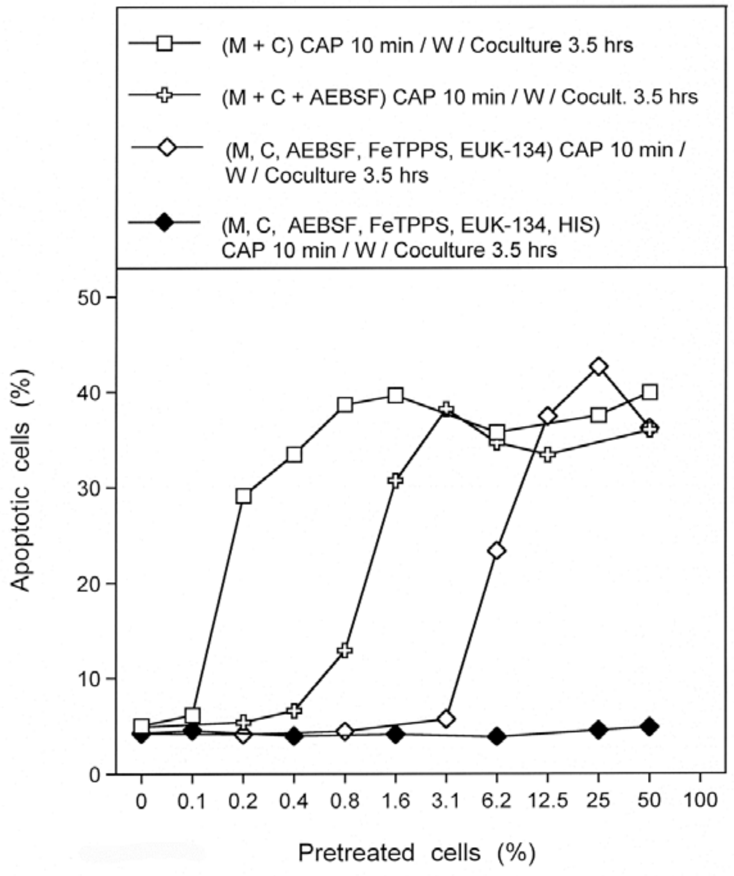

Supplementary Figure 6. Quantitation of the bystander inducing effects of primary $^1O_2$ from CAP or long-lived species from PAM, in combination with secondary $^1O_2$ generated by the cells. Please find details of the experiment and conclusions in the text.

Statistical analysis: Apoptosis induction is highly significant ($p<0.001$) for open squares at 0.2 % pretreated cells, open crosses at 0.8 % preatreated cells and open diamonds at 6.2 % pretreated cells. The shift between the curves is highly significant ($p<0.001$).

during CAP treatment through inhibition of NOX1 by AEBSF, and in parallel the generation of primary $^1O_2$ through the interaction between H_2O_2 and NO_2^- was prevented through addition of FeTPPS and EUK-134 that decompose essential intermediates, the rightward shift of the curve was further extended. The curve is indicative for the induction of bystander effect inducing potential seems to be caused by $^1O_2$ that is directly contributed by CAP, without supplementation by primary $^1O_2$ generated by long-lived species H_2O_2/ NO_2^- and without enhancement by secondary $^1O_2$ generated by the tumor cells themselves.

B. Supplementary Discussion:

B. 1 The significance of inhibitor studies for the elucidation of the mechanism of the generation of the primary and secondary $^1O_2$

Supplementary Figure 7 (corresponding to Figure 1 in the main manuscript) gives an overview of the mechanism of primary and secondary $^1O_2$ generation after CAP and PAM action, as well as on the role of inhibitors in this scenario.

Due to a strong overlap between the mechanism of the primary and secondary $^1O_2$ generation, inhibitors like histidine, $ONOO^-$, EUK-134 and mannitol cause a strong inhibitory effect in both steps. In contrast, inhibition of NOX1 by AEBSF, NOS by L-NAME and inhibition of caspase-8 selectively and efficiently prevents the generation of the secondary $^1O_2$.

Supplementary Figure 7

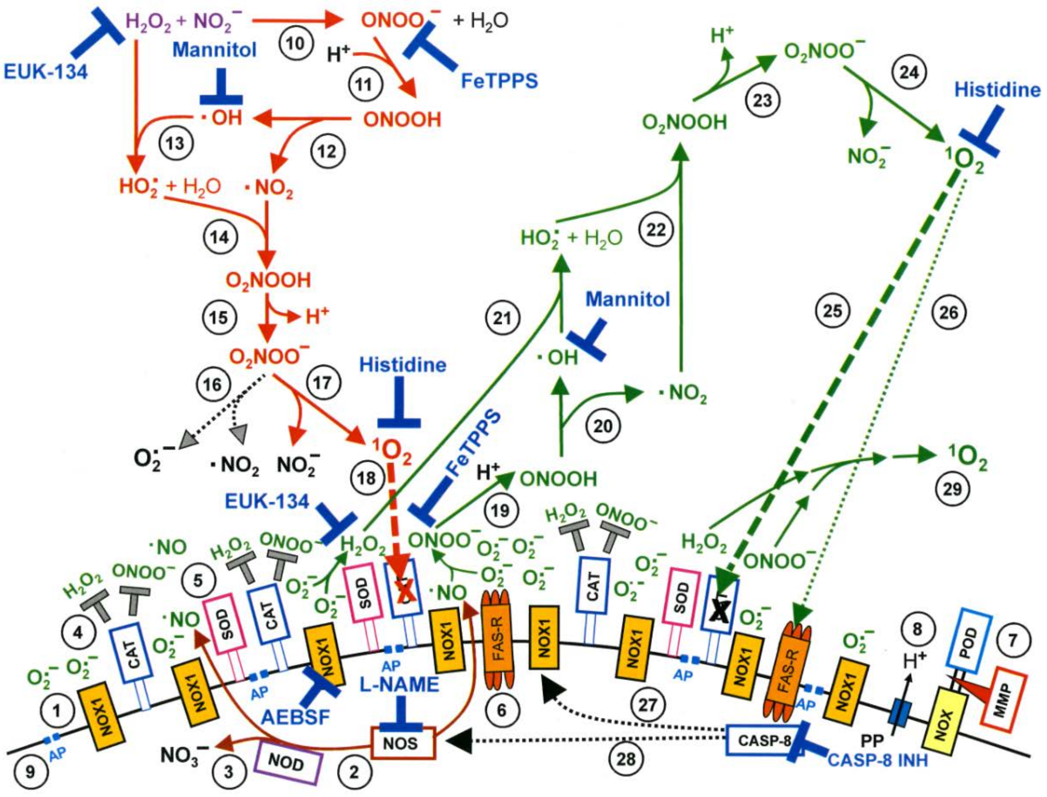

Supplementary Figure 7. Generation of primary $^1O_2$ from H_2O_2 and NO_2^- derived from CAP/PAM (red) and the subsequent generation of secondary $^1O_2$ (green).

NADPH oxidase 1 (NOX1) is expressed in the membrane of tumor cells and generates extracellular superoxide anions ($O_2^{\bullet -}$) (#1). NO synthase (NOS) (#2) generates $^\bullet$NO which can be either oxidized by NO dioxygenase (NOD) (#3) or pass through the cell membrane. Membrane-associated catalase (#4) protects tumor cells towards intercellular RONS-mediated signaling. Comodulatory SOD (#5) is required to prevent $O_2^{\bullet -}$-mediated inhibition of catalase. Further important elements in the membrane are the FAS receptor (#6), Dual oxidase (DUOX) (#7), from which a peroxidase domain (POD) is split through matrix metalloprotease, proton pumps (#8) and aquaporins (#9).

H_2O_2 and NO_2^- derived from CAP treatment and stable in PAM interact and generate peroxynitrite ($ONOO^-$) (#10). In the vicinity to membrane-associated proton pumps $ONOO^-$ is protonated to peroxynitrous acid (ONOOH) (#11) and decomposes into $^\bullet NO_2$ and $^\bullet$OH radicals (#12). $^\bullet$OH radicals react with H_2O_2, resulting in the formation of hydroyperoxyl radicals ($HO_2^\bullet$) (#13). The subsequent generation of peroxynitric acid (O_2NOOH) (#14) and peroxynitrate (O_2NOO^-) (#15) allows for the generation of “primary singlet oxygen” ($^1O_2$) (#17). Primary $^1O_2$ causes local inactivation of membrane-associated catalase (#18). Surviving H_2O_2 and $ONOO^-$ at the site of inactivated catalase are the source for sustained generation of “secondary $^1O_2$” through reactions # 19- #24. Secondary $^1O_2$ may either inactivate further catalase molecules (#25) and thus trigger autoamplification of $^1O_2$ generation (#29), or activate the FAS receptor (# 26) and in this way enhance the activities of NOX1 and NOS. This enhances the efficiency of secondary $^1O_2$ generation. The site of action of specific inhibitors and

scavengers are indicated. (This figure is identical to Figure 1 in the main text. It is shown here again for convenience, as it is required for comparison with the subsequent Supplementary Figures 8-16.)

Scavenging of primary $^1O_2$ by histidine is sufficient to block catalase inactivation by primary $^1O_2$ and therefore prevents the generation of secondary $^1O_2$ (Supplementary Figure 8). Likewise, the individual action of either EUK-134, mannitol or FeTPPS efficiently prevents the generation of primary $^1O_2$ (Supplementary Figure 9) and therefore also prevents generation of secondary $^1O_2$.

Supplementary Figure 8

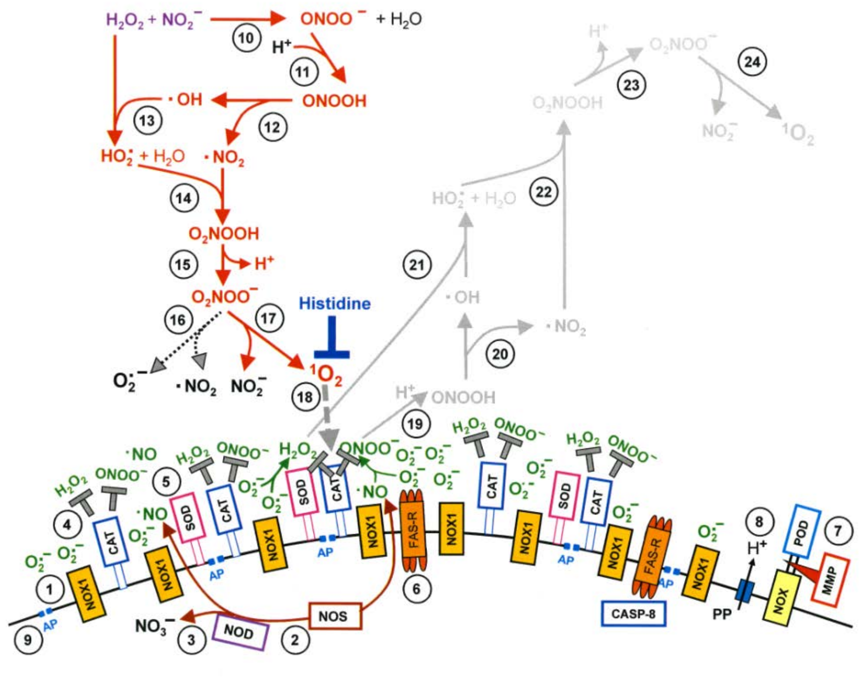

Please find the Legend to Supplementary Figure 8 and 9 on the next page.

Supplementary Figure 9

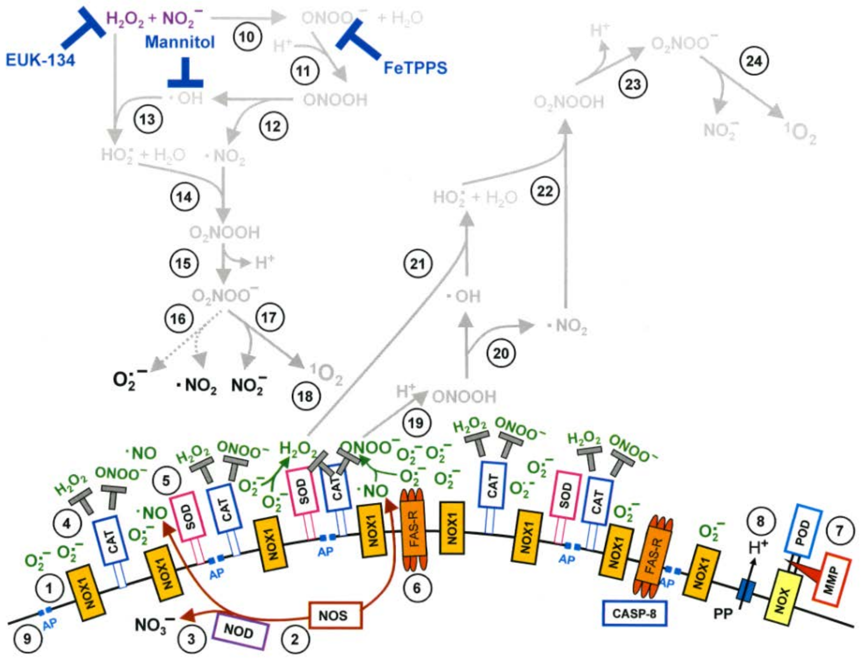

Legend to Supplementary Figures 8 and 9
These two figures are based on the preceding Supplementary Figure 7. They show the effect of the singlet oxygen scavenger histidine (HIS) (Supplementary Figure 8) or the catalase mimetic EUK-134, the ONOO decomposition catalyst FeTPPS or the •OH radical scavenger mannitol (MANN) (Supplementary Figure 9) on the generation and action of primary $^1O_2$.

Supplementary Figure 10

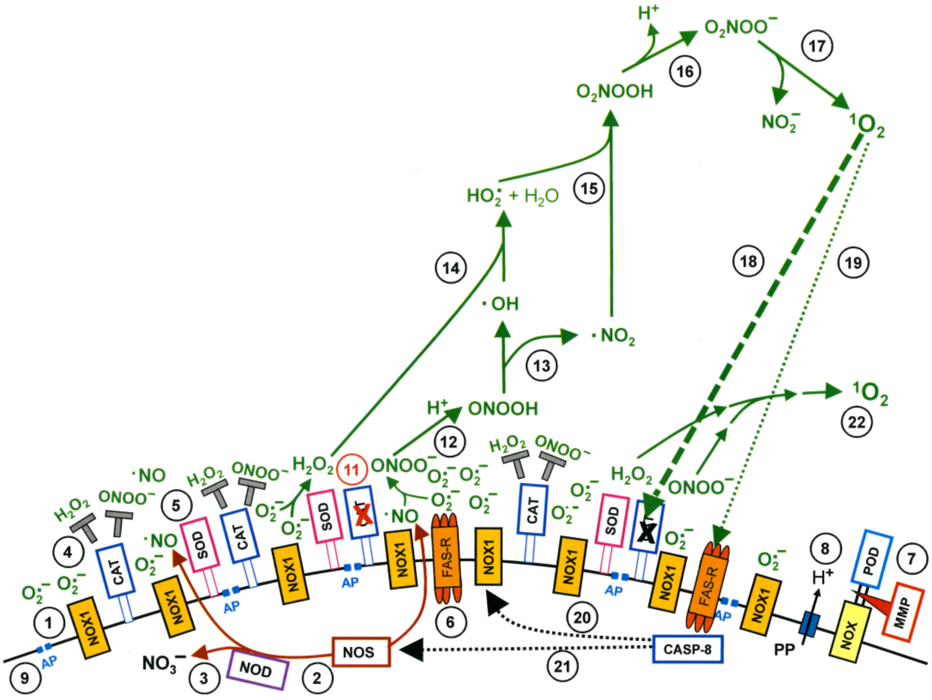

Legend to Suppl. Figure 10: Local inactivation of catalase by primary $^1O_2$ (#11) allows subsequent generation of secondary $^1O_2$ through reactions #12- #17. Secondary $^1O_2$ then starts autoamplification of $^1O_2$ generation through catalase inactivation (#18) or activates the FAS receptor (#19), which enhances the activities of NOX1 (#20) and NOS (#21).

Supplementary Figure 11

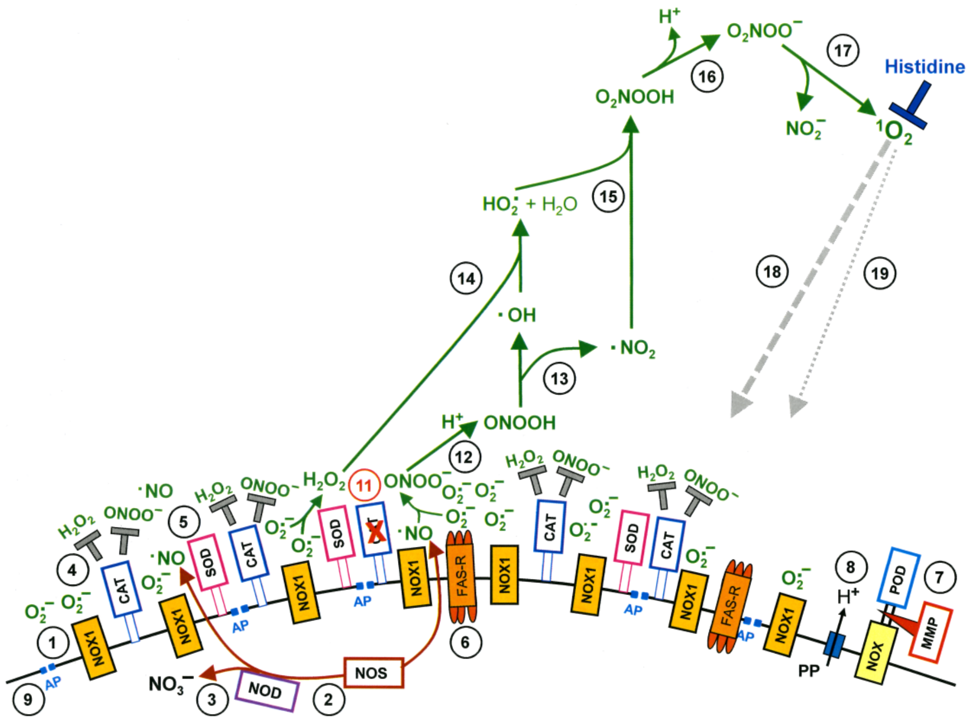

Legend to Suppl. Fig. 11: Scavenging of the secondary $^1O_2$ prevents further catalase inactivation and autoamplification.

Supplementary Figure 12

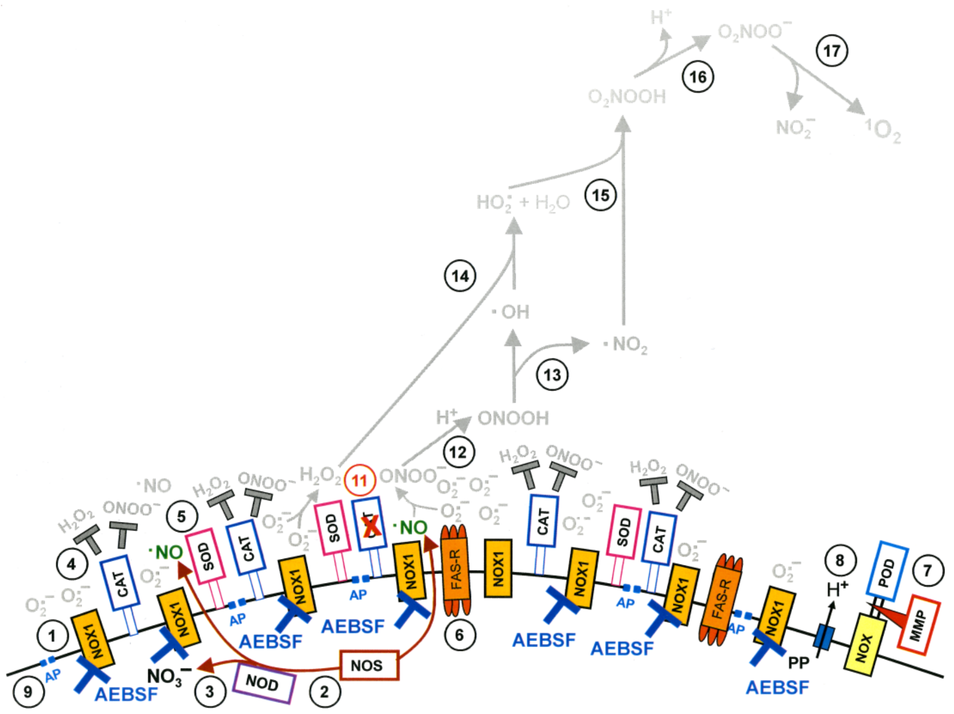

Legend to Suppl. Fig. 12: Inhibition of NOX1 by AEBSF prevents the generation of the secondary $^1O_2$

.

Supplementary Figure 13

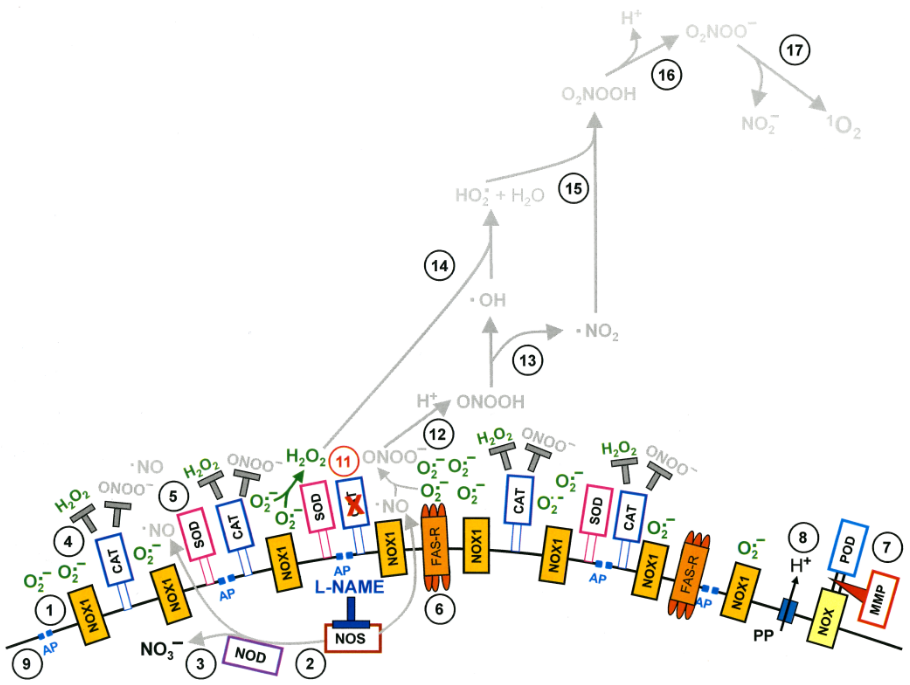

Legend to Suppl. Fig. 13: Inhibition of NOS by L-NAME prevents the generation of the secondary $^1O_2$.

Supplementary Figure 14

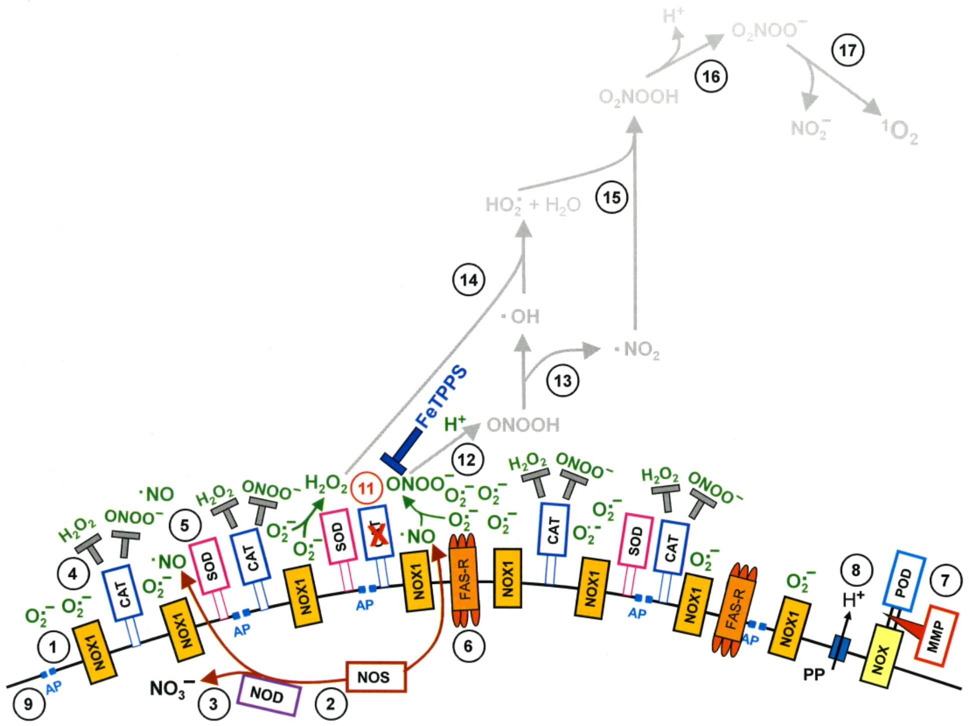

Legend to Suppl. Fig. 14: Decomposition of $ONOO^-$ by FeTPPS prevents the generation of the secondary $^1O_2$.

Supplementary Figure 15

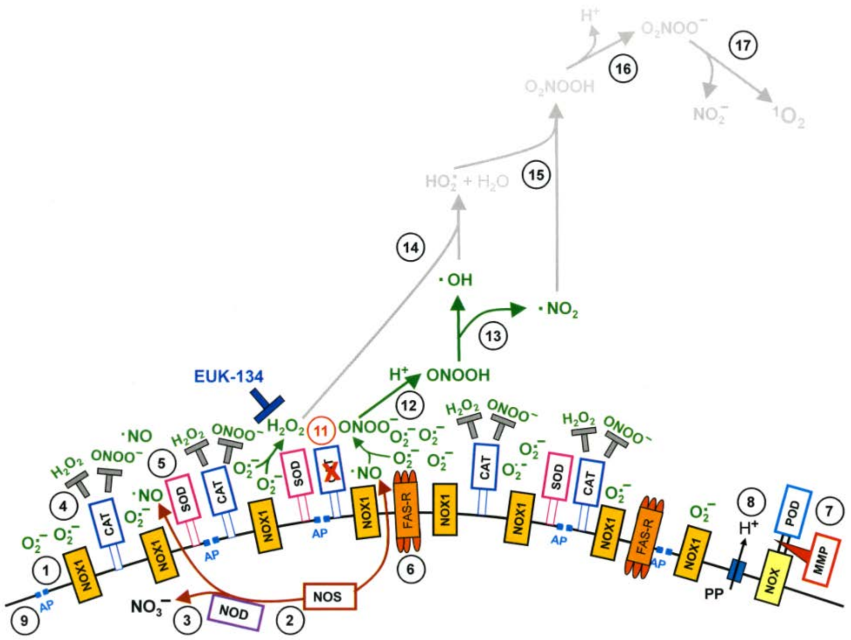

Legend to Suppl. Fig. 15: Decomposition of H_2O_2 by EUK-134 prevents the generation of the secondary $^1O_2$.

Supplementary Figure 16

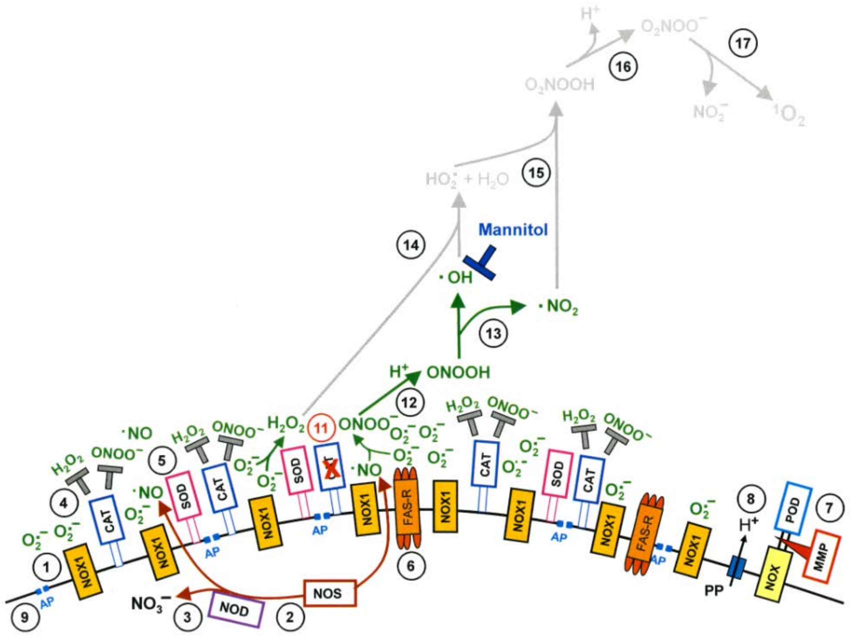

Legend to Suppl. Fig. 16: Scavenging of $^{\bullet}OH$ by mannitol prevents the generation of the secondary $^1O_2$.

B. 2 Visualization of the dynamic processes triggered by primary and secondary singlet oxygen ($^1O_2$)

Supplementary Figure 17 visualizes the steps between generation of primary $^1O_2$ generated through the interaction between H_2O_2 and NO_2^- derived from CAP and maintained in PAM, to local catalase inactivation, generation of secondary singlet oxygen up to apoptosis induction through intercellular RONS signaling. The generation of primary $^1O_2$ through the interaction between H_2O_2 and NO_2^- is limited and leads to the rare effect of inactivation of membrane-associated catalase. At the site of inactivated catalase, constant generation of H_2O_2 and $ONOO^-$ by the tumor cell (see Suppl. Figure 7 for details) leads to the sustained generation of secondary $^1O_2$. This establishes an autoamplification of $^1O_2$ generation and catalase inactivation. Finally, catalase inactivation is sufficient to allow HOCl signaling that causes apoptosis through the mitochondrial pathway of apoptosis.

Inactivated membrane-associated catalase on the surface of tumor cells acts as an imprint that will allow triggering of secondary $^1O_2$ in neighboring cells.

The transfer of few cells from a population with established autoamplification of catalase inactivation to untreated tumor cells is sufficient to trigger the onset of autoamplification of secondary $^1O_2$ generation in this cell population. The number of cells that are necessary to transfer bystander signaling can be taken as a measure for the frequency of imprinted cells.

When the treatment of tumor cells with CAP or PAM is performed in the presence of the NOX1 inhibitor AEBSF (Suppl. Figure 18), the imprint by primary $^1O_2$ is set, but the generation of secondary $^1O_2$ and its autoamplification are hindered. As AEBSF is a reversible inhibitor, it can be washed away. Its removal will cause the start of secondary $^1O_2$ generation and autoamplication. The kinetics of final apoptosis induction will be delayed compared to control assays without temporary AEBSF treatment. This scenario explains the kinetic differences seen in Figures 8-10 of the main manuscript.

Supplementary Figure 17

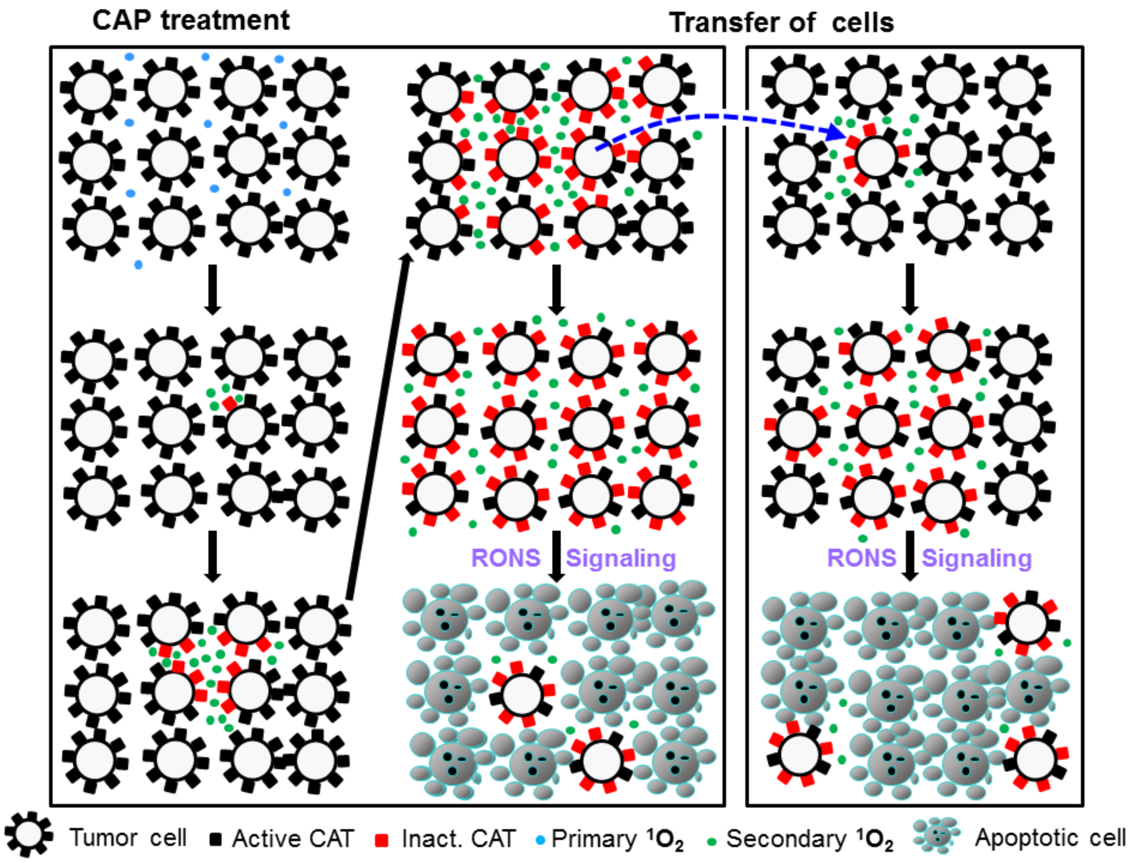

Legend to Suppl. Figure 17

Primary $^1O_2$ derived from the interaction between H_2O_2 and NO_2^- in CAP-treated medium (blue dots) has a small chance to hit tumor cells and inactivate a few catalase molecules (black squares) on their membrane. Inactivation of catalase is indicated by red squares. Its consequence is the sustained generation of the secondary $^1O_2$ (green dots) at the site of inactivated catalase, leading to the autoamplification of the secondary $^1O_2$ generation and catalase inactivation within the population. Finally, RONS-dependent apoptosis-inducing signaling is established and causes selective apoptosis induction in the tumor cells. The figure demonstrates the rare effect of the primary $^1O_2$, and the driving potential of the secondary $^1O_2$ generated by the tumor cells themselves.

Supplementary Figure 18

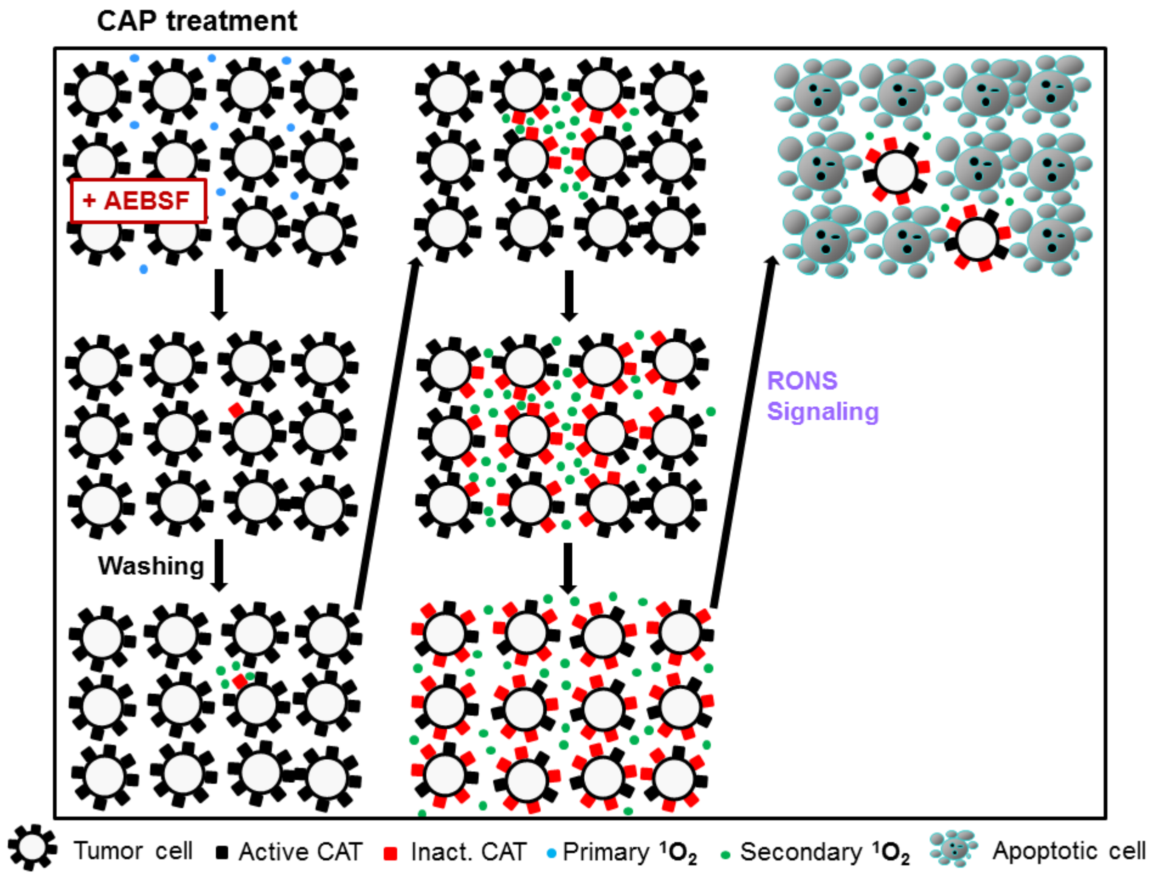

Legend to Supplementary Figure 18.
The transient inhibition of NOX1 by AEBSF, followed by a washing step, allows to demonstrate the dominant role of tumor cell-derived superoxide anions for the generation of secondary $^1O_2$ and for overall apoptosis induction. As a result, the kinetics of apoptosis induction is delayed, as shown in Figures 2-5 in the main manuscript.
Please find more details in the legend to Supplementary Figure 17 and in the text.

B.3 Dissection of the effects of the primary $^1O_2$ (derived either from the gaseous phase of CAP or generated through the reaction between H_2O_2 and NO_2^-) and the secondary $^1O_2$, generated by the tumor cells.

Tumor cells express active catalase on their membranes. When they are covered with medium and treated with CAP, they are confronted with the primary $^1O_2$ from the gaseous phase of CAP and with the primary $^1O_2$ generated through the interaction between H_2O_2 and NO_2^-, two long-lived species which are found in CAP-treated medium (**Suppl. Fig.19 A)**. Most of the primary $^1O_2$ from the gaseous phase of CAP will react with medium components on top of the assay, and only a few $^1O_2$ from this

source will reach tumor cells (in suspension) and inactivate catalase. H_2O_2 and NO_2^- will generate $^1O_2$ and a few $^1O_2$ molecules from this source will inactivate catalase. Our measurements showed that $^1O_2$ derived from H_2O_2 and NO_2^- is inactivating catalase more frequently than $^1O_2$ derived directly from the gaseous phase of CAP. At the site of inactivated catalase, secondary $^1O_2$ is constantly generated through the interaction between NOX1 and NOS of the tumor cells (Suppl. Fig. 19 B; see Suppl. Fig. 7 for details). In parallel, the concentration of H_2O_2 and also potentially NO_2^- is gradually decreased through the activity of membrane-associated catalase. Secondary $^1O_2$ generation is increased through the autoamplification and causes a bystander signaling within the population (C and D). This then allows intercellular apoptosis-inducing signaling through the HOCl signaling pathway (not shown in this figure). This figure (Supplementary Figure 19A) visualizes our findings on the rare effects of the primary and the high efficiency of the secondary $^1O_2$ generated by the tumor cells themselves. As the generation of the secondary $^1O_2$, as well as the intercellular HOCl signaling require active NOX1, and as NOX1 represents a hallmark of the malignant state, the selectivity of this process for tumor cells is warranted. For simplicity, the figure does not illustrate the influx of extracellular H_2O_2 into the tumor cells through aquaporins and the resulting depletion of intracellular glutathione. This effect sensitizes the target cells for apoptosis induction through the HOCl pathway.

Supplementary Figure 20 illustrates that the presence of the singlet oxygen scavenger histidine during CAP treatment and the subsequent incubation of the cells in the same medium completely prevents catalase inactivation, generation of secondary $^1O_2$ and apoptosis induction.

Supplementary Figure 21 demonstrates that the presence of the NOX1 inhibitor during CAP treatment and the subsequent incubation of the tumor cells in the same medium allows imprinting by primary $^1O_2$, but no generation of secondary $^1O_2$. After removal of AEBSF, secondary $^1O_2$ generation is started by the imprinted cells and autoamplification of $^1O_2$ generation, catalase inactivation and apoptosis induction are established in a delayed mode compared to control assays without AEBSF treatment.

Supplementary Figure 22 shows that the presence of the $ONOO^-$ decomposition catalyst FeTPPS during CAP treatment and the subsequent incubation of the cells in the same medium prevents the generation of the primary $^1O_2$ through the reaction between H_2O_2 and NO_2^-, whereas the primary $^1O_2$ derived from the gaseous phase

of CAP has a chance to imprint a few tumor cells through the inactivation of catalase. However, the generation of secondary $^1O_2$ is prevented by FeTPPS as well. Therefore, resumption of the secondary $^1O_2$ generation and its autoamplification can only start after FeTPPS has been removed from the system (D). As autoamplification and subsequent processes now start from very few initiation points, the resulting kinetics of apoptosis induction is strongly delayed, as has been shown in Figures 9 C, 10 C, 11 C and others of the main manuscript.

Supplementary Figure 19

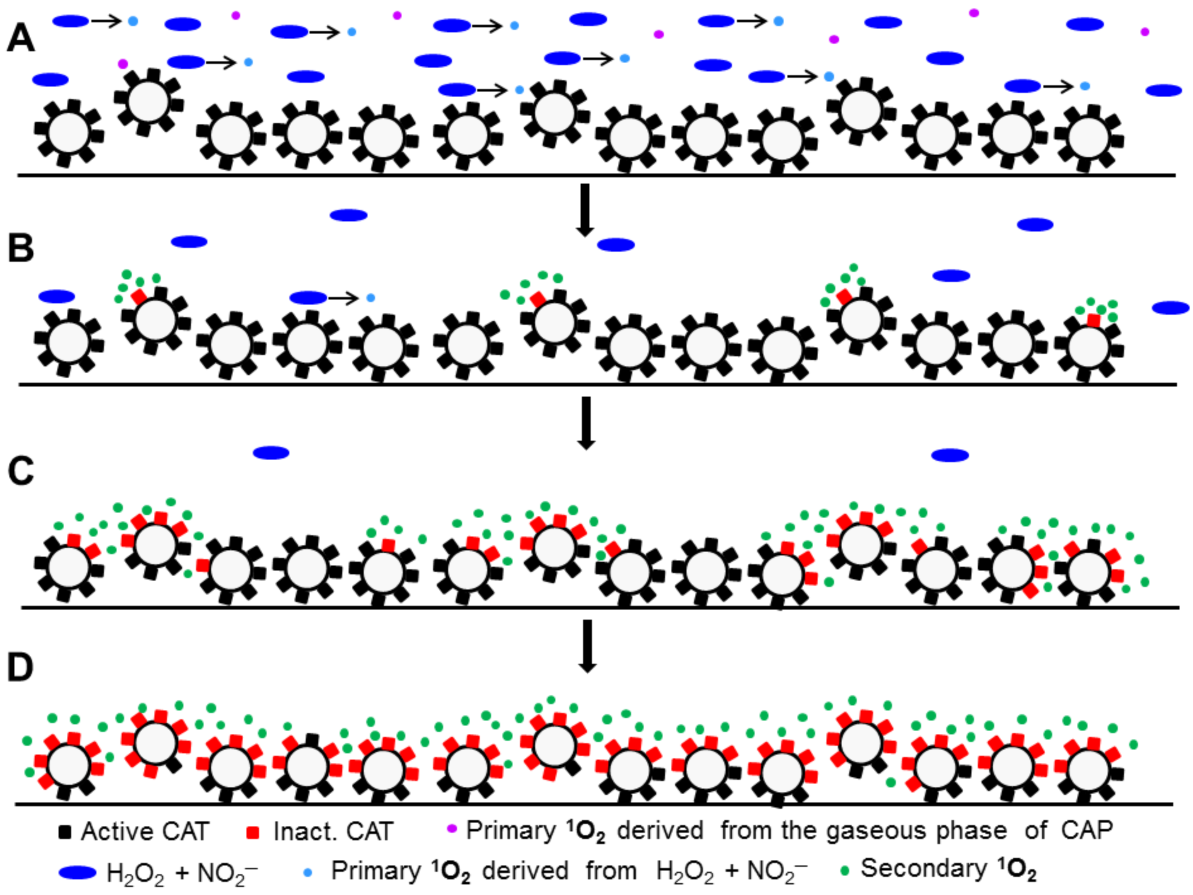

Supplementary Figure 19: Roles of primary $^1O_2$ from CAP or H_2O_2/NO_2^- interaction, and secondary $^1O_2$ generated by the tumor cells for inactivation of membrane-associated catalase .
A: As a consequence of treatment of tumor cells (in medium) with CAP, the cells are confronted with primary $^1O_2$ derived from the gaseous phase of CAP (pink dots) or generated through the reaction between the long-lived CAP-derived species H_2O_2 and NO_2^- (blue oval: H_2O_2 + NO_2^-, blue dot: $^1O_2$). As a rare event, membrane-associated catalase (black square) is hit by $^1O_2$. B: Secondary $^1O_2$ (green dots) is generated by the tumor cells at the site of inactivated catalase (red square). In parallel, the concentrations of H_2O_2 + NO_2^- are reduced by tumor cell catalase.
C, D: Secondary $^1O_2$ causes autoamplification of its generation and catalase inactivation. This leads to the reactivation of apoptosis induction through intercellular RONS signaling (not shown in the Figure).

Supplementary Figure 20

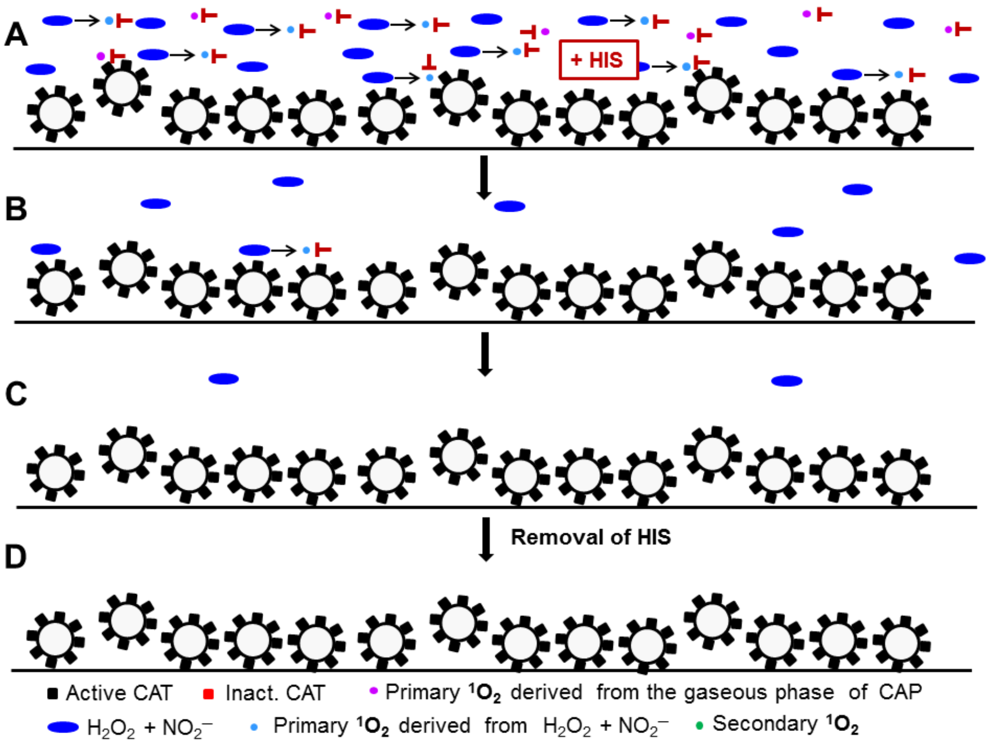

Suppl. Fig. 20: Scavenging of the primary $^1O_2$ by histidine (HIS) prevents the generation of the secondary $^1O_2$

Supplementary Figure 21

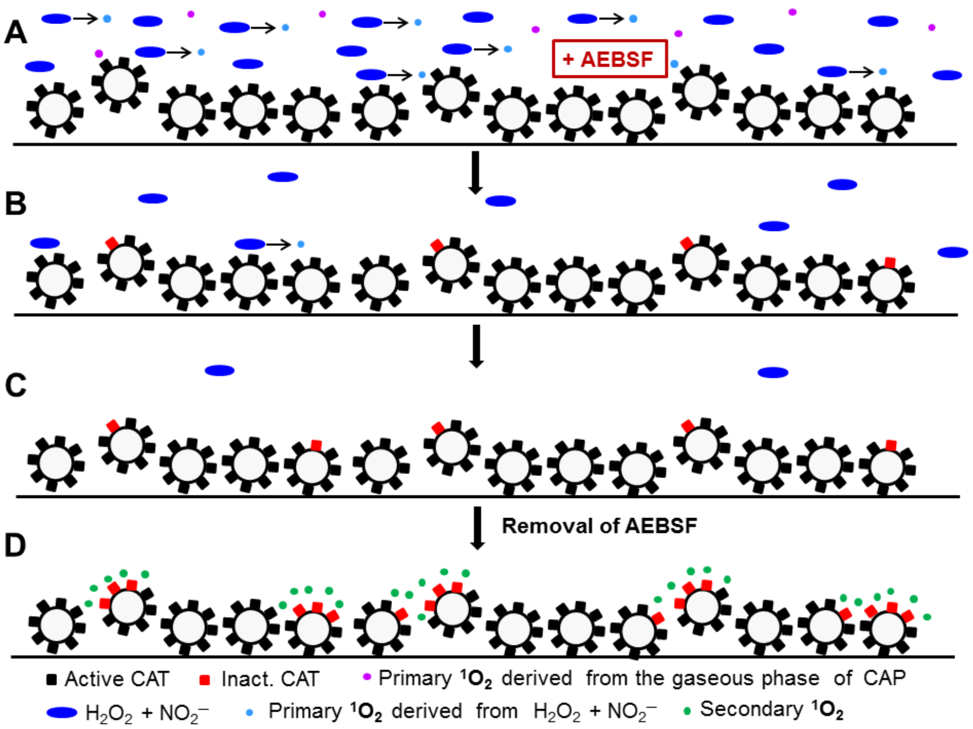

Suppl. Fig. 21: Transient inhibition of NOX1 by AEBSF allows imprinting by the primary $^1O_2$, but prevents the generation of the secondary $^1O_2$. After removal of AEBSF, the process continues in a delayed mode.

Supplementary Figure 22

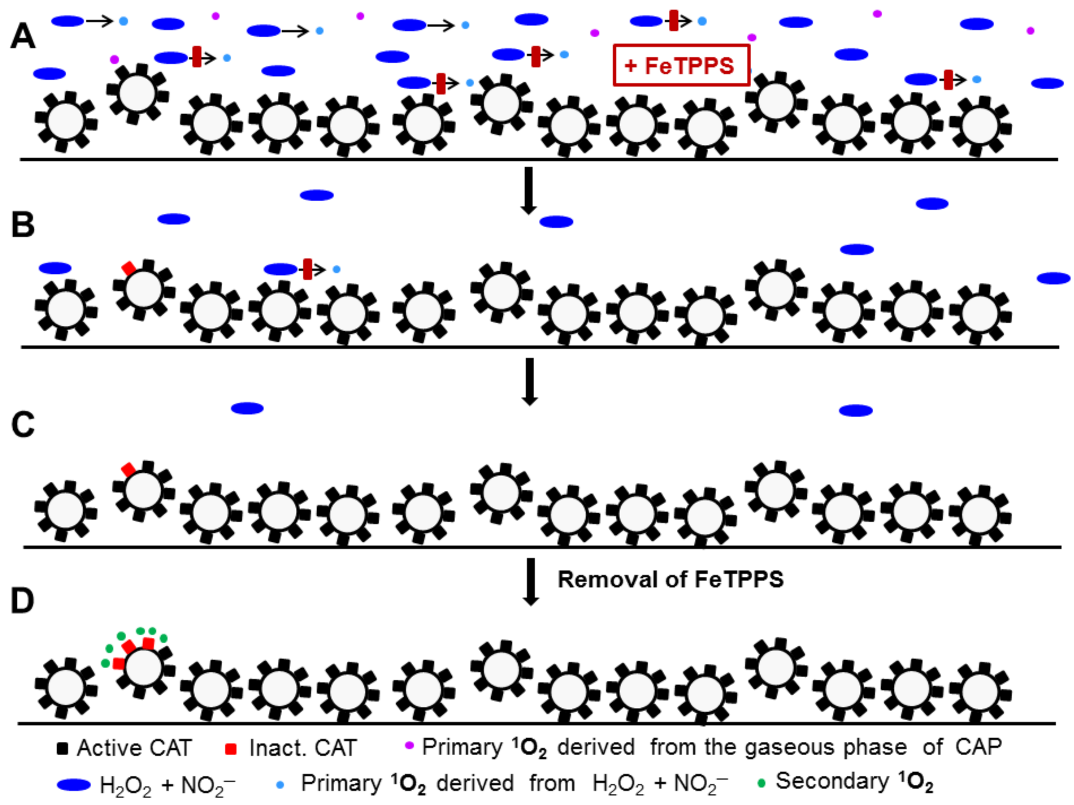

Suppl. Fig. 22: Transient decomposition of $ONOO^-$ by FeTPPS prevents the generation of primary $^1O_2$ derived from NO_2^- / H_2O_2 interaction, but allows imprinting by the primary $^1O_2$ derived from the gaseous phase of CAP.

B.4 Autoamplification of secondary $^1O_2$ generation on the initially triggered tumor cell and adjacent cells

The experimental proof for bystander effect-inducing potential of tumor cells with inactivated catalase triggered by $^1O_2$ has been established in the main manuscript through transfer of CAP- or PAM-pretreated tumor cells to a population of untreated tumor cells. It was then observed that the pretreated cells caused autoamplification of $^1O_2$ generation in the untreated population, leading finally to their apoptosis. No methods exist so far to directly demonstrate autoamplification of the $^1O_2$ generation on individual pretreated tumor cells themselves. However, a thorough reflection, as illustrated in Supplementary Figure 23, allows to conclude that the rare effect of imprinting, i. e. the catalase inactivation by $^1O_2$, first requires autoamplification of the secondary $^1O_2$ generation and catalase inactivation on the originally triggered cells,

before neighboring cells can be reached by $^1O_2$. The major reason for the strength of this statement is the extremely short free diffusion path length of $^1O_2$.

Supplementary Figure 23

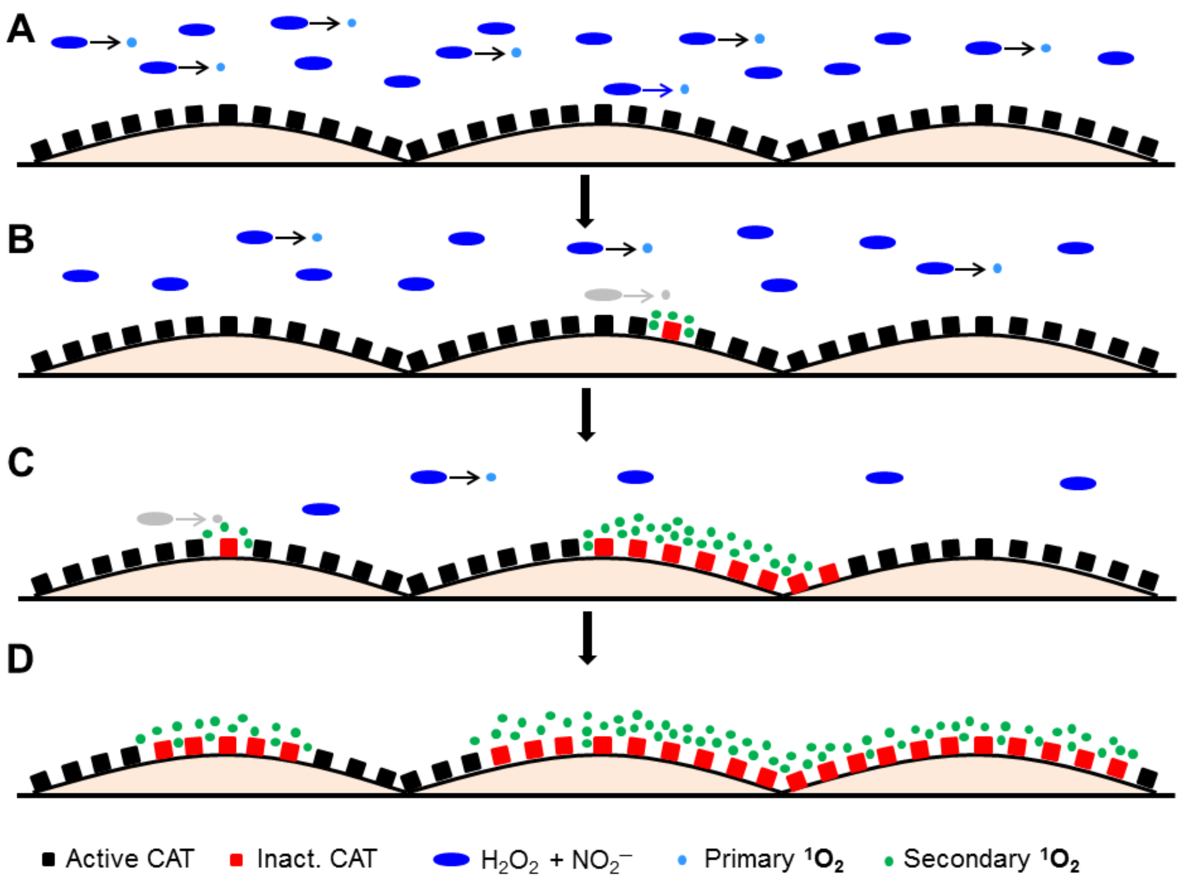

Supplementary Figure 23: Autoamplification of $^1O_2$ generation and catalase inactivation on the originally imprinted cell and on its neighbours
A: The generation of the primary $^1O_2$ (blue dot) from H_2O_2 and NO_2^- (blue oval) and its interaction with catalase (black square) on the membrane of tumor cells is a relatively rare effect. B. Secondary $^1O_2$ (green dot) is generated at the site of inactivated catalase (red square) through the interaction between tumor cell-specific NOX1 and NOS. C, D: Additional effects by the primary $^1O_2$ occur (cell on the left side), but become less frequent due to the decrease in the concentrations of H_2O_2 and NO_2^-. Autoamplification of secondary $^1O_2$ generation first needs to spread on the initially hit cell, before bystander signaling to neighboring cells can occur.

B.5 Detailed flow chart of CAP/PAM-mediated apoptosis induction in tumor cells

Supplementary Figure 24 illustrates three different ways to study the effects of long-lived RONS derived/generated by CAP. Part A 1 shows that "direct" treatment of cells (in medium) with CAP leads to the generation of short-lived species like $^{\bullet}OH$, $O_2^{\bullet -}$, O_3, $^{\bullet}NO$ and $ONOO^-$, as well as long-lived species like NO_2^- and H_2O_2. Whereas the short-lived species are rapidly quenched through the interaction with medium components, the long-lived species essentially remain (A2). Their interaction (see Supplementary Figure 7 for details) leads to the generation of $^1O_2$ which triggers the subsequent steps.

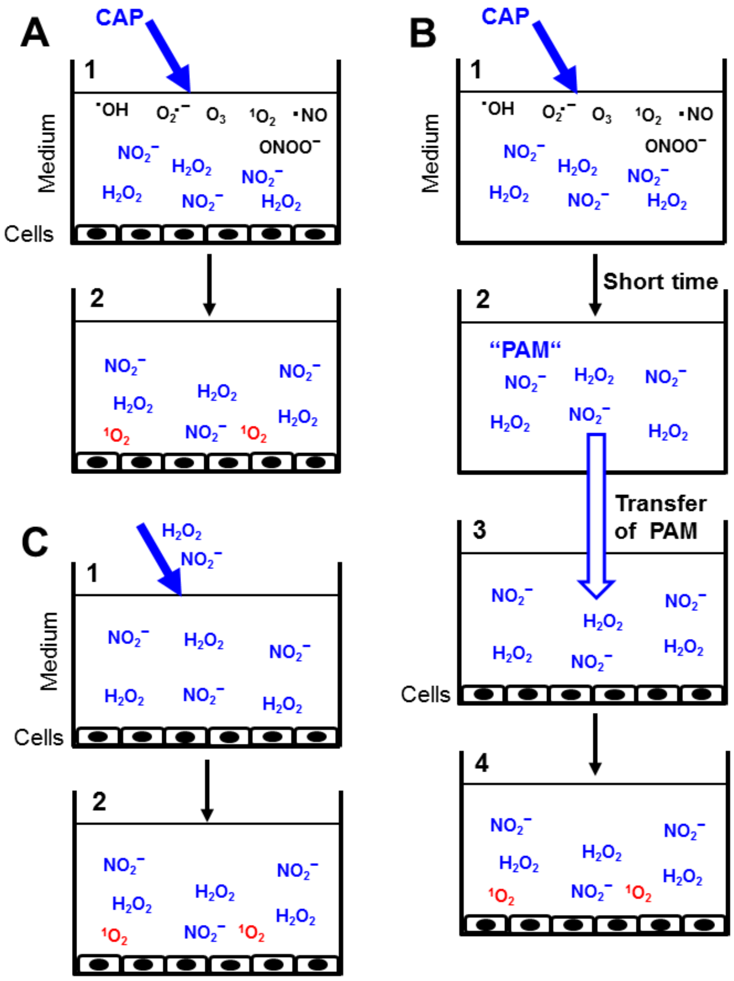

Suppl. Figure 24. Distinct modes to study the effects of CAP. Please find details in the text.

Treatment of the medium (without cells) with CAP (B1) leads to the same mixture of RONS as shown under A1. Within a short time (for some species microseconds are

sufficient), the short-lived species have been quenched (B2). Transfer of this plasma-activated medium (PAM) to cells allows the same subsequent steps as described under A2.

The effects of the long-lived species on tumor cells can be studied by adding defined concentrations of NO_2^- and H_2O_2 to tumor cells (C1,2). This approach has been performed in a parallel study (Bauer, 2019 a, b) and has allowed to study the signaling chemistry triggered specifically by these species. The results obtained are congruent to the findings obtained with direct application of a plasma source and with PAM generated by this source.

The flow chart (Supplementary Figure 25) starts with the long-lived species NO_2^- and H_2O_2 (#1) derived from either treatment shown in Supplementary Figure 24.

NO_2^- and H_2O_2 generate low concentrations of primary $^1O_2$ (#2) (according to the reactions outlined in Supplementary Figure 7). These are too low to cause cell death, but sufficient to trigger inactivation of a few catalase molecules on a few tumor cells within the population (#3). At the site of inactivated catalase, H_2O_2 and $ONOO^-$, steadily generated by NOX1 and NOS of the tumor cells, are no longer decomposed (#4) and thus ensure sustained generation of secondary $^1O_2$ (#5) through reactions shown in Supplementary Figure 7. Secondary $^1O_2$ causes further inactivation of catalase molecules on the originally triggered tumor cells and on their neighbors (#6). The cycling of this autoamplificatory process is of central importance for the strength and the selectivity of CAP and PAM-mediated apoptosis induction. As soon as membrane-associated catalase is inactivated at a sufficient degree in the tumor cell population, mainly cell-derived H_2O_2 intrudes into the cells through aquaporins (#7). This process causes depletion of intracellular glutathione *but is not sufficient by itself to cause apoptosis induction.* Glutathione depletion renders the cells sensitive for the apoptosis inducing effect of HOCl and/or $^{\bullet}NO/ONOO^-$ signaling (#8), as lipid

peroxidation through $^{\bullet}OH$ generated by both pathways at the membrane of the tumor cells can no longer be repaired by glutathione peroxidase-4 in the absence of an optimal glutathione concentration. Lipid peroxidation then triggers the mitochondrial pathway of apoptosis, which is executed by caspase-9 and caspase-3 (#9) and leads to the cell death (#10).

CAP and PAM-mediated apoptosis induction shows some additional side reactions which may provide for further enhancement and synergistic effects, as recently outlined (Bauer, 2019 c). As the FAS receptor can also be activated by $^1O_2$ (instead of its genuine ligand) (Zhuang et al.2001), primary and secondary $^1O_2$ also have a chance to activate the FAS receptor (#11) instead of interacting with catalase. In most tumor cell systems, this signal will not be sufficient to cause FAS receptor-mediated apoptosis (Bauer, 2015), but will induce strong enhancement of NOX1 and NOS activities (#12) (Suzuki et al., 1998; Reinehr et al., 2005; Selleri et al, 1997; reviewed in Bauer, 2015). The resultant increase in free $O_2^{\bullet -}$ and $^{\bullet}NO$ causes a profound enhancement of secondary $^1O_2$ generation and thus essentially enhances the autoamplificatory process of $^1O_2$ generation and potentially also the efficiency of subsequent apoptosis-inducing RONS signaling.

As SOD also carries an essential histidine residue in its active center (Escobar et al., 1996; Kim et al., 2001), primary or secondary $^1O_2$ may in some cases inactivate SOD instead of catalase (#13). This leads to free NOX1-derived $O_2^{\bullet -}$ at the site of inactivated SOD (#14). $O_2^{\bullet -}$ may inhibit catalase through formation of inactive compound III and thus lead to the presence of free H_2O_2 and $ONOO^-$, which may contribute to the generation of secondary $^1O_2$. Parallel inactivation of catalase and SOD leads to a synergistic effect, as determined in a parallel study that utilized neutralizing single domain antibodies directed towards catalase and SOD (Bauer and Motz, 2016). Therefore, the potential of $^1O_2$ to either inactivate catalase or SOD may

be one of the clues for the efficiency of CAP and PAM action towards tumor cells, as it bears an inherent potential for the induction of a valuable synergistic effect.

Suppl. Figure 25:

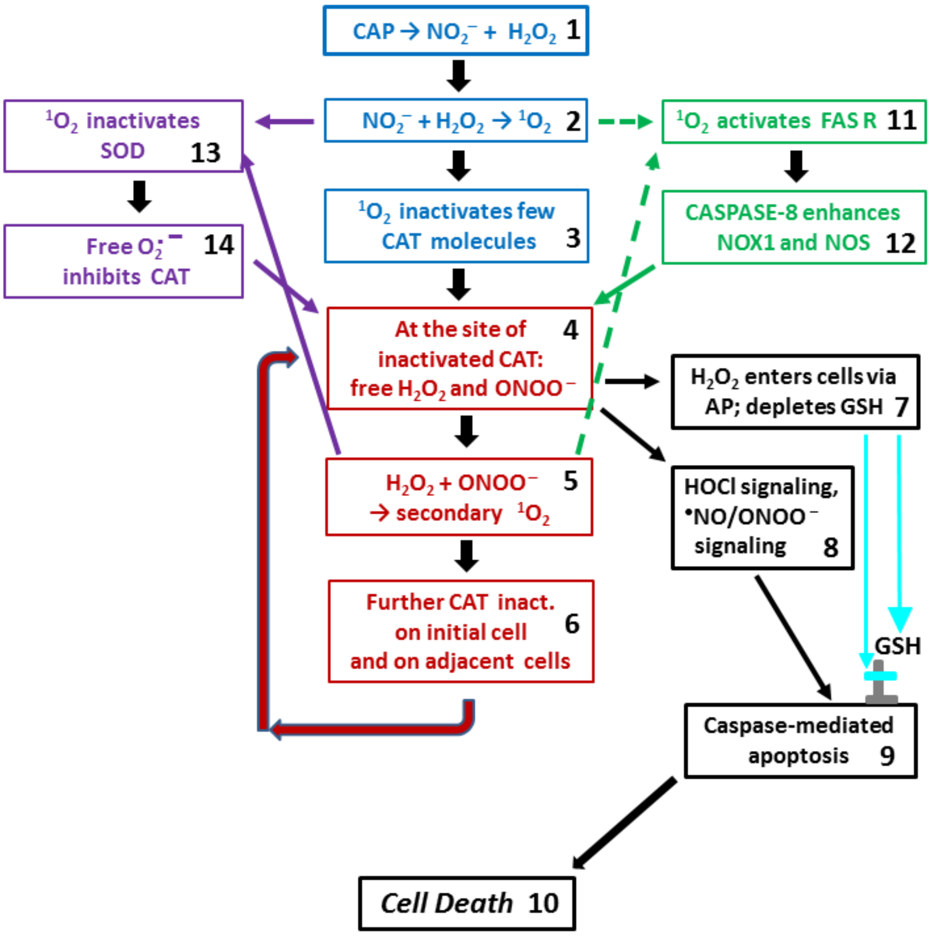

Suppl. Figure 25. Complete flow chart of apoptosis induction in tumor cells by CAP and PAM. Please find the explanation in the text.

B. 6 Comparison to other models for CAP and PAM-mediated apoptosis induction

The unique features of our model (established by experimental work using CAP, PAM, as well as reconstitution experiments with defined concentrations of NO_2^- and H_2O_2, and based on previous studies that utilized direct application of $^1O_2$ are:

a) the relatively rare generation of primary $^1O_2$ through the interaction between NO_2^- and H_2O_2 ,

b) inactivation of membrane-associated catalase on tumor cells by primary $^1O_2$, with the inherent selectivity towards tumor cells in this step,

c) sustained generation of secondary $^1O_2$ after catalase inactivation, with its inherent selectivity for malignant cells, due to essential participation of NOX1-derived $O_2^{\bullet -}$

d) the resultant autoamplification of these processes, driven by NOX1 and NOS of tumor cells;

e) apoptosis induction through intercellular RONS signaling, with its inherent selectivity for malignant cells, due to the central role of NOX1-derived $O_2^{\bullet -}$

f) and the counter control of the last step, which is based on aquaporins and cell-derived H_2O_2, thereby providing an additional step of selectivity control, as this H_2O_2 is the dismutation product of NOX1-derived $O_2^{\bullet -}$. As shown in this manuscript, CAP/PAM-derived H_2O_2 is not available for this step, as it is consumed by tumor cell catalase in the preceding time.

In this model, H_2O_2 from CAP and PAM is effective at concentrations that do no harm nonmalignant cells, which are more susceptible to H_2O_2 than tumor cells (Böhm et al., 2015). Initially, H_2O_2 plays no role by itself, but requires interaction with CAP/PAM-derived NO_2^- , to generate primary $^1O_2$. Later on, tumor cell-derived H_2O_2 is required for sustained generation of secondary $^1O_2$ through its reaction with

$ONOO^-$. After (at least partial) abrogation of catalase-mediated control, the influx of cell-derived extracellular H_2O_2 into the tumor cells is required to lower the concentration of intracellular glutathione – a necessary step, to sensitize the cells for apoptosis induction through $^{\bullet}OH$-mediated lipid peroxidation. The influx of H_2O_2 at concentrations that are generated by the tumor cells is, however, not sufficient to cause apoptosis induction through Fenton chemistry. Rather, H_2O_2 is used during intercellular HOCl signaling as the substrate for peroxidase to catalyze the generation of HOCl. The site-directed interaction between HOCl and NOX1-derived $O_2^{\bullet -}$, leading to $^{\bullet}OH$ at the membrane is then the apoptosis-inducing trigger. This finding is not only essential for the understanding of the established model. It also reminds us that the interaction between spatially defined $O_2^{\bullet -}$ with HOCl is a preferred principle in chemical biology to achieve site-specific $^{\bullet}OH$ generation, which would not be possible with randomly operating Fenton chemistry.

The models suggested by Yan et al. (2015a, 2017a) and Van der Paal et al., (2017) follow a completely different line, as they are based on the concept that CAP or PAM-derived H_2O_2 is the essential factor that determines selective apoptosis induction in tumor cells.

Yan et al. (2015a, 2017a) concluded that the increased concentration of aquaporins in the membrane of tumor cells, compared to nonmalignant cells, would result in an increased influx of H_2O_2 into the tumor cells and thus cause selective antitumor action.

Van der Paal et al. (2016) suggested that the decreased cholesterol content of tumor cells compared to nonmalignant cells was the determining factor for selective CAP and PAM action directed towards tumor cells, as cholesterol has a hampering effect on the ingress of ROS into cells.

Both models are based on the concept that ROS/RNS in CAP and PAM are ***directly*** responsible for the induction of cell death in the target cells. In both models, H_2O_2 is the major effector from CAP and the only effector from PAM. Both models did not consider, however, that tumor progression leads to a phenotype that is characterized by increased resistance to exogenous H_2O_2 (Deichman and Vendrov, 1986; Deichman et al., 1989. 1998; Deichman 2000, 2002). This tumor progression-associated resistance towards exogenous H_2O_2 is based on the expression of membrane-associated catalase (Heinzelmann and Bauer, 2010; Bechtel and Bauer, 2009 a, b; Böhm et al., 2015), Membrane-associated catalase not only protects tumor cells towards exogenous H_2O_2, but also oxidizes $^{\bullet}$NO and readily decomposes exogenous peroxynitrite ($ONOO^{-}$) (Heinzelmann and Bauer, 2010; Böhm et al., 2015). The models proposed by Yan et al. and Van der Paal et al. did not include an active role of tumor cells for their own cell death after CAP or PAM treatment, did not consider any involvement of tumor cell specific NOX1, and did not require the action of singlet oxygen to explain the cell death. Therefore, these models cannot explain our experimental findings on the requirement for active NOX1 and for the inactivation of membrane-associated catalase, followed by reactivation of intercellular RONS signaling. They also cannot mechanistically explain the bystander signaling potential of few CAP or PAM-pretreated cells towards an excess of untreated neighboring cells, as it was presented and analyzed in this manuscript.

However, the strong protective effect of aquaporin inhibition on apoptosis induction by PAM, as reported by Yan et al. 2015a, 2017a, opened the path for the understanding of aquaporin-mediated sensitization of tumor cells for exogenous apoptosis-inducing RONS signaling. Therefore, this central part of the concept established by Yan et al. 2015a, 2017a, has become an integral part of our model, with the modification that aquaporins on tumor cells are “gated” by membrane-

associated catalase. Therefore, their role for the influx of H_2O_2 into the cells cannot be established before membrane-associated catalase is inactivated. Further studies along these lines have shown that the aquaporin-mediated control effect as described by Yan et al. 2015 a, 2017 a, is a regular and central control element in RONS-mediated apoptosis induction, even beyond the action of CAP and PAM (Bauer, unpublished results). Other central findings by Keidar`s group are also consistent with our model: Yan et al., 2017 b described that CAP triggers a fast response of H_2O_2 generation by CAP-treated tumor cells. This effect seems to be consistent with the release of tumor cell-derived H_2O_2 from the control through membrane-associated catalase after CAP action. Likewise, the consumption of H_2O_2 through tumor cells as described by Yan et al., 2015 a, b is well explained by the effect of membrane-associated catalase. It is consistent with the finding described in Figure 19 of the main manuscript. Therefore, the model for CAP and PAM action, as proposed by us in this and the preceding manuscript also includes essential elements established by Keidar`s group.

Attri and Bogaerts (2019) have recently suggested an alternative model to the previous model explored by their group (Van der Paal et al., 2017). Their new model has adopted central elements of models described by Adachi et al., 2016; Yan et al., 2018 and Bauer (2018 b). Attri and Bogaerts follow Bauer`s concept that the interaction between long-lived species in PAM leads to the generation of $^1O_2$, which inactivates membrane-associated catalase of tumor cells. According to the model by Attri and Bogaerts, $^1O_2$ derived from PAM is sufficient to inactivate catalase to a degree that allows optimal influx of extracellular H_2O_2 through aquaporins, followed by apoptosis induction through Fenton chemistry. The weak points of this model are

the missing experimental proof-of-concept and the proposed model's apparent discrepancy with the experimental findings established in the original publications.

As shown in our study on the dynamics of $^1O_2$ generation by CAP and PAM, the generation of the primary singlet oxygen through the interaction between long-lived species is a relatively rare effect. It leads to apoptosis of tumor cells only if it is followed by the secondary singlet oxygen generation by the tumor cells. This key step in the model described in Bauer, 2018b, has not been incorporated into the composite model proposed by Attri and Bogaerts. Furthermore, our results obtained from multiple, separate experiments, including using CAP, PAM, defined concentrations of NO_2^- and H_2O_2, as well as with an exogenous source of singlet oxygen (Bauer 2019 a, b; Bauer et al, submitted, Riethmüller et al., 2015) are not consistent with the proposed composite model. These prior experiments uniformly show that apoptosis induction after inactivation of membrane-associated catalase of tumor cells always requires intercellular RONS signaling (HOCl or $^{\bullet}NO/ONOO^-$ signaling pathway) rather than a simple Fenton chemistry of H_2O_2. Multiple inhibitor and scavenging experiments showed that if RONS signaling were experimentally blocked after catalase inactivation, no apoptosis induction was observed. These observations were made under conditions such that Fenton chemistry would have been possible (Heinzelmann and Bauer, 2015; Scheit and Bauer, 2015; Riethmüller et al., 2015, Bauer, 2015; 2018 a, b, c, d). Moreover, the enhancement of Fenton chemistry through the addition of ferrous ion to an assay with ongoing HOCl-mediated apoptosis induction in malignant cells caused a drastic inhibition of apoptosis induction, due to the shift from site-directed to random generation of $^{\bullet}OH$ (Bauer, 2013).

Furthermore, as noted above, it is hard to see how models based on the concept that ROS/RNS in CAP and PAM are ***directly*** responsible for the induction of cell death in the target cells can explain autoamplification and the observed bystander effects. We note only a few 'activated' cells are sufficient to trigger a massive apoptotic response from a much larger group of non-activated cells. This is a central and strongly supported experimental effect. Any alternative model based on the aforementioned set of experiments would need to explain all of these observations.

B. 7 The potential connection between CAP-and PAM-mediated apoptosis induction and a subsequent specific immune response

One of the most exciting developments in oncology during the last years was the finding that classical tumor therapy, such as chemotherapy, radiotherapy, photodynamic therapy, and others, are not successful, unless they trigger a subsequent immunological attack on the treated tumor (Apetoh et al., 2008; Green et al., 2009; Golden and Apetoh, 2015; Krysko et al., 2012, Kroemer et al., 2013; Garg et al., 22014; Candeias and Gaipl, 2016). These findings have led to the concept of “immunogenic cell death (ICD)”, with its central theme that the activation of dendritic cells through defined signaling factors (“Damage-associated molecular patterns (DAMPs”) released by the dead cells leads to the activation of a cytotoxic T cell response. This T cell response is essential for the complete eradication of the tumor and can also act at sites in the body that are distant of the tumor, e. g. metastases

Hodge et al. (2013) have recognized that besides “classical ICD” (strictly defined by induction of cell death and by DAMPs), an “ immunogenic modulation” of tumor cells can enhance killing by cytotoxic T lymphocytes. This mechanism is distinct from classical immunogenic cell death but leads to an analogous result. Certain chemotherapeutics, which do not cause immunogenic cell death, have been shown

to have the potential for immunogenic modulation. In some cases, effective immunogenic modulation did not even require a preceding cell death, whereas in the case of immunogenic cell death, induction of the cell death is part of the definition. Consistent with the concept of immunogenic modulation, HOCl has been shown to enhance the recognition of antigens by dendritic cells, and thus to enhance the control of tumors by cytotoxic T cells, both in vitro and in vivo (Chiang et al., 2006, 2008, 2010, 2015; Prokopowicz et al., 2010; Biedron et al., 2015; Zhou et al., 2012). Several groups have shown that cold atmospheric plasma induces immunogenic cell death in vitro and in vivo (Miller et al., 2016; Lin et al., 2017 a, b, 2018, Mizuno et al., 2017; Bekeschus et al., 2018 a, b). ROS derived from plasma and/or generated within the treated cells seems to be involved in the triggering of ICD. In addition to the function of cytotoxic T cells, tumor cell killing by tumor necrosis factor type alpha from macrophages has been shown to be also involved in tumor cell killing after plasma treatment (Kaushik et al., 2018).

Therefore, it is an open and interesting question, whether RONS-dependent apoptosis induction, which is triggered by long-lived species derived from CAP or PAM and is established through autoamplificatory bystander signaling, is one of the triggers for ICD and/or immunogenic modulation. The answer to this question requires to clarify, whether defined species involved in intercellular RONS signaling are also inducers of DAMPs, or whether these processes are not directly connected. As apoptosis induction after CAP and PAM treatment requires the action of the HOCl signaling pathway (as shown in our present study), it is not unlikely that it also activates immunogenic modulation according to the mechanisms described by Chiang et al., Prokopowicz, Biedron and Zhou. In this way, reactivation of intercellular HOCl signaling through the CAP- and PAM-mediated autoamplificatory catalase inactivation might be the trigger for another, equally specific superimposed

immunological control system. In contrast to RONS-mediated apoptosis induction, which is dependent on an optimal cell density, the overlaid immunological process would have the power to attack individual tumor cells as well.

Supplementary References

Bauer,G. The synergistic effect between hydrogen peroxide and nitrite, two long-lived molecular species from cold atmospheric plasma, triggers tumor cells to induce their own cell death. *Redox Biol.* **26**, 101291 (2019a).
https://doi.org/10.1016/j.redox.2019.101291

Bauer, G.. Intercellular singlet oxygen-mediated bystander signaling triggered by long-lived species of cold atmospheric plasma and plasma-activated medium. *Redox Biol* .**26**, 101301 (2019 b).
https://doi.org/10.1016/j.redox.2019.101301

Adachi, T.; Nonomura, S.; Horiba, M.; Hirayama, T.; Kamiya, T.; Nagasawa, H.; Hara, H. Iron stimulates plasma-activated medium-induced A549 cell injury. *Sci. Rep.* **6**, 20928 (2016**).**

Apetoh, L., Tesnier, A., Ghiringhelli, F., Kroemer, G. & Zitvogel, L. Interactions between dying tumor cells and the innate immune system determine the efficiency of conventional antitumor therapy. *Cancer Res*. **68**, 4026-4030 (2008).

Attri, .P & Bogaerts, A. Perspectives of Plasma-treated Solutions as Anticancer Drugs. *Anticancer Agents Med Chem* **19**, 436-418 (2019).

Bauer, G & Motz, M. The antitumor effect of single-domain antibodies directed towards membrane-associated catalase and superoxide dismutase. *Anticancer Res*. **36**, 5945-5956 (2016).

Bauer, G. & Graves, D.B. Mechanisms of selective antitumor action of cold atmospheric plasma-derived reactive oxygen and nitrogen species. *Plasma Process. Polymer.* **13**,1157-1178 (2016).

Bauer, G. Signal amplification by tumor cells: clue to the understanding of the antitumor effects of cold atmospheric plasma and plasma-activated medium. *IEEE Transactions on Radiation and Plasma Medical Sciences* **2**, 87-98 (2018a)

Bauer, G. Targeting the protective catalase of tumor cells with cold atmospheric plasma-treated medium (PAM). *Anticancer Agents in Medicinal Chemistry* **18**, 784-804 (2018 b). DOI: 10.2174/1871520617666170801103708

Bauer, G. HOCl and the control of oncogenesis. *J. Inorganic Biochem*. **179**, 10-23 (2018 c). doi:10.1016/j.jinorgbio.2017.11.005

Bauer, G. Autoamplificatory singlet oxygen generation sensitizes tumor cells for intercellular apoptosis-inducing signaling. *Mech. Ageing Develop*. **172**, 59-77 (2018 d)

Bauer, G. HOCl-dependent singlet oxygen and hydroxyl radical generation modulate and induce apoptosis of malignant cells. *Anticancer Res*. **33**, 3589-3602 (2013).

Bauer, G. Increasing the endogenous NO level causes catalase inactivation and reactivation of intercellular apoptosis signaling specifically in tumor cells. *Redox Biol*. **6**, 353-371 (2015).

Bechtel, W. & Bauer, G. Catalase protects tumor cells against apoptosis induction by intercellular ROS signaling. *Anticancer Res*. **29***,* 4541-4557 (2009a).

Bechtel, W. & Bauer, G. Modulation of intercellular ROS signaling of human tumor cells. *Anticancer Res.* **29**, 4559-4570 (2009 b).

Bekeschus S, Clemen R, Metelmann H-R. Potentiating anti-tumor immunity with physical plasma. *Clin. Plasma Med*. **12**,17-22 (2018).

Bekeschus, S., Mueller, A., Miller, V., Gaipl, U. & Weltmann, K.-D. Physical plasma elicits immunogenic cancer cell death and mitochondrial singlet oxygen. *IEE transactions on Radiation and Plasma Medical Sciences*. **2**,138-147 (2018).

Biedron R, Konopinski MK, Marcinkiewicz J and Jozefowski S. Oxidation by neutrophil-derived HOCl increases immunogenicity of proteins by converting them into ligands of several endocytic receptors involved in antigen uptake by dendritic cells and macrophages. *PLOS ONE* **10**(4):e01123293 (2015).

Böhm, B., Heinzelmann, S., Motz, M. & Bauer, G. Extracellular localization of catalase is associated with the transformed state of malignant cells. *Biol. Chem*. **396**, 1339-1356 (2015).

Candeias, S.M. & Gaipl, U.S. The immune system in cancer prevention, development and therapy. *Anticancer Agents Med. Chem*. **16**, 101-107 (2016).

Chiang, C.L.L., Benencia, .F & Coukos, G. Whole tumor antigen vaccines. *Sem Immunol* **22**: 132-143 (2010).

Chiang, C.L.L., Coukos, G. & Kandalaft, L.E. Whole tumor antigen vaccines: where are we? *Vaccines* **3**: 344-372 (2015).

Chiang, C.L.L., Ledermann, J.A., Aitkens, E., Benjamin, E., Katz, D.R. & Chain, B.M. Oxidation of ovarian epithelial cancer cells by hypochlorous acid enhances immunogenicity and stimulates t cells that recognize autologous primary tumor. *Clin. Cancer Res*. **14**, 4898-4907 (2008).

Chiang, C.L.L., Ledermann, J.A., Rad, A.N., Katz, D.R. & Chain, B.M. Hypochlorous acid enhances immunogenicity and uptake of allogeneic ovarian tumor cells by dendritic cells to cross-prime tumor-specific T cells. *Cancer Immunol. Immunother*. **55**: 1384-1395 (2006).

Deichman, G. Early phenotypic changes of *in vitro* transformed cells during *in vivo* progression: possible role of the host innate immunity. *Sem. Cancer Biol*. **12**, 317-326 (2002).

Deichman, G. Natural selection and early changes of phenotype of tumor cells *in vivo*: Acquisition of new defense mechanisms. *Biochem*. ***65***, 78-94 (2000).

Deichman, G., Matveeva, V.A., Kashkina, L.M., Dyakova, N.A., Uvarova, E.N., Nikiforov, M.A. & Gudkov, A.V. Cell transforming genes and tumor progression: *in vivo* unified secondary phenotypic cell changes. *Int. J. Cancer* **75**, 277-283 (1998).

Deichman, G.I. & Vendrov, E.L. Characteristics of in vitro transformed cells essential for their in vivo survival, selection and metastatic activity. *Int. J. Cancer* **37**, 401-409 (1986).

Deichman, G.I., Kluchareva, T.E., Matveeva, V.A., Kushlinsky, N.E., Bassalyk, L.S. & Vendrov, E.L. Clustering of discrete cell properties essential for tumorigenicity and metastasis. I. Studies of syrian hamster embryo fibroblasts spontaneously transformed in vitro. *Int. J. Cancer* **44**, 904-907 (1989).

Escobar, J.A., Rubio, A. & Lissi, E. A. SOD and catalase inactivation by singlet oxygen and peroxyl radicals", *Free Radic. Biol. Med.* ***20***, 285-290 (1996).

Garg, A.D. & Agostinos, P. ER stress, autophagy and immunogenic cell death in photodynamic therapy-induced anti-cancer immune responses. *Photochem Photobiol Sci*. **13**, 474-487 (2014).

Golden, E.B. & Apetoh, L. Radiotherapy and immunogenic cell death. *Sem. Radiat. Oncol*. **25**, 11-17 (2015).

Green, D.R., Ferguson, T., Zitvogel, L. & Kroemer, G. Immunogenic and tolerogenic cell death. *Nature Rev Immunol.* **9**, 353–363 (2009).

Hodge, J.W., Garnett, C.T., Farsaci, B., Palena, C., Tsang, K.-Y., Ferrone, S. & Gameiro, S.R. Chemotherapy-induced immunogenic modulation of tumor cells enhances killing by cytotoxic T lymphocytes and is distinct from immunogenic cell death. *Int. J. Cancer* **133**, 624-637 (2013).

Kaushik, N.K., Kaushik, N., Min, B., Choi, K.H., Hong, Y.J., Miller, V., Fridman, A. & Choi, E.H. Cytotoxic macrophage-released tumour necrosis factor-alpha (TNF-alpha) as a killing mechanism for cancer cell death after cold plasma activation. *J. Phys. D-Appl. Phys*. **49**, 084001 (2018).

Kim, Y.K., Kwon, O. J. & Park, J.-W. Inactivation of catalase and superoxide dismutase by singlet oxygen derived from photoactivated dye. *Biochimie*. **83**, 437-444 (2001).

Kroemer, G., Galluzzi, L., Kepp, O. & Zitvogel, L. Immunogenic cell death in Cancer therapy. *Ann. Rev. Immunol.* **31**,51-72 (2013).

Krysko, D.V., Garg, A.D., Kaczmarek, A., Krysko, O., Agostinis, P. & Vandenabeele, P. Immunogenic cell death and DAMPs in cancer therapy. *Nature Rev. Cancer* **12**, 860-875 (2012).

Lin, A, Truong, B., Patel, S., Kaushik, N., Choi, E.H., Fridman, G., Fridman, A.& Miller, V. Nanosecond-pulsed DBD plasma-generated reactive oxygen species trigger immunogenic cell death in A549 lung carcinoma cells through intracellular oxidative stress. *Int. J. Mol. Sciences* ***18***, 966 (2017)

Lin, A., Truong, B., Fridman, G., Fridman, A. & Miller, V. Immune cells enhance selectivity of nanosecond-pulsed DBD plasma against tumor cells. *Plasma Medicine* **7**, 85-96 (2017)

Lin, A.G., Xiang, B., Merlino, D.J., Baybutt, T.R., Sahu, J., Fridman, A., Snook, A.E. & Miller, V. Non-thermal plasma induces immunogenic cell death in vivo in murine CT26 colorectal tumors. *Oncoimmunology* **7**, e148978 (2018).

Miller, V., Lin, A. & Fridman, A. Why target immune cells for plasma treatment of cancer. Plasma Chem. Plasm. Process. **36**, 259-268 (2016).

Mizuno, K., Yonetamari, Y., Shirakawa, Y., Akiyama, .T & Ono, R. Anti-tumor immune response induced by nanosecond pulsed streamer discharge in mice. *J. Phys. D-App.l Phys*. **50**, 12LT01 (2017).

Prokopowicz, Z.M., Arce, F., Biedron, R., Chiang, C.L.L., Ciszek, M., Katz, D.R., Nowakowska, M., Zapotoczny, S., Marcinkiewicz, J. & Chain, B.M. Hypochlorous acid: a natural adjuvant that facilitates antigen processing, cross-priming, and the induction of adaptive immunity. *J Immunol* **184**, 824-835 (2010).

Reinehr, R., Becker, S., Eberle, A., Grether-Beck, S. & Häussinger, D. Involvement of NADPH oxidase isoforms and src family kinases in CD95-dependent hepatocyte apoptosis. *J. Biol. Chem.* **280**, 27179-27194 (2005).

Riethmüller, M., Burger, N. & Bauer, G. Singlet oxygen treatment of tumor cells triggers extracellular singlet oxygen generation, catalase inactivation and reactivation of intercellular apoptosis-inducing signaling. *Redox Biol*. **6**, 157-168 (2015).

Scheit, K. & Bauer, G. Direct and indirect inactivation of tumor cell protective catalase by salicylic acid and anthocyanidins reactivates intercellular ROS signaling and allows for synergistic effects. *Carcinogenesis* ***36****,* 400-411 (2015).

Selleri ,C., Sato, T., Raiola, A.M., Rotoli, B., Young, N.S. & Maciejewski, J.P. Induction of nitric oxide synthase is involved in the mechanism of FAS-mediated apoptosis in hematopoietic cells. *Br. J. Hematol.* **99**, 481-489 (1997).

Suzuki, Y., Ono, Y. & Hirabayashi, Y. Rapid and specific reactive oxygen species generation via NADPH oxidase activation during FAS-mediated apoptosis. *FEBS letters.* **425,** 209-212 (1998*)*.

Van der Paal, J., Verheyen, C., Neyts, E.C. & Bogaerts, A. Hampering effect of cholesterol on the permeation of reactive oxygen species through phospholipid bilayer: Possible explanation for plasma cancer selectivity. *Sci. Rep*. **7**, 39526 (2017).

Yan, D.Y., Talbot, A., Nourmohammadi, N., Sherman, J.H., Cheng, X.Q. & Keidar, M. Toward understanding the selective anticancer capacity of cold atmospheric plasma- A model based on aquaporins. *Biointerphases.* **10**, 040801 (2015 a)

Yan, A., Talbot, N., Nourmokammadi, X., Cheng, J., Canady, J., Sherman, J.H. & Keidar, M. Principles of using cold atmospheric plasma stimulated media for cancer treatment. *Sci Rep.* **5**,1833901-18339017 (2015 b).

Yan, D., Xiao, H., Zhu, W., Nourmohammadi, N., Zhang, L.G., Bian, K. & Keidar, M. The role of aquaporins in the anti-glioblastoma capacity of the cold plasma-stimulated medium. *J. Phys. D-Appl. Phys.* **50***,* 055401 (2017 a).

Yan D, Cui H, Zhu W, Talbot A, Zhang LG, Sherman JH and Keidar M. The strong cell-based hydrogen peroxide generation triggered by cold atmospheric plasma Scientific Reports **7**: 10831, (2017 b**)**

Yan, D.; Sherman, J.H.; Keidar, M. The application of the cold atmospheric plasma-activated solutions in cancer treatment. *Anticancer Agents Med. Chem.* **18**, 769-775 (2018).

Zhou, R., Huang, W.-J., Ma, C., Zhou, Y., Yao, Y.-Q., Wang, Y.-X., Gou, L.-T., Chen, Y. & Yang, J.-L.: HOCl oxidation-modified CT26 cell vaccine inhibits colon tumor growth in a mouse model. *Asian Pacific J. Cancer Prevention* **13**, 4037-4043 (2012).

Zhuang, S., Demir, J.T. & Kochevar, I.E. Protein kinase C inhibits singlet oxygen-induced apoptosis by decreasing caspase-8 activation. *Oncogene* **20**, 6764-6776 (2001).
